# Effect of Conformational Variability on the Drug Resistance of *Candida auris* ERG11p and FKS1

**DOI:** 10.1021/acsomega.3c08134

**Published:** 2024-04-23

**Authors:** Hiroshi Izumi, Laurence A. Nafie, Rina K. Dukor

**Affiliations:** † National Institute of Advanced Industrial Science and Technology (AIST), AIST Tsukuba West, Tsukuba Ibaraki 305-8569, Japan; ‡ Department of Chemistry, Syracuse University, Syracuse, New York 13244-4100, United States; § BioTools Inc., Bee Line Hwy, Jupiter, Florida 33458, United States

## Abstract

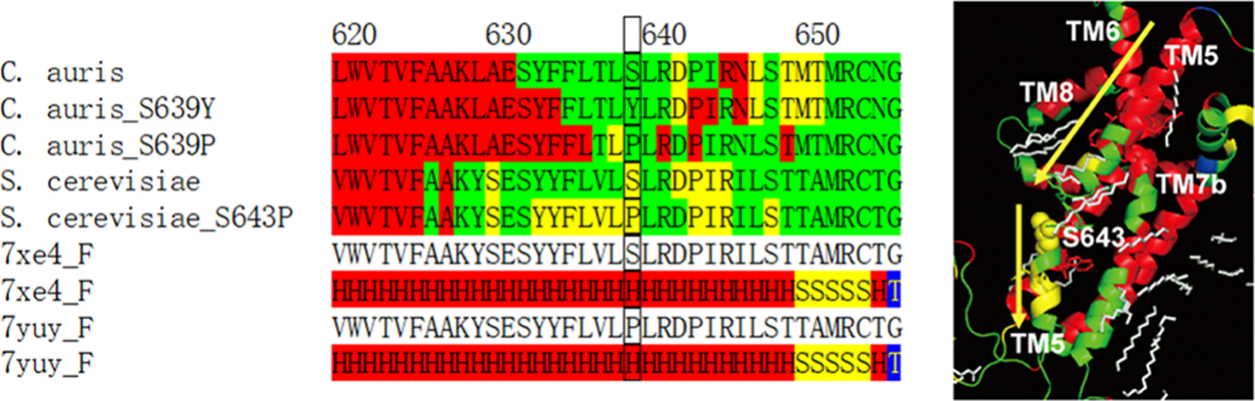

*Candida auris* infection has been recognized as an urgent threat to antifungal drug resistance, and the Eagle effect of *C. auris* FKS1 (1,3-β-d-glucan synthase) wild-type isolates has also been noted. The Eagle effect, namely, where higher concentrations of antifungals reduce fungicidal activity relative to lower concentrations, is a confounding factor of apparent antifungal resistance, but the detailed mechanism remains unclear. Here, we present the conformational variability of mutation sites for ERG11p (lanosterol 14α-demethylase) and FKS1 from deep neural network-based prediction along with the reported X-ray crystallographic and cryo-electron microscopy (cryo-EM) structures of antifungals. The sequence variability maps provide valuable insights into the inconsistent correlation between azole resistance and the mysterious Eagle effect with the dispersion of minimal inhibitory concentration (MIC) for echinocandin resistance. The conformational variability prediction supports the hypothesis that mutations K143R of clade I, VF125AL of clade III, and Y132F of clade IV for *C. auris* ERG11p make the corresponding site variable and that an increased population of invisible variable conformations potentially contributes to triazole resistance. In contrast, the predicted rigid conformation by the S639F mutation of hot spot region 1 (HS1) for FKS1 suggests that caspofungin (CAS) is involved in an uncompetitive inhibition, and a decreased population of the CAS-bound state of FKS1 with Rho1 leads to drug resistance. The predicted variable HS1 region for FKS1 WT isolates and the rigid one for FKS1 S639F mutants support the in vivo drug response and the in vitro MIC dispersion. A plausible mechanism of the Eagle effect is hereby proposed, namely, that a high concentration of CAS with a high membrane affinity reduces the population of the CAS-bound state of FKS1 with Rho1, as well as accompanying events such as aggregation or association depending on the conformational variability of HS1.

## Introduction

**Figure 1:**
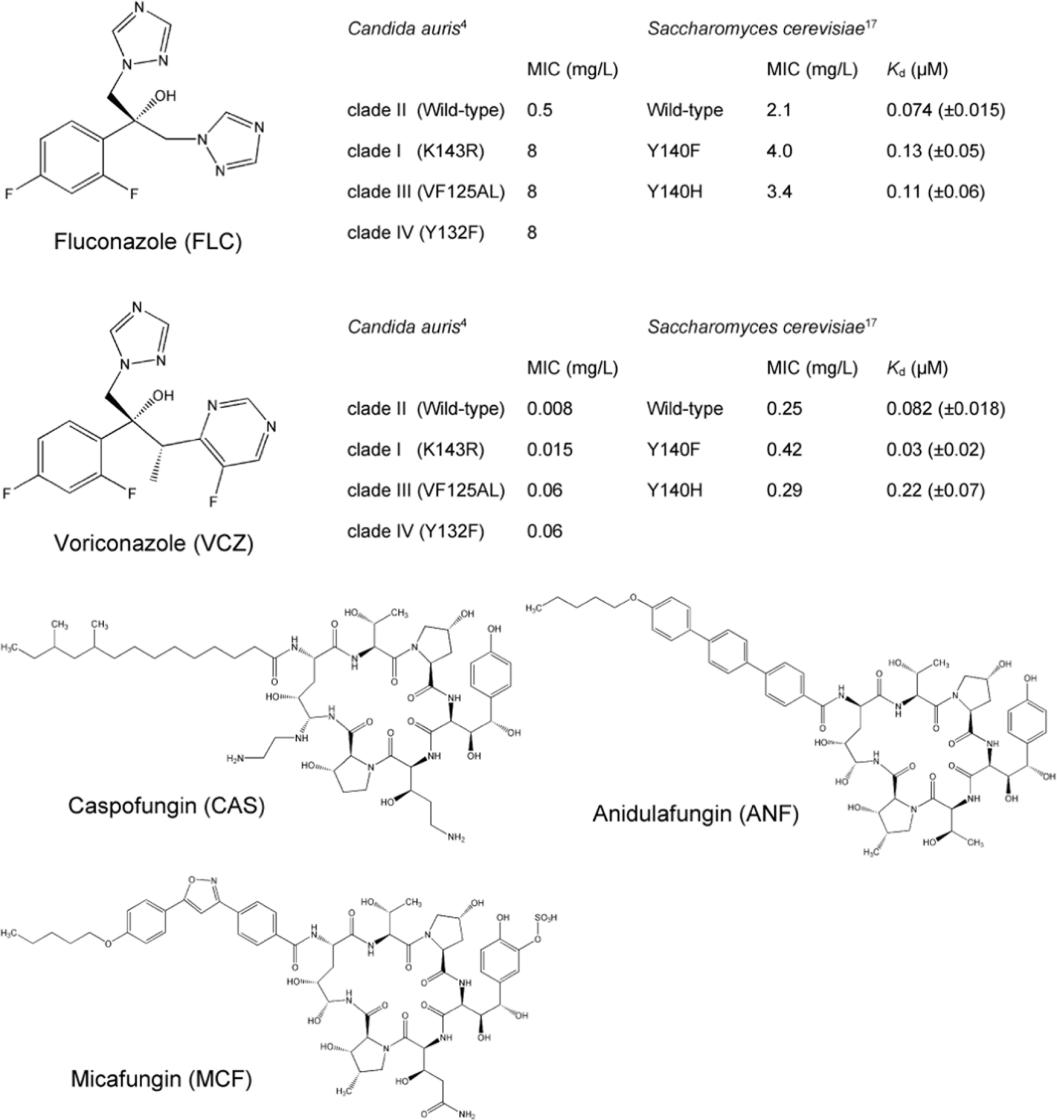
Chemical structures of representative azole and echinocandin drugs referenced in this paper. Also provided are the correlation data of the azoles FLC and VCZ between drug resistance, MIC (minimal inhibitory concentration), and dissociation constant, *K*_d_, for *C. auris* and *Saccharomyces cerevisiae*.^4,17^ Tables were originally created by using the quoted data.

*Candida auris* infection has been recognized as an urgent threat from the centers for disease control and prevention (CDC) and is a new fungal member among emerging infectious diseases.^1,2^*C. auris*, which was first isolated from ear discharge in Japan in 2005 (reported 2009),^3^ is resistant to high temperature and high osmotic pressure. In addition, there are four major clades of *C. auris* phylogenetically,^4,5^ in which the three clades except for the East Asia clade (clade II) are mainly associated with multidrug resistance (Figure 1) and outbreaks.^6−8^ The phenomenon that higher concentrations of antifungals have reduced fungicidal activity compared to lower ones is known as an Eagle effect.^9^ In many reports of the Eagle effect, the concentrations causing reversal were represented as the multiple of the minimal inhibitory concentration (MIC)^10^ but not for *C. auris*.^9^ The Eagle effect of *C. auris* FKS1 (1,3-β-d-glucan synthase) wild-type (WT) isolates, which correlates with the apparent antifungal resistance, has been noted, but the detailed mechanism has not been clarified.^9^

Techniques using deep neural network such as Alphafold,^11^ Rosettafold,^12^ and ProteinMPNN^13^ have predicted highly faithful protein structures and have made a strong impact on molecular biology. Recently, we presented a deep neural network-based conformational variability prediction system of protein structures (SSSCPreds)^14^ and the high prediction reliability of the correlation between conformational variability and phenotypes by mutations of spike proteins for SARS-CoV-2 variants.^15,16^ We have proposed the supersecondary structure code (SSSC), which is represented as a conformation propensity using the letters “H”, “S”, “T”, and “D” for each amino acid peptide unit referring to an α-helix-type conformation (H), a β-sheet-type conformation (S), a variety of other-type conformations (T), and disordered residues or the C-terminus (D), and this code has been authorized as a protocol for molecular biology databases.^16^ For SSSCPreds, the conformational variability for each amino acid peptide unit is evaluated by using the agreement of SSSCs among independent SSSCPred200, SSSCPred100, and SSSCPred (three neural networks of SSSCs including different lengths of protein data bank (PDB) amino acid sequences) and categorized as the following conformations: a variable conformation (green), an α-helix-type conformation (red), a β-sheet-type conformation (yellow), and a variety of other-type conformations (blue).^16^

SSSCPreds can concurrently predict sites of protein variability and the shapes of those conformations including loop structures with high accuracy.^16^ The value size of concordance rates for SSSC sequences among the three systems of SSSCPreds provides a good indication of the variability of the protein subunits and can be useful for predicting the ease of obtaining crystals.^14^ We have reported a high correlation between conformational variability of the receptor binding domain (RBD) for the SARS-CoV-2 spike protein and the actual expression or angiotensin-converting enzyme 2-binding affinity.^14^ Further, the difference of seasonable pandemic variants in summer and those in winter including Omicron variants XBB.1.5, EG.5.1, BA.2.86, and JN.1 can be distinguished by this conformational variability.^16^

We note that SSSCPreds compares supersecondary structure prediction such as a helix–hairpin–helix motif and not secondary structure prediction. SSSC for protein structure prediction is completely different from the assignment for protein secondary structure prediction using DSSP (database of secondary structure assignments program). As further support of the supersecondary structure prediction capability for SSSCPreds, the following facts are noted. A subunit with the keyword PHOSPHOGLYCERATE MUTASE 1 (3pgm_A) indicated a low concordance rate (0.62) between SSSCPred200 and PDB data, but the PDB files (1qhf_A, 1bq3_A, 4pgm_A, and 5pgm_A) for the identical amino acid sequence indicated high concordance rates (0.96, 0.97, 0.96, and 0.99) between SSSCPred200 and PDB data, so that SSSCPreds predicted a high concordance rate (0.91) between SSSCPred200 and SSSCPred100 data.^14^ The increase in the number of PDB files for the identical amino acid sequence with low concordance rates decreases the predicted concordance rate between SSSCPred200 and SSSCPred100. In this way, SSSCPreds can predict the conformational variability of proteins from more than 350,000 subunit random data, including NMR data.

In addition, the predicted conformational variability of the subunits with the keywords PROTEASOME, FAB, LYSOZYME, HEMOGLOBIN, MICROGLOBULIN, HLA, MYOGLOBIN, GLUCOSIDASE, OUTER MEMBRANE, ENVELOPE, PORIN, REPLICATION, INTERLEUKIN, RIBOSOMAL PROTEIN, KINASE, TRANSFERASE, SYNTHASE, REDUCTASE, DEHYDROGENASE, HYDROGENASE, POLYMERASE, HYDROLASE, PROTEASE, PHOSPHATASE, ISOMERASE, OXIDASE, and so on has been extensively characterized with the observed SSSC sequences by X-ray crystallography, cryo-electron microscopy (cryo-EM), and NMR spectroscopy.^14^ Beyond this, SSSCPreds reproduces the difference of RBD between SARS-CoV and SARS-CoV-2^14^ and the conformational changes of the HIV-1 envelope glycoprotein with fluorescence resonance energy transfer (FRET) data, avian influenza A (H5N1) hemeagglutinin, *Staphylococcus aureus* DNA gyrase A, Piezo1, TRPV1, CAS9, and so on.

X-ray crystallographic and cryo-EM structures potentially provide valuable conformational information about catalytic actions, such as processive polysaccharide synthases, but these techniques are not entirely perfect. In most cases, the actual active conformations are not visible, in principle. The conformational variability prediction can complement the catalytic transformation information in these structural analyses. For azole resistance, the apparent lack of correlation between resistant phenotypes and azole affinity for *S. cerevisiae* as a target surrogate has been reported (Figure 1).^17^ In lipopeptide echinocandin drugs, only caspofungin (CAS) shows the remarkable Eagle effect with the dispersion of MIC.^9^ To define the inconsistent correlation between azole resistance and the mysterious Eagle effect associated with echinocandin resistance considering the effect of conformational variability, we analyzed sequence variability maps using SSSCPreds with the reported X-ray crystallographic and cryo-EM structures of antifungals.

## Results and Discussion

### Azole Resistance

**Figure 2:**
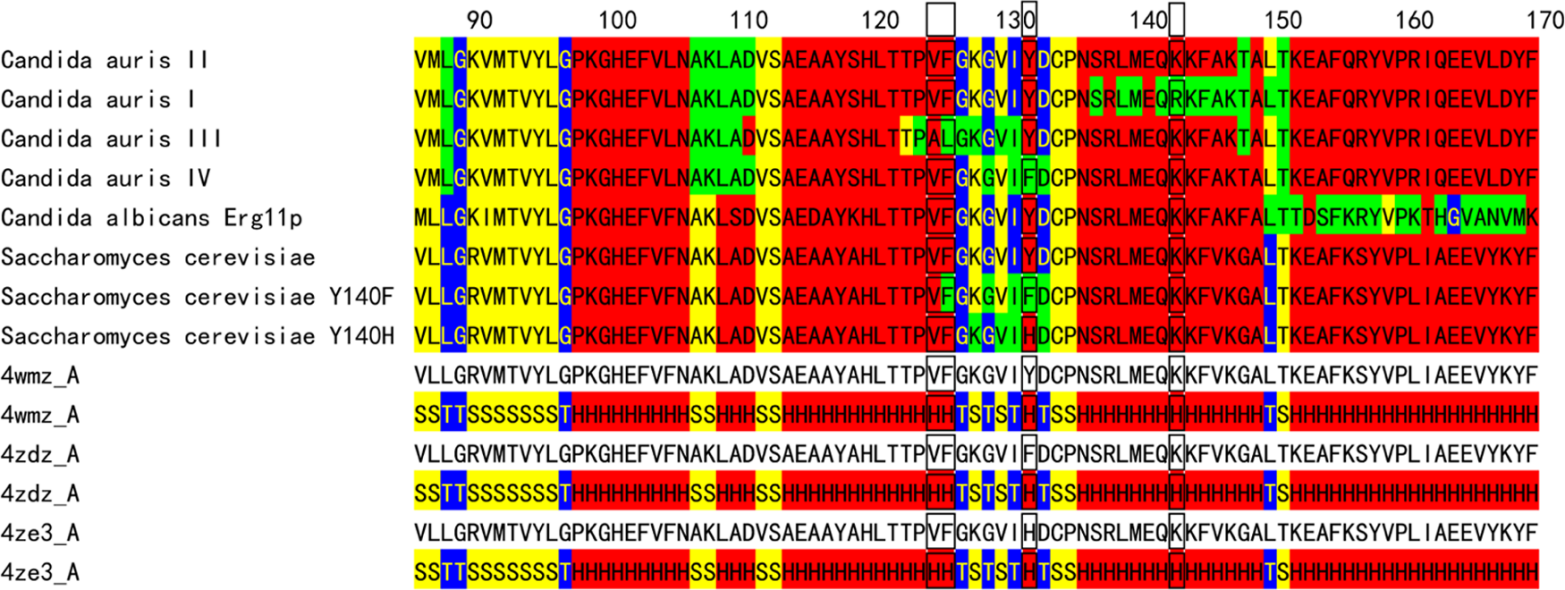
Sequence variability maps of mutation sites (Erg11p) for clades I–IV of *C. auris*, *C. albicans*, and *S. cerevisiae* with supersecondary structure code (SSSC) maps of PDB structures (green, variable conformation; red, α-helix-type conformation; yellow, β-sheet-type conformation; blue, other-type conformations; black rectangles, mutation sites).

**Figure 3:**
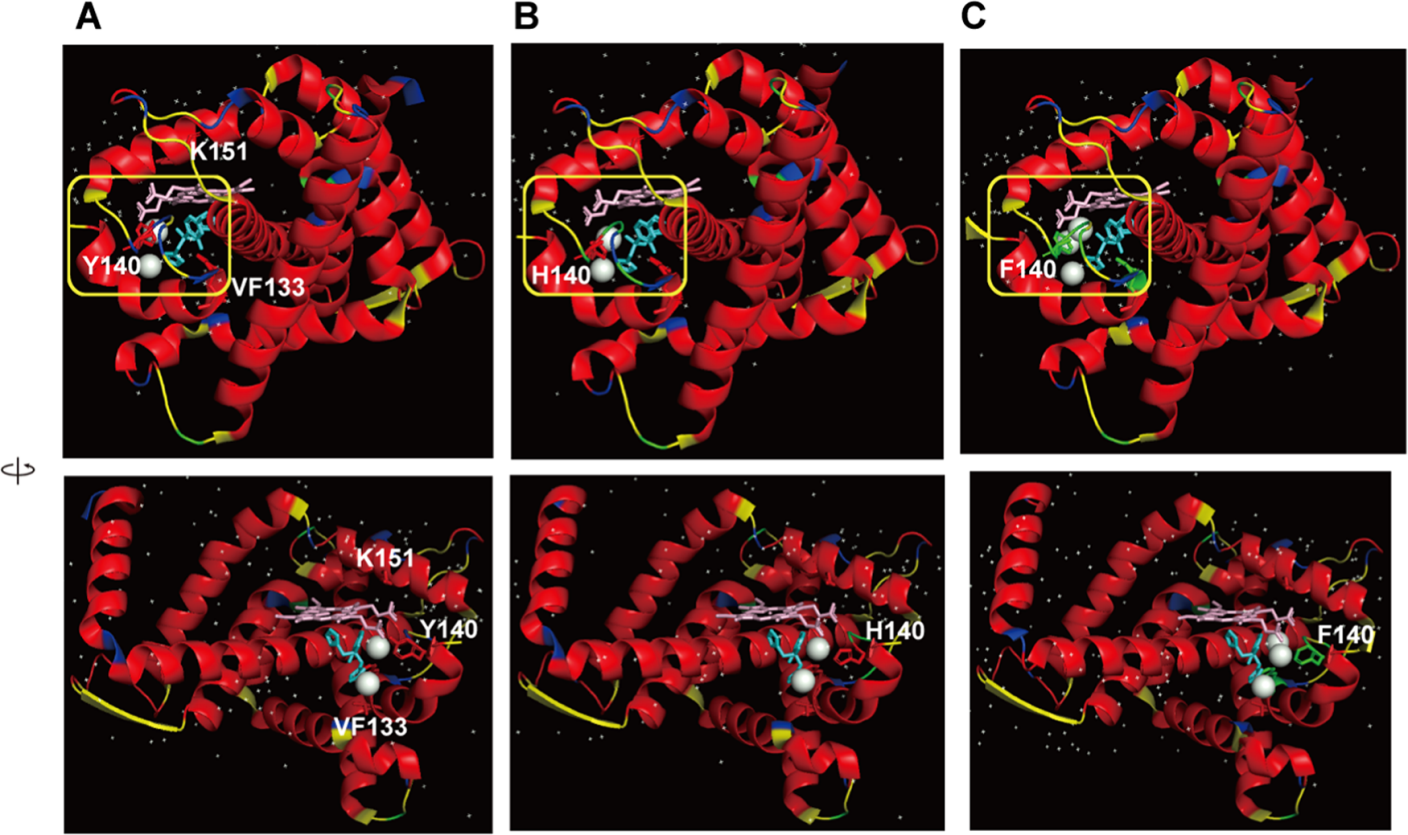
Conformational variability maps front and back (green, variable conformation) of mutation sites (yellow rectangular frame and stick structures) for *S. cerevisiae* on PDB ID 4wmz (A), on PDB ID 4ze3 (B), and on PDB ID 4zdz (C) with FLC (cyan) and heme (pink). The water molecules in the hydrogen-bond network are represented as white spheres.

*C. auris* ERG11p (lanosterol 14α-demethylase) is the enzymatic target of azoles.^17^ Although the conformational variability of ERG11p among *C. auris*, *Candida albicans*, and *S. cerevisiae* at the sites such as residues 35–70, 170–205, and 265–285 is quite different from their predicted variability maps, conformational variability at the target sites (residues 120–145) is identical (Figure S1) because of the architecture of the heme-containing protein. Further, the predicted conformational pattern at the target sites completely reproduces that of the X-ray structures (Figure 2 and yellow rectangular frame in Figure 3). As shown above, the value size of the concordance rates for SSSC sequences among the three systems of SSSCPreds provides a good indication of the variability of the protein subunits.^14^ In general, the ratios of hemoglobin (760/825) and myoglobin (174/178) PDB files with the concordance rate of ≥0.90 to the total number of those files are extremely high (≥0.92), making the architecture of heme-containing proteins very rigid.^14^ The concordance rates of the entire ERG11p subunit for *C. auris* were 0.77–0.81, and those for *C. albicans* were 0.73–0.77. On the other hand, those for *S. cerevisiae* were 0.88–0.98, and the value size of concordance rates for *S. cerevisiae* was suitable to obtain the crystal for X-ray crystallographic analysis.

The three clades, clade I (originally associated with South Asia, K143R), clade III (originally associated with South Africa, VF125AL), and clade IV (originally associated with South America, Y132F), except for clade II (originally associated with East Asia), have clear antifungal drug resistance.^4^ The predicted variability map of clade II near the mutation sites (residues 120–145) shows a rigid site containing the characteristic conformational pattern of the loop structure near the heme (Figure 2 and yellow rectangular frame in Figure 3). On the other hand, the mutations K143R, VF125AL, and Y132F make each site of clades I, III, and IV variable, respectively. Sagatova and co-workers have reported that the [Azole]_0.5_ and *K*_d_ values obtained from plotting the change in absorbance against the azole concentration with the WT and mutant enzymes for each triazole drug indicated comparably high-affinity binding.^17^ They also showed the X-ray crystal structures of *S. cerevisiae* Erg11p (ScErg11p) as a target surrogate (Figure 3). The structures of WT ScErg11p6 × His and the Y140F mutant enzyme complexed with fluconazole (FLC) show the drug to be bound in a similar conformation, with all differences in the interactions between the drug and the enzyme ascribed to the mutation.^17^ From these findings, they have claimed that a water-mediated hydrogen-bond network involved in binding of short-tailed triazoles, which is disrupted by the mutations, leads directly to a decrease in the binding affinity and then FLC and voriconazole (VCZ) resistance.^17^ However, this interpretation does not explain the comparable high affinity binding results obtained from the absolute absorption spectra. Further, the other hot spot (HS) of K143R for clade I of *C. auris* (ScErg11p: K151R) is placed on the opposite side of the heme ring against FLC (Figure 3).

**Figure 4:**
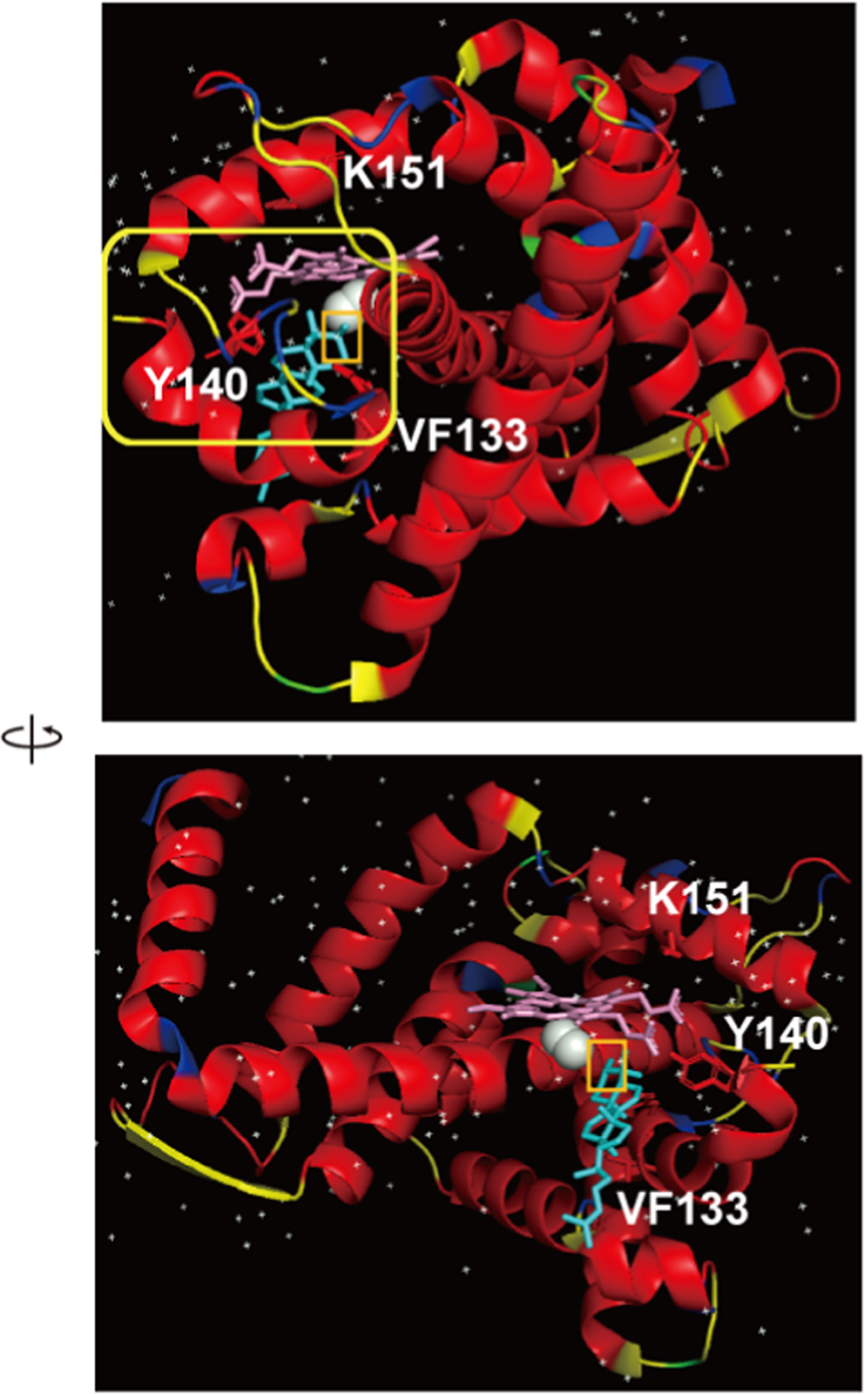
Conformational variability maps front and back (green, variable conformation) of mutation sites (yellow rectangular frame and stick structures) for *S. cerevisiae* on PDB ID 4lxj with lanosterol (cyan) and heme (pink). The oxygen molecule is represented as a sphere dimer. The hydroxyl group of lanosterol does not interact with the amino acids at the mutation sites (orange rectangular frame structures).

The conformational variability of mutation sites for clades I, III, and IV (Figure 2) decreases the population of the FLC-binding conformation. On the other hand, the hydroxyl group of lanosterol does not interact with the amino acids of ScErg11p at the mutation sites at all (orange and yellow rectangular frames in Figure 4).^18^ The type II difference spectra for the mutant enzymes (ScErg11p) are less intense than for the WT enzyme.^17^ They give 2–3-fold smaller differences in absorbance (Δ*A*_max_), with peaks shifted from 428 nm for the WT enzyme to 424–425 nm for the mutant enzymes.^17^ The conformational variability prediction result of ScErg11p is also consistent with the smaller differences in absorbance for the Y140F and Y140H mutant enzymes, and it is suggested that the populations of variable conformations, which correspond to the differences in Δ*A*_max_ between the WT enzyme and the mutant enzymes, potentially contribute to the triazole resistance.

### Echinocandin Resistance

*C. auris* FKS1 (1,3-β-d-glucan synthase) is a key enzyme involved in cell wall biosynthesis and is the enzymatic target of lipopeptide echinocandin drugs.^9^ Antifungal susceptibility testing of *C. auris* with CAS was challenging due to the fact that all FKS1 WT isolates exhibited an Eagle effect (also known as the paradoxical growth effect), which occurred at various intensities.^9^ For a global collection of *C. auris* isolates, the prevalence of resistance was 3.8%, which is on the order of that for *Candida glabrata*.^9^ All isolates were susceptible to CAS at a human therapeutic dose in a murine model of invasive candidiasis, except for those harboring the S639F mutation at the position equivalent to the well-characterized position S645 in FKS1 HS1 in *C. albicans*.^9^

The SSSCPreds concordance rates of the entire FKS1 subunit for *C. auris*, *C. albicans*, *C. glabrata*, and *S. cerevisiae* were 0.72–0.74, 0.68–0.75, 0.70–0.73, and 0.68–0.73, respectively. The value size of the concordance rates suggested that it is difficult to obtain the crystal for X-ray crystallographic analysis.

**Figure 5:**
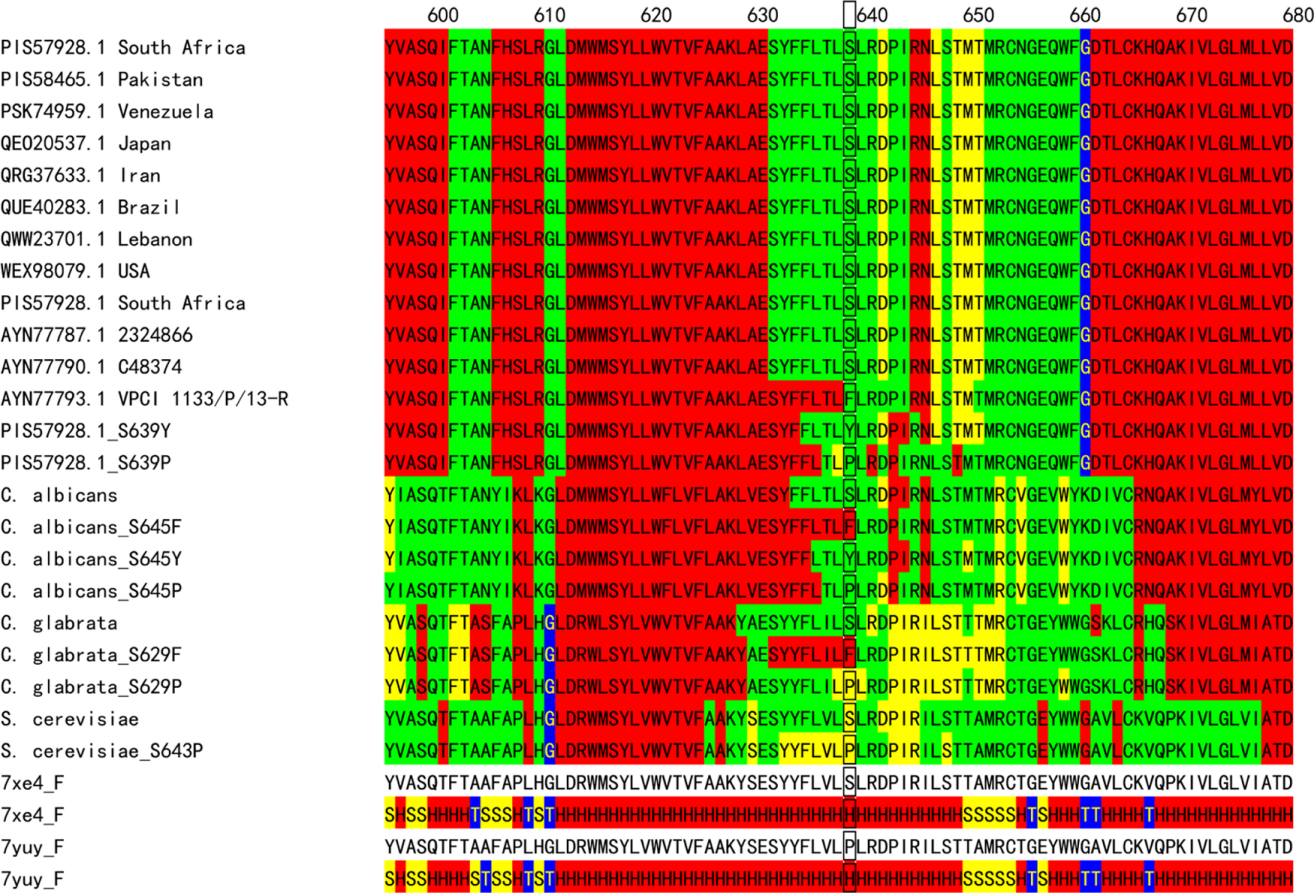
Sequence variability maps of mutation sites (FKS1) for *C. auris*, *C. albicans*, *C. glabrata*, and *S. cerevisiae* with SSSC maps of PDB structures (green, variable conformation; red, α-helix-type conformation; yellow, β-sheet-type conformation; blue, other-type conformations; black rectangles, mutation sites).

In contrast with the triazole result, the predicted variability map of HS1 for the WT of *C. auris* indicates that this region for the WT is variable (Figure 5). Most of clades I, II, III, and IV for *C. auris* do not have the mutation at S639. The mutations S639F, S639Y, and S639P make this region more rigid and stabilize the α-helix-type conformations. The mutations S645F, S645Y, and S645P for *C. albicans* and the mutation S629F for *C. glabrata* show a similar behavior, but the mutation S629P for *C. glabrata* and the mutation S643P for *S. cerevisiae* stabilize the β-sheet-type conformations.

**Figure 6:**
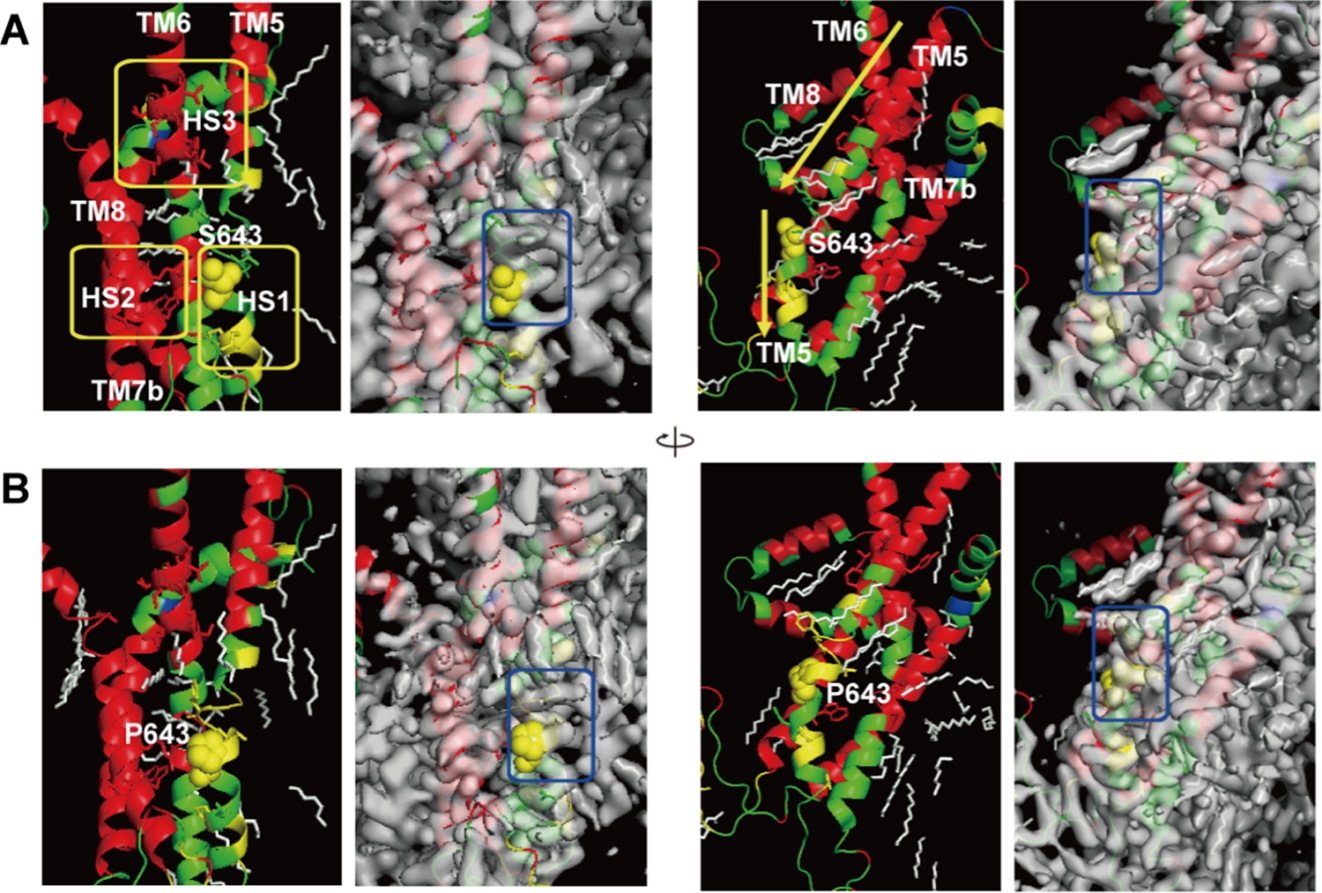
Conformational variability maps front and side (green, variable conformation) of the HS regions (S643P, spheres; other mutations, stick structure) for *S. cerevisiae* FKS1 on PDB ID 7xe4 (A) and on PDB ID 7yuy (B) with the cryo-EM maps.

Recently, Hu and co-workers reported the cryo-EM structures of β-1,3-glucan synthase FKS1 for *S. cerevisiae* and the echinocandin-resistant mutant FKS1(S643P).^19^ They noticed an elongated density along the proposed product translocation channel in FKS1(S643P), presumably corresponding to a bound product.^19^ The echinocandin-resistant mutations were clustered at a region near transmembrane (TM) domains 5–6 and TM8 of FKS1, but they could not capture the CAS-bound state of the enzyme.^19^Figure 6 shows the conformational variability maps of the HS regions for *S. cerevisiae* FKS1 and FKS1(S643P) (green, variable conformation; red, α-helix-type conformation; yellow, β-sheet-type conformation; blue, other-type conformations). FKS1 is a processive polysaccharide synthase, and its polymerized product, glucans, needs to be translocated across the membrane.^19^ For TM7b and TM8 along the proposed product translocation channel, rigid α-helix-type conformations, which correspond to those observed in the cryo-EM structures, have been predicted. On the other hand, the conformational variability prediction indicates that the S643P mutation on TM5 stabilizes the β-sheet-type conformations from the variable conformations. Although the actual cryo-EM structure near the S643P mutation site has α-helix-type conformations (Figure 5), TM5 is bent at the S643P mutation site, and the helix is largely strained (Figure 6). The lack of agreement between predicted and measured conformations suggests that the shape of the main chain with the amino acid sequence is deformable. The experimental report supports that the predicted conformational variability is convenient for this strain of the S643P mutation site. Y638 of FKS1(S643P) in the cryo-EM map of the predicted rigid motif YYFLVLP with continuous β-sheet-type conformations (Figure 5) shows a more elongated density than the variable motif for Y638 of FKS1 (blue rectangular frame in Figure 6). It may suggest a correlation between the cryo-EM map density and the predicted conformational variability.

Further, the translocation channel remains closed at the extracellular side, suggesting the existence of a regulated channel opening mechanism.^19^ The residual membrane-exposed TM9–10 and TM12 along the proposed product translocation channel at the extracellular side are short.^19^ TM5 containing HS1 contacts TM7b and TM8 along the proposed product translocation channel and the shape of the main chain with the amino acid sequence of the HS1 region are deformable as shown above. A regulatory subunit Rho1 is also necessary for 1,3-β-d-glucan synthase.^20^ Although the antifungals at concentrations >10-fold above the IC_50_ value were used, no change in the glucan length was observed.^20^ This means that the conversion efficiency to glucan was largely decreased, but the polymerization reaction was not stopped by CAS. As shown above, the CAS-bound state of the enzyme could not be captured in the cryo-EM experiments.^19^ It is suggested that CAS is involved in an uncompetitive inhibition, and the interaction of FKS1 with membrane and fungal cell wall components and Rho1 contributes to the channel opening mechanism with the conformational variability of HS1, and the impact of rigid conformations by the mutations of HS1 against the interaction reduces the binding affinity of CAS against FKS1 with Rho1 and leads to a decreased population of the CAS-bound state of FKS1 with Rho1 and to drug resistance.

All tested *C. auris* isolates from Colombia (*n* = 56), India (*n* = 40), and the Antimicrobial Resistance (AR) Isolate Bank (*n* = 10) for CAS exhibited an Eagle effect.^9^ The observed Eagle effect did not affect the in vivo drug response of *C. auris* isolates, of which the only determinant impacting the pharmacodynamic response was the FKS1 genotype.^9^ At 24 h post infection, mice challenged with FKS1 S639F mutants failed to respond to the drug, as there was no significant difference in the fungal kidney burdens between the CAS- and vehicle-treated groups.^9^ In contrast, mice infected with *C. auris* FKS1 WT isolates, irrespective of MIC and the intensity of the Eagle effect observed in vitro, had a significant kidney burden reduction upon CAS treatment relative to the kidney burden in the vehicle-treated controls.^9^ For anidulafungin (ANF) and micafungin (MCF), such a remarkable Eagle effect has not been observed.^9^ It has been suggested that some kind of interaction with the HS regions exists because HS1, HS2, and HS3 are very close (yellow rectangular frames in Figure 6).^19^ As described in the introduction, the concentrations causing reversal are represented as the multiple of MIC in many cases^10^ but not for *C. auris*.^9^ The predicted variable HS1 region for FKS1 WT isolates and the rigid one for FKS1 S639F mutants are not inconsistent with the in vivo drug response and the in vitro MIC dispersion.

On the basis of the experimental reports, a possible mechanism for the Eagle effect is proposed. The main difference of CAS relative to ANF and MCF is the length of lipid tails, and the alkyl chain of CAS is longer than those of ANF and MCF (Figure 1) so that CAS is suggested to possess high affinity against the lipid. The high concentration of CAS may allow CAS to more easily aggregate or associate in the gap among FKS1 with Rho1, the membrane, and the fungal cell wall. Further, events such as aggregation or association may reduce the population of the CAS-bound state of FKS1 with Rho1 and may lead to apparent CAS resistance. In any case, it is suggested that the conformational variability of HS1 is one of the factors for the Eagle effect with the dispersion of MIC.

## Conclusions

In conclusion, the deep neural network-based prediction of conformational variability demonstrates reasonable conformational patterns of the target sites of azole (ERG11p) and echinocandin (FKS1) drugs for *C. auris*, *C. albicans*, *C. glabrata*, and *S. cerevisiae*. The triazole resistance of *C. auris* can be rationalized by the increased populations of variable conformations accompanying the mutations of clades I, III, and IV. In contrast, the cryo-EM structures of the β-1,3-glucan synthase FKS1 and the echinocandin-resistant mutant FKS1(S643P) for *S. cerevisiae* support the interaction of FKS1 with membrane and fungal cell wall components and Rho1.^19,20^ These experimental reports correspond to the variable HS1 for the WT from the predicted variability map. The predicted conformational variability by the mutations of HS1 suggests decreased populations of the CAS-bound state of FKS1 with Rho1 and the contribution of HS1 to the channel opening mechanism.

Further, this work promotes our mechanistic understanding of the observed Eagle effect for CAS with the dispersion of the MIC. The predicted conformational variability of HS1 is not inconsistent with the in vivo drug response and the in vitro MIC dispersion. Structure-based conformational variability analysis of echinocandin-resistant mutations suggests a plausible mechanism of the Eagle effect for *C. auris* with the reduced population of the CAS-bound state of FKS1 with Rho1 accompanying events such as aggregation or association with a high concentration of CAS. In this way, this conformational variability prediction method may serve as a framework for future prediction studies of antifungal resistance and the Eagle effect, as well as for the development of new antifungals.

## Computational Methods

### SSSCview and SSSCPreds^14^

SSSC is represented as a conformation term for each amino acid peptide unit using the letters H, S, T, and D referring, respectively, to an α-helix-type conformation (H), a β-sheet-type conformation (S), a variety of other-type conformations (T), and disordered residues or the C-terminus (D), which is derived from the template patterns, characterized as conformational codes,^21,22^ such as 3a5c4a (α-helix-type conformation) and 6c4a4a (β-sheet-type conformation).^23^ The observed PDB structure files were converted to FASTA-format files containing the amino acid sequences and SSSCs of protein subunits using SSSCview.^14,24^ Python^25^ and Biopython^26^ were used to construct SSSCview. Assignment of “T” conformations was carried out using template patterns such as 2c6a4c (left-handed α-helix-type conformation).

The predicted SSSCs were gained from the FASTA-format files containing the original and mutation sequences of protein subunits using SSSCPreds, which has the benchmarks (average concordance rates) of the three systems SSSCPred200, SSSCPred100, and SSSCPred as follows: for SSSCPred200, CullPDB,^27^ 0.905 (9851 subunits) and CB513,^27^ 0.911 (361 subunits); for SSSCPred100, CullPDB, 0.896 (17,169 subunits) and CB513, 0.907 (612 subunits); and for SSSCPred, CullPDB, 0.861 (17,169 subunits) and CB513, 0.882 (612 subunits).^14^ Python^25^ and Neural Network Console 1.40^28^ were utilized to construct SSSCPreds. Software SSSCPreds and SSSCview have been deposited on the H.I. Web site (https://staff.aist.go.jp/izumi.h/SSSCPreds/index-e.html) and have been freely available.^16^

Conformational variability for each amino acid peptide unit was evaluated by using the concordance of SSSCs among SSSCPred200, SSSCPred100, and SSSCPred as the following conformations: a variable conformation (green), an α-helix-type conformation (red), a β-sheet-type conformation (yellow), and a variety of other-type conformations (blue).^16^ Sequence variability maps were obtained using the Python script with Python-docx.^29^ Conformational variability maps on the molecular model were obtained using the Python script with PyMOL.^30^

The X-ray crystallographic and cryo-EM structural data of ERG11p and FKS1 for *S. cerevisiae* were downloaded from PDBj.^31^ The amino acid sequences of ERG11p and FKS1 for *C. auris*, *C. albicans*, *C. glabrata*, and *S. cerevisiae* were obtained from UniProt^32^ or NCBI (National Center for Biotechnology Information).^33^ The converted SSSCPreds and SSSC data have been deposited on the Supporting Information because of their large size.

The cryo-EM map density (surface level 5.0 and transparency 20%) at the molecular model with the conformational variability map was depicted using PyMOL.^30^ The cryo-EM map data of FKS1 for *S. cerevisiae* were downloaded from EMDB (the Electron Microscopy Data Bank).^34^

## Supporting Information Available

SSSCPreds data of *C. auris*; *C. albicans*; *C. glabrata*; and *S. cerevisiae* with SSSC data of PDB structures (PDF)
