# Supplementary material for: Effect of Conformational Variability on the Drug Resistance of Candida auris ERG11p and FKS1
Source: ACS Omega. 2024 Apr 23;9(18):19816–23. doi: 10.1021/acsomega.3c08134 (PMC11080008; doi:10.1021/acsomega.3c08134)
Supplement: Supplementary file 1 — ao3c08134_si_001.pdf [file ao3c08134_si_001.pdf]

Supporting Information for

Effect of Conformational Variability on the Drug Resistance of *Candida auris* ERG11p and FKS1

Hiroshi Izumi,[*,†] *Laurence A. Nafie,*[‡,§] *and Rina K. Dukor*[§]

[†]National Institute of Advanced Industrial Science and Technology (AIST), AIST Tsukuba West, 16-1 Onogawa, Tsukuba, Ibaraki 305-8569, Japan
[‡]Department of Chemistry, Syracuse University, Syracuse, New York 13244-4100, United States
[§]BioTools Inc., Bee Line Hwy, Jupiter, Florida 33458, United States

e-mail: izumi.h@aist.go.jp

Table of contents

Table S1	S2-S3
Table S2	S4-S10
Table S3	S11-S18
Table S4	S19-S26
Table S5	S27-S31

Table S1. SSSCPreds data of ERG11p for *Candida auris*, *Candida albicans*, and *Saccharomyces cerevisiae* with SSSC data of PDB structures.

	1 10 20 30 40 50 60 70 80
Candida auris II	MALKDCIVDVVDRFSALPVPVKLAVLILVPIVYNLVWQFVYSLRKDRAPLVFHWVPWVGSAVVYGMQPYQFFESCREKYGDVFAF
Candida auris I	MALKDCIVDVVDRFSALPVPVKLAVLILVPIVYNLVWQFVYSLRKDRAPLVFHWVPWVGSAVVYGMQPYQFFESCREKYGDVFAF
Candida auris III	MALKDCIVDVVDRFSALPVPVKLAVLILVPIVYNLVWQFVYSLRKDRAPLVFHWVPWVGSAVVYGMQPYQFFESCREKYGDVFAF
Candida auris IV	MALKDCIVDVVDRFSALPVPVKLAVLILVPIVYNLVWQFVYSLRKDRAPLVFHWVPWVGSAVVYGMQPYQFFESCREKYGDVFAF
C. albicans Erg11p	MAIVETVIDGINYFLSLSVTQQISILLGVPFVYNLVWQYLYSLRKDRAPLVFYWIPWFGSAASYGQQPYEFFESCRQKYGDVFSF
S. cerevisiae	MSATKSIVGEALEYVNIGLSHFLALPLAQRISLIIIIPFIYNIVWQLLYSLRKDRPPLVFYWIPWVGSAVVYGMKPYEFFEECQKKYGDIFSF
4wmz_A	IVGEALEYVNIGLSHFLALPLAQRISLIIIIPFIYNIVWQLLYSLRKDRPPLVFYWIPWVGSAVVYGMKPYEFFEECQKKYGDIFSF
4wmz_A	THHHHHHHHHHHHHHHHHSSHHHHHHHHHHHHHHHHHHHHHHHHHSHHSSSSSSSHSSTHTHHHHHHHTHHHHHHHHHHHHTHSSSS
4zdz_A	SIVGEALEYVNIGLSHFLALPLAQRISLIIIIPFIYNIVWQLLYSLRKDRPPLVFYWIPWVGSAVVYGMKPYEFFEECQKKYGDIFSF
4zdz_A	TSHHHHHHHHHHHHHHHHHSSHHHHHHHHHHHHHHHHHHHHHHHHHSHHSSSSSSSHSSTHTHHHHHHHTHHHHHHHHHHHHTHSSSS
4ze3_A	VGEALEYVNIGLSHFLALPLAQRISLIIIIPFIYNIVWQLLYSLRKDRPPLVFYWIPWVGSAVVYGMKPYEFFEECQKKYGDIFSF
4ze3_A	THHHHHHHHHHHHHHHTSSHHHHHHHHHHHHHHHHHHHHHHHHHSHHSSSSSSSHSSTHTHHHHHHHTHHHHHHHHHHHHTHSSSS

	90 100 110 120 130 140 150 160 170
Candida auris II	VMLGKVMTVYLGPKGHEFVLNAKLADVSAEAAYSHLTTPVFGKGVIYDCPNSRLMEQKKFAKTALTKEAFQRYVPRIQEEVLDYF
Candida auris I	VMLGKVMTVYLGPKGHEFVLNAKLADVSAEAAYSHLTTPVFGKGVIYDCPNSRLMEQRKFAKTALTKEAFQRYVPRIQEEVLDYF
Candida auris III	VMLGKVMTVYLGPKGHEFVLNAKLADVSAEAAYSHLTTPALGKGVIYDCPNSRLMEQKKFAKTALTKEAFQRYVPRIQEEVLDYF
Candida auris IV	VMLGKVMTVYLGPKGHEFVLNAKLADVSAEAAYSHLTTPVFGKGVIFDCPNSRLMEQKKFAKTALTKEAFQRYVPRIQEEVLDYF
Candida albicans Erg11p	MLLGKIMTVYLGPKGHEFVFNAKLSDVSAEDAYKHLTTPVFGKGVIYDCPNSRLMEQKKFAKFALTTDSFKRYVPKTHGVANVMK
Candida albicans Erg11p Y132H	MLLGKIMTVYLGPKGHEFVFNAKLSDVSAEDAYKHLTTPVFGKGVIHDCPNSRLMEQKKFAKFALTTDSFKRYVPKTHGVANVMK
Saccharomyces cerevisiae	VLLGRVMTVYLGPKGHEFVFNAKLADVSAEAAYAHLTTPVFGKGVIYDCPNSRLMEQKKFVKGALTKEAFKSYVPLIAEEVYKYF
Saccharomyces cerevisiae Y140F	VLLGRVMTVYLGPKGHEFVFNAKLADVSAEAAYAHLTTPVFGKGVIFDCPNSRLMEQKKFVKGALTKEAFKSYVPLIAEEVYKYF
Saccharomyces cerevisiae Y140H	VLLGRVMTVYLGPKGHEFVFNAKLADVSAEAAYAHLTTPVFGKGVIHDCPNSRLMEQKKFVKGALTKEAFKSYVPLIAEEVYKYF
4wmz_A	VLLGRVMTVYLGPKGHEFVFNAKLADVSAEAAYAHLTTPVFGKGVIYDCPNSRLMEQKKFVKGALTKEAFKSYVPLIAEEVYKYF
4wmz_A	SSTTSSSSSSSTHHHHHHHHHSSHHHSSHHHHHHHHHHHHHTSTSTHTSSHHHHHHHHHHHHHHTSHHHHHHHHHHHHHHHHHHH
4zdz_A	VLLGRVMTVYLGPKGHEFVFNAKLADVSAEAAYAHLTTPVFGKGVIFDCPNSRLMEQKKFVKGALTKEAFKSYVPLIAEEVYKYF
4zdz_A	SSTTSSSSSSSTHHHHHHHHHSSHHHSSHHHHHHHHHHHHHTSTSTHTSSHHHHHHHHHHHHHHTSHHHHHHHHHHHHHHHHHHH
4ze3_A	VLLGRVMTVYLGPKGHEFVFNAKLADVSAEAAYAHLTTPVFGKGVIHDCPNSRLMEQKKFVKGALTKEAFKSYVPLIAEEVYKYF
4ze3_A	SSTTSSSSSSSTHHHHHHHHHSSHHHSSHHHHHHHHHHHHHTSTSTHTSSHHHHHHHHHHHHHHTSHHHHHHHHHHHHHHHHHHH

	171 180 190 200 210 220 230 240 250
Candida auris II	KACSQFKMNERNNGVANVMKTQPEMTILTASKSLMGDDMRARFDASFAKLYSDLDKGFTPINFVFPHLPLPAYWKRDAAQQKISA
Candida auris I	KACSQFKMNERNNGVANVMKTQPEMTILTASKSLMGDDMRARFDASFAKLYSDLDKGFTPINFVFPHLPLPAYWKRDAAQQKISA
Candida auris III	KACSQFKMNERNNGVANVMKTQPEMTILTASKSLMGDDMRARFDASFAKLYSDLDKGFTPINFVFPHLPLPAYWKRDAAQQKISA
Candida auris IV	KACSQFRMNERNNGVANVMKTQPEMTILTASKSLMGDDMRARFDASFAKLYSDLDKGFTPINFVFPHLPLPAYWKRDAAQQKISA
Candida albicans Erg11p	TQPEITIFTASRSLFGDEMRRIFDRSFAQLKIREYILNYFVTDESFKLKEYSDLDKGFTPINFVFPNLPLPHYWRRDAAQKKISA
Saccharomyces cerevisiae	RDSKNFRLNERTTGTIDVMVTQPEMTIFTASRSLLGKEMRAKLDTDFAYLYSDLDKGFTPINFVFPNLPLEHYRKRDHAQKAISG
4wmz_A	RDSKNFRLNERTTGTIDVMVTQPEMTIFTASRSLLGKEMRAKLDTDFAYLYSDLDKGFTPINFVFPNLPLEHYRKRDHAQKAISG
4wmz_A	HHSHHHTHHHSHSSSSSHHHHHHHHHHHHHHHHHHTHHHHHHHHSHHHHHHHHHHHHTSHHHHHTHSSHSHHHHHHHHHHHHHHH
4zdz_A	RDSKNFRLNERTTGTIDVMVTQPEMTIFTASRSLLGKEMRAKLDTDFAYLYSDLDKGFTPINFVFPNLPLEHYRKRDHAQKAISG
4zdz_A	HHSHHHTHHHSHSTSSSHHHHHHHHHHHHHHHHHHTHHHHHHHHSHHHHHHHHHHHHTSHHHHHTHSSTSHHHHHHHHHHHHHHH
4ze3_A	RDSKNFRLNERTTGTIDVMVTQPEMTIFTASRSLLGKEMRAKLDTDFAYLYSDLDKGFTPINFVFPNLPLEHYRKRDHAQKAISG
4ze3_A	HHSHHHTHHHSHSSSSSHHHHHHHHHHHHHHHHHHTHHHHHHHHSHHHHHHHHHHHHTSHHHHHTHSSHSHHHHHHHHHHHHHHH

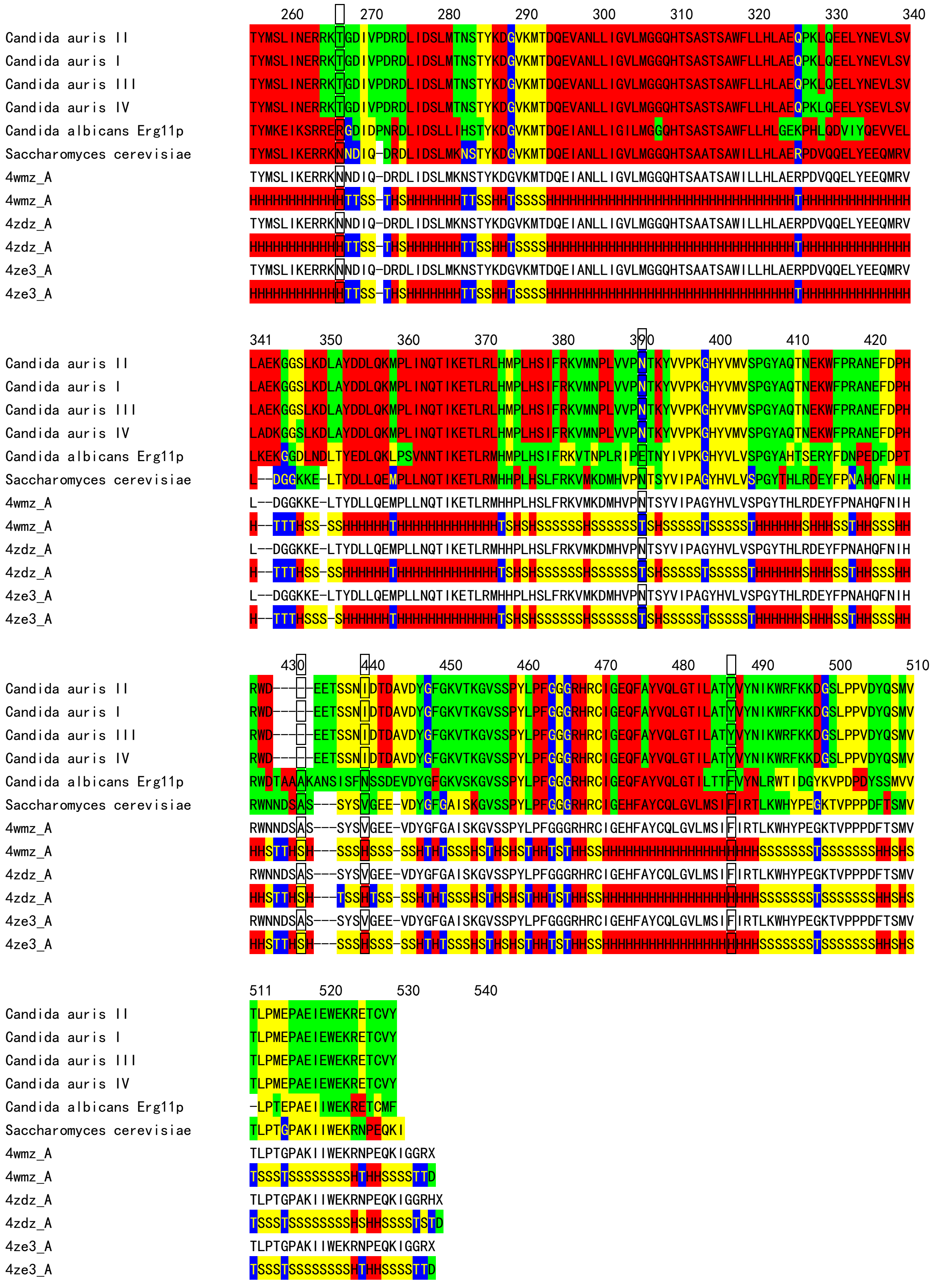

Candida auris I: UMZ08590.1, Candida auris II: XP_028891800.2, Candida auris III: PIS55918.1, Candida auris IV: PSK75255.1
Candida albicans Erg11p: AAW50593.1, Saccharomyces cerevisiae: A6ZSR0.

Table S2. SSSCPreds data of FKS1 for *Candida auris*.

	1 10 20 30 40 50 60 70 80
AYN77787.1	MSYDNNHNYYDPNQQQGGQPGGEYYQQGAYDEMGQPVNYDQAGDYYDPNQQYQQQPYDMDGYQNYGQQGAAGYSADPEAFSDFSY
AYN77790.1	MSYDNNHNYYDPNQQQGGQPGGEYYQQGAYDEMGQPVNYDQAGDYYDPNQQYQQQPYDMDGYQNYGQQGAAGYSADPEAFSDFSY
AYN77793.1	MSYDNNHNYYDPNQQQGGQPGGEYYQQGAYDEMGQPVNYDQAGDYYDPNQQYQQQPYDMDGYQNYGQQGAAGYSADPEAFSDFSY
PIS57928.1	MSYDNNHNYYDPNQQQGGQPGGEYYQQGAYDEMGQPVNYDQAGDYYDPNQQYQQQPYDMDGYQNYGQQGAAGYSADPEAFSDFSY
PIS58465.1	MSYDNNHNYYDPNQQQGGQPGGEYYQQGAYDEMGQPVNYDQAGDYYDPNQQYQQQPYDMDGYQNYGQQGAAGYSADPEAFSDFSY
PSK74959.1	MSYDNNHNYYDPNQQQGGQPGGEYYQQGAYDEMGQPVNYDQAGDYYDPNQQYQQQPYDMDGYQNYGQQGAAGYSADPEAFSDFSY
QEO20537.1	MSYDNNHNYYDPNQQQGGQPGGEYYQQGAYDEMGQPVNYDQAGDYYDPNQQYQQQPYDMDGYQNYGQQGAAGYSADPEAFSDFSY
QRG37633.1	MSYDNNHNYYDPNQQQGGQPGGEYYQQGAYDEMGQPVNYDQAGDYYDPNQQYQQQPYDMDGYQNYGQQGAAGYSADPEAFSDFSY
QUE40283.1	MSYDNNHNYYDPNQQQGGQPGGEYYQQGAYDEMGQPVNYDQAGDYYDPNQQYQQQPYDMDGYQNYGQQGAAGYSADPEAFSDFSY
QWW23701.1	MSYDNNHNYYDPNQQQGGQPGGEYYQQGAYDEMGQPVNYDQAGDYYDPNQQYQQQPYDMDGYQNYGQQGAAGYSADPEAFSDFSY
WEX98079.1	MSYDNNHNYYDPNQQQGGQPGGEYYQQGAYDEMGQPVNYDQAGDYYDPNQQYQQQPYDMDGYQNYGQQGAAGYSADPEAFSDFSY

	90 100 110 120 130 140 150 160 170
AYN77787.1	GGGHAPGTPGYDQYGAQYTPSQMSYAGARSSGASTPIYGANPNYDPSQFQLSSNLPYPAWSADPQAPIKIEHIEDIFIDLTNKFG
AYN77790.1	GGGHAPGTPGYDQYGAQYTPSQMSYAGARSSGASTPIYGANPNYDPSQFQLSSNLPYPAWSADPQAPIKIEHIEDIFIDLTNKFG
AYN77793.1	GGGHAPGTPGYDQYGAQYTPSQMSYAGARSSGASTPIYGANPNYDPSQFQLSSNLPYPAWSADPQAPIKIEHIEDIFIDLTNKFG
PIS57928.1	GGGHAPGTPGYDQYGAQYTPSQMSYAGARSSGASTPIYGANPNYDPSQFQLSSNLPYPAWSADPQAPIKIEHIEDIFIDLTNKFG
PIS58465.1	GGGHAPGTPGYDQYGAQYTPSQMSYAGARSSGASTPIYGANPNYDPSQFQLSSNLPYPAWSADPQAPIKIEHIEDIFIDLTNKFG
PSK74959.1	GGGHAPGTPGYDQYGAQYTPSQMSYAGARSSGASTPIYGANPNYDPSQFQLSSNLPYPAWSADPQAPIKIEHIEDIFIDLTNKFG
QEO20537.1	GGGHAPGTPGYDQYGAQYTPSQMSYAGARSSGASTPIYGANPNYDPSQFQLSSNLPYPAWSADPQAPIKIEHIEDIFIDLTNKFG
QRG37633.1	GGGHAPGTPGYDQYGAQYTPSQMSYAGARSSGASTPIYGANPNYDPSQFQLSSNLPYPAWSADPQAPIKIEHIEDIFIDLTNKFG
QUE40283.1	GGGHAPGTPGYDQYGAQYTPSQMSYAGARSSGASTPIYGANPNYDPSQFQLSSNLPYPAWSADPQAPIKIEHIEDIFIDLTNKFG
QWW23701.1	GGGHAPGTPGYDQYGAQYTPSQMSYAGARSSGASTPIYGANPNYDPSQFQLSSNLPYPAWSADPQAPIKIEHIEDIFIDLTNKFG
WEX98079.1	GGGHAPGTPGYDQYGAQYTPSQMSYAGARSSGASTPIYGANPNYDPSQFQLSSNLPYPAWSADPQAPIKIEHIEDIFIDLTNKFG

	171 180 190 200 210 220 230 240 250
AYN77787.1	FQRDSMRNMFDYFMTLLDSRSSRMSPAQALLSLHADYIGGDNANYKKWFFASQQDLDESIGFANMNLGKIGRKARKASKKSKKAR
AYN77790.1	FQRDSMRNMFDYFMTLLDSRSSRMSPAQALLSLHADYIGGDNANYKKWFFASQQDLDESIGFANMNLGKIGRKARKASKKSKKAR
AYN77793.1	FQRDSMRNMFDYFMTLLDSRSSRMSPAQALLSLHADYIGGDNANYKKWFFASQQDLDESIGFANMNLGKIGRKARKASKKSKKAR
PIS57928.1	FQRDSMRNMFDYFMTLLDSRSSRMSPAQALLSLHADYIGGDNANYKKWFFASQQDLDESIGFANMNLGKIGRKARKASKKSKKAR
PIS58465.1	FQRDSMRNMFDYFMTLLDSRSSRMSPAQALLSLHADYIGGDNANYKKWFFASQQDLDESIGFANMNLGKIGRKARKASKKSKKAR
PSK74959.1	FQRDSMRNMFDYFMTLLDSRSSRMSPAQALLSLHADYIGGDNANYKKWFFASQQDLDESIGFANMNLGKIGRKARKASKKSKKAR
QEO20537.1	FQRDSMRNMFDYFMTLLDSRSSRMSPAQALLSLHADYIGGDNANYKKWFFASQQDLDESIGFANMNLGKIGRKARKASKKSKKAR
QRG37633.1	FQRDSMRNMFDYFMTLLDSRSSRMSPAQALLSLHADYIGGDNANYKKWFFASQQDLDESIGFANMNLGKIGRKARKASKKSKKAR
QUE40283.1	FQRDSMRNMFDYFMTLLDSRSSRMSPAQALLSLHADYIGGDNANYKKWFFASQQDLDESIGFANMNLGKIGRKARKASKKSKKAR
QWW23701.1	FQRDSMRNMFDYFMTLLDSRSSRMSPAQALLSLHADYIGGDNANYKKWFFASQQDLDESIGFANMNLGKIGRKARKASKKSKKAR
WEX98079.1	FQRDSMRNMFDYFMTLLDSRSSRMSPAQALLSLHADYIGGDNANYKKWFFASQQDLDESIGFANMNLGKIGRKARKASKKSKKAR

	260 270 280 290 300 310 320 330 340
AYN77787.1	KAAEEHGQDIDALNNELEGDYSMEAANIRWKAKMNVLTPEERVRDIALYLLLWGEANQVRFTPELICFIFKTALDYLNSPQCQQR
AYN77790.1	KAAEEHGQDIDALNNELEGDYSMEAANIRWKAKMNVLTPEERVRDIALYLLLWGEANQVRFTPELICFIFKTALDYLNSPQCQQR
AYN77793.1	KAAEEHGQDIDALNNELEGDYSMEAANIRWKAKMNVLTPEERVRDIALYLLLWGEANQVRFTPELICFIFKTALDYLNSPQCQQR
PIS57928.1	KAAEEHGQDIDALNNELEGDYSMEAANIRWKAKMNVLTPEERVRDIALYLLLWGEANQVRFTPELICFIFKTALDYLNSPQCQQR
PIS58465.1	KAAEEHGQDIDALNNELEGDYSMEAANIRWKAKMNVLTPEERVRDIALYLLLWGEANQVRFTPELICFIFKTALDYLNSPQCQQR
PSK74959.1	KAAEEHGQDIDALNNELEGDYSMEAANIRWKAKMNVLTPEERVRDIALYLLLWGEANQVRFTPELICFIFKTALDYLNSPQCQQR
QEO20537.1	KAAEEHGQDIDALNNELEGDYSMEAANIRWKAKMNVLTPEERVRDIALYLLLWGEANQVRFTPELICFIFKTALDYLNSPQCQQR
QRG37633.1	KAAEEHGQDIDALNNELEGDYSMEAANIRWKAKMNVLTPEERVRDIALYLLLWGEANQVRFTPELICFIFKTALDYLNSPQCQQR
QUE40283.1	KAAEEHGQDIDALNNELEGDYSMEAANIRWKAKMNVLTPEERVRDIALYLLLWGEANQVRFTPELICFIFKTALDYLNSPQCQQR
QWW23701.1	KAAEEHGQDIDALNNELEGDYSMEAANIRWKAKMNVLTPEERVRDIALYLLLWGEANQVRFTPELICFIFKTALDYLNSPQCQQR
WEX98079.1	KAAEEHGQDIDALNNELEGDYSMEAANIRWKAKMNVLTPEERVRDIALYLLLWGEANQVRFTPELICFIFKTALDYLNSPQCQQR

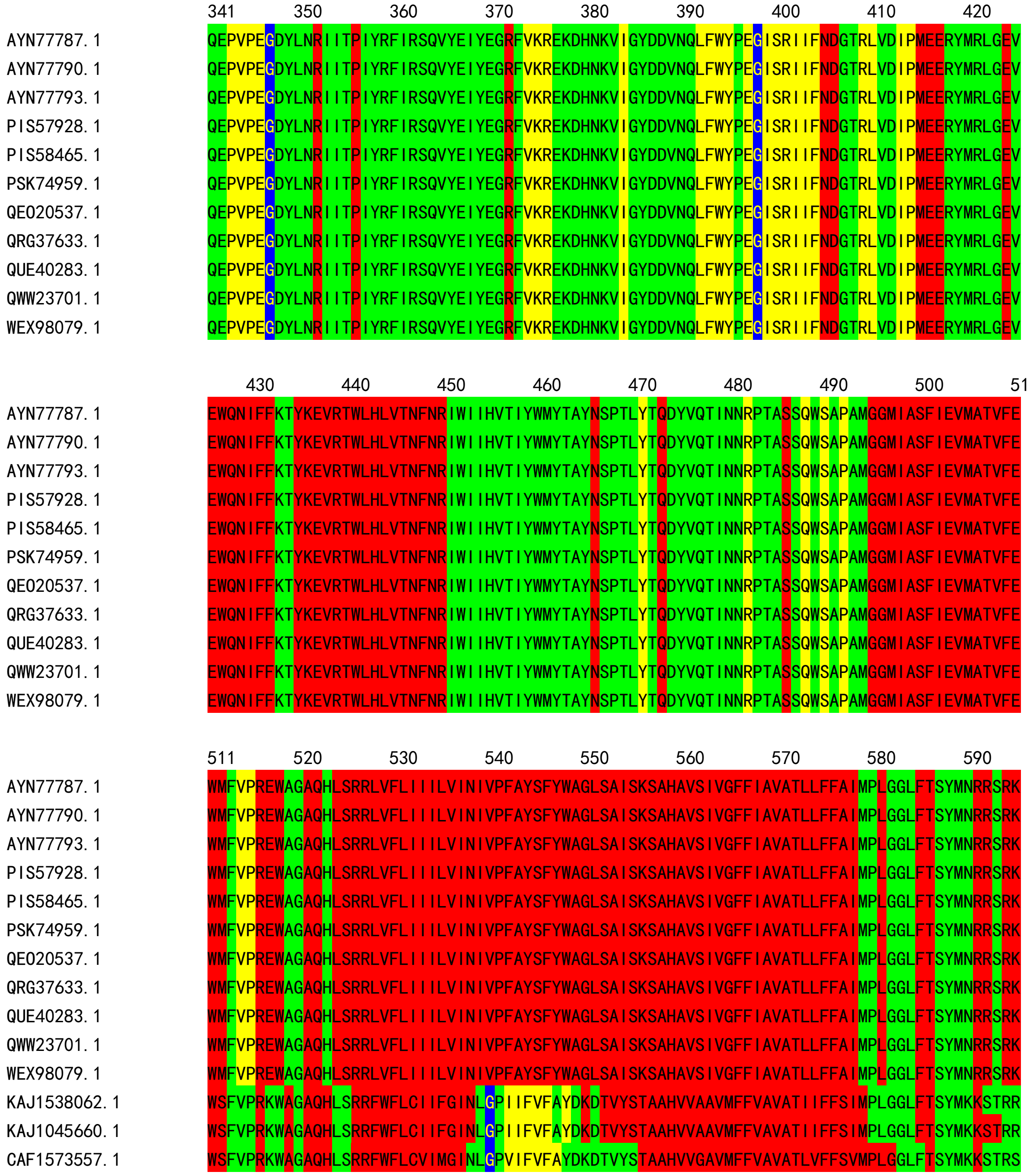
341 350 360 370 380 390 400 410 420
AYN77787.1 QEPVPEGDYLNRIITPIYRFIRSQVYEIYEGRFVKREKDHNKVIGYDDVNQLFWYPEGISRIIFNDGTRLVDIPMEERYMRLGEV
AYN77790.1 QEPVPEGDYLNRIITPIYRFIRSQVYEIYEGRFVKREKDHNKVIGYDDVNQLFWYPEGISRIIFNDGTRLVDIPMEERYMRLGEV
AYN77793.1 QEPVPEGDYLNRIITPIYRFIRSQVYEIYEGRFVKREKDHNKVIGYDDVNQLFWYPEGISRIIFNDGTRLVDIPMEERYMRLGEV
PIS57928.1 QEPVPEGDYLNRIITPIYRFIRSQVYEIYEGRFVKREKDHNKVIGYDDVNQLFWYPEGISRIIFNDGTRLVDIPMEERYMRLGEV
PIS58465.1 QEPVPEGDYLNRIITPIYRFIRSQVYEIYEGRFVKREKDHNKVIGYDDVNQLFWYPEGISRIIFNDGTRLVDIPMEERYMRLGEV
PSK74959.1 QEPVPEGDYLNRIITPIYRFIRSQVYEIYEGRFVKREKDHNKVIGYDDVNQLFWYPEGISRIIFNDGTRLVDIPMEERYMRLGEV
QE020537.1 QEPVPEGDYLNRIITPIYRFIRSQVYEIYEGRFVKREKDHNKVIGYDDVNQLFWYPEGISRIIFNDGTRLVDIPMEERYMRLGEV
QRG37633.1 QEPVPEGDYLNRIITPIYRFIRSQVYEIYEGRFVKREKDHNKVIGYDDVNQLFWYPEGISRIIFNDGTRLVDIPMEERYMRLGEV
QUE40283.1 QEPVPEGDYLNRIITPIYRFIRSQVYEIYEGRFVKREKDHNKVIGYDDVNQLFWYPEGISRIIFNDGTRLVDIPMEERYMRLGEV
QWW23701.1 QEPVPEGDYLNRIITPIYRFIRSQVYEIYEGRFVKREKDHNKVIGYDDVNQLFWYPEGISRIIFNDGTRLVDIPMEERYMRLGEV
WEX98079.1 QEPVPEGDYLNRIITPIYRFIRSQVYEIYEGRFVKREKDHNKVIGYDDVNQLFWYPEGISRIIFNDGTRLVDIPMEERYMRLGEV
430 440 450 460 470 480 490 500 510
AYN77787.1 EWQNIFFKTYKEVRTWLHLVTNFNRIWIIHVTIYWMYTAYNSPTLYTQDYVQTINNRPTASSQWSAPAMGGMIASFIEVMATVFE
AYN77790.1 EWQNIFFKTYKEVRTWLHLVTNFNRIWIIHVTIYWMYTAYNSPTLYTQDYVQTINNRPTASSQWSAPAMGGMIASFIEVMATVFE
AYN77793.1 EWQNIFFKTYKEVRTWLHLVTNFNRIWIIHVTIYWMYTAYNSPTLYTQDYVQTINNRPTASSQWSAPAMGGMIASFIEVMATVFE
PIS57928.1 EWQNIFFKTYKEVRTWLHLVTNFNRIWIIHVTIYWMYTAYNSPTLYTQDYVQTINNRPTASSQWSAPAMGGMIASFIEVMATVFE
PIS58465.1 EWQNIFFKTYKEVRTWLHLVTNFNRIWIIHVTIYWMYTAYNSPTLYTQDYVQTINNRPTASSQWSAPAMGGMIASFIEVMATVFE
PSK74959.1 EWQNIFFKTYKEVRTWLHLVTNFNRIWIIHVTIYWMYTAYNSPTLYTQDYVQTINNRPTASSQWSAPAMGGMIASFIEVMATVFE
QE020537.1 EWQNIFFKTYKEVRTWLHLVTNFNRIWIIHVTIYWMYTAYNSPTLYTQDYVQTINNRPTASSQWSAPAMGGMIASFIEVMATVFE
QRG37633.1 EWQNIFFKTYKEVRTWLHLVTNFNRIWIIHVTIYWMYTAYNSPTLYTQDYVQTINNRPTASSQWSAPAMGGMIASFIEVMATVFE
QUE40283.1 EWQNIFFKTYKEVRTWLHLVTNFNRIWIIHVTIYWMYTAYNSPTLYTQDYVQTINNRPTASSQWSAPAMGGMIASFIEVMATVFE
QWW23701.1 EWQNIFFKTYKEVRTWLHLVTNFNRIWIIHVTIYWMYTAYNSPTLYTQDYVQTINNRPTASSQWSAPAMGGMIASFIEVMATVFE
WEX98079.1 EWQNIFFKTYKEVRTWLHLVTNFNRIWIIHVTIYWMYTAYNSPTLYTQDYVQTINNRPTASSQWSAPAMGGMIASFIEVMATVFE
511 520 530 540 550 560 570 580 590
AYN77787.1 WMFVPREWAGAQHLSRRLVFLIIILVINIVPFAYSFYWAGLSAISKSAHAVSIVGFFIAVATLLFFAIMPLGGLFTSYMNRRSRK
AYN77790.1 WMFVPREWAGAQHLSRRLVFLIIILVINIVPFAYSFYWAGLSAISKSAHAVSIVGFFIAVATLLFFAIMPLGGLFTSYMNRRSRK
AYN77793.1 WMFVPREWAGAQHLSRRLVFLIIILVINIVPFAYSFYWAGLSAISKSAHAVSIVGFFIAVATLLFFAIMPLGGLFTSYMNRRSRK
PIS57928.1 WMFVPREWAGAQHLSRRLVFLIIILVINIVPFAYSFYWAGLSAISKSAHAVSIVGFFIAVATLLFFAIMPLGGLFTSYMNRRSRK
PIS58465.1 WMFVPREWAGAQHLSRRLVFLIIILVINIVPFAYSFYWAGLSAISKSAHAVSIVGFFIAVATLLFFAIMPLGGLFTSYMNRRSRK
PSK74959.1 WMFVPREWAGAQHLSRRLVFLIIILVINIVPFAYSFYWAGLSAISKSAHAVSIVGFFIAVATLLFFAIMPLGGLFTSYMNRRSRK
QE020537.1 WMFVPREWAGAQHLSRRLVFLIIILVINIVPFAYSFYWAGLSAISKSAHAVSIVGFFIAVATLLFFAIMPLGGLFTSYMNRRSRK
QRG37633.1 WMFVPREWAGAQHLSRRLVFLIIILVINIVPFAYSFYWAGLSAISKSAHAVSIVGFFIAVATLLFFAIMPLGGLFTSYMNRRSRK
QUE40283.1 WMFVPREWAGAQHLSRRLVFLIIILVINIVPFAYSFYWAGLSAISKSAHAVSIVGFFIAVATLLFFAIMPLGGLFTSYMNRRSRK
QWW23701.1 WMFVPREWAGAQHLSRRLVFLIIILVINIVPFAYSFYWAGLSAISKSAHAVSIVGFFIAVATLLFFAIMPLGGLFTSYMNRRSRK
WEX98079.1 WMFVPREWAGAQHLSRRLVFLIIILVINIVPFAYSFYWAGLSAISKSAHAVSIVGFFIAVATLLFFAIMPLGGLFTSYMNRRSRK
KAJ1538062.1 WSFVPRKWAGAQHLSRRFWFLCIIFGINLGPIIFVFAYDKDTVYSTAAHVVAAVMFFVAVATIIFFSIMPLGGLFTSYMKKSTRR
KAJ1045660.1 WSFVPRKWAGAQHLSRRFWFLCIIFGINLGPIIFVFAYDKDTVYSTAAHVVAAVMFFVAVATIIFFSIMPLGGLFTSYMKKSTRR
CAF1573557.1 WSFVPRKWAGAQHLSRRFWFLCVIMGINLGPVIFVFAYDKDTVYSTAAHVVGAVMFFVAVATLVFFSVMPLGGLFTSYMKKSTRS

```
                    600       610       620       630       640       650       660       670       680
AYN77787.1          YVASQIFTANFHSLRGLDMWMSYLLWVTVFAAKLAESYFFLTLSLRDPIRNLSTMTMRCNGEQWFGDTLCKHQAKIVLGLMLLVD
AYN77790.1          YVASQIFTANFHSLRGLDMWMSYLLWVTVFAAKLAESYFFLTLSLRDPIRNLSTMTMRCNGEQWFGDTLCKHQAKIVLGLMLLVD
AYN77793.1          YVASQIFTANFHSLRGLDMWMSYLLWVTVFAAKLAESYFFLTLFLRDPIRNLSTMTMRCNGEQWFGDTLCKHQAKIVLGLMLLVD
PIS57928.1          YVASQIFTANFHSLRGLDMWMSYLLWVTVFAAKLAESYFFLTLSLRDPIRNLSTMTMRCNGEQWFGDTLCKHQAKIVLGLMLLVD
PIS57928.1_S639Y    YVASQIFTANFHSLRGLDMWMSYLLWVTVFAAKLAESYFFLTLYLRDPIRNLSTMTMRCNGEQWFGDTLCKHQAKIVLGLMLLVD
PIS57928.1_S639P    YVASQIFTANFHSLRGLDMWMSYLLWVTVFAAKLAESYFFLTLPLRDPIRNLSTMTMRCNGEQWFGDTLCKHQAKIVLGLMLLVD
PIS58465.1          YVASQIFTANFHSLRGLDMWMSYLLWVTVFAAKLAESYFFLTLSLRDPIRNLSTMTMRCNGEQWFGDTLCKHQAKIVLGLMLLVD
PSK74959.1          YVASQIFTANFHSLRGLDMWMSYLLWVTVFAAKLAESYFFLTLSLRDPIRNLSTMTMRCNGEQWFGDTLCKHQAKIVLGLMLLVD
QE020537.1          YVASQIFTANFHSLRGLDMWMSYLLWVTVFAAKLAESYFFLTLSLRDPIRNLSTMTMRCNGEQWFGDTLCKHQAKIVLGLMLLVD
QRG37633.1          YVASQIFTANFHSLRGLDMWMSYLLWVTVFAAKLAESYFFLTLSLRDPIRNLSTMTMRCNGEQWFGDTLCKHQAKIVLGLMLLVD
QUE40283.1          YVASQIFTANFHSLRGLDMWMSYLLWVTVFAAKLAESYFFLTLSLRDPIRNLSTMTMRCNGEQWFGDTLCKHQAKIVLGLMLLVD
QWW23701.1          YVASQIFTANFHSLRGLDMWMSYLLWVTVFAAKLAESYFFLTLSLRDPIRNLSTMTMRCNGEQWFGDTLCKHQAKIVLGLMLLVD
WEX98079.1          YVASQIFTANFHSLRGLDMWMSYLLWVTVFAAKLAESYFFLTLSLRDPIRNLSTMTMRCNGEQWFGDTLCKHQAKIVLGLMLLVD
KAJ1538062.1        YVASQTFTAAFAPLHGLDRWMSYLVWVTVFAAKYSESYYFLVLSLRDPIRILSTTAMRCTGEYWWGAVLCKVQPKIVLGLVIATD
KAJ1045660.1        YIASQTFTAAFAPLHGLDRWMSYLVWVTVFAAKYSESYYFLVLSLRDPIRILSTTAMRCTGEYWWGAVLCKVQPKIVLGLVIATD
CAF1573557.1        YVASQTFTASFAPLHGLDRWMSYLVWVTVFAAKYAESYFFLILSLRDPIRILSTTSMRCTGEYWWGNKICKVQPKIVLGLMIATD
```

```
                    681      690       700       710       720       730       740       750       760
AYN77787.1          LFLFFLDTYMWYIICNCVFSIGRSFYLGISILTPWRNIFTRLPKRIYSKILATTEMEIKYKPKVLISQVWNAIVISMYREHLLAI
AYN77790.1          LFLFFLDTYMWYIICNCVFSIGRSFYLGISILTPWRNIFTRLPKRIYSKILATTEMEIKYKPKVLISQVWNAIVISMYREHLLAI
AYN77793.1          LFLFFLDTYMWYIICNCVFSIGRSFYLGISILTPWRNIFTRLPKRIYSKILATTEMEIKYKPKVLISQVWNAIVISMYREHLLAI
PIS57928.1          LFLFFLDTYMWYIICNCVFSIGRSFYLGISILTPWRNIFTRLPKRIYSKILATTEMEIKYKPKVLISQVWNAIVISMYREHLLAI
PIS58465.1          LFLFFLDTYMWYIICNCVFSIGRSFYLGISILTPWRNIFTRLPKRIYSKILATTEMEIKYKPKVLISQVWNAIVISMYREHLLAI
PSK74959.1          LFLFFLDTYMWYIICNCVFSIGRSFYLGISILTPWRNIFTRLPKRIYSKILATTEMEIKYKPKVLISQVWNAIVISMYREHLLAI
QE020537.1          LFLFFLDTYMWYIICNCVFSIGRSFYLGISILTPWRNIFTRLPKRIYSKILATTEMEIKYKPKVLISQVWNAIVISMYREHLLAI
QRG37633.1          LFLFFLDTYMWYIICNCVFSIGRSFYLGISILTPWRNIFTRLPKRIYSKILATTEMEIKYKPKVLISQVWNAIVISMYREHLLAI
QUE40283.1          LFLFFLDTYMWYIICNCVFSIGRSFYLGISILTPWRNIFTRLPKRIYSKILATTEMEIKYKPKVLISQVWNAIVISMYREHLLAI
QWW23701.1          LFLFFLDTYMWYIICNCVFSIGRSFYLGISILTPWRNIFTRLPKRIYSKILATTEMEIKYKPKVLISQVWNAIVISMYREHLLAI
WEX98079.1          LFLFFLDTYMWYIICNCVFSIGRSFYLGISILTPWRNIFTRLPKRIYSKILATTEMEIKYKPKVLISQVWNAIVISMYREHLLAI
KAJ1538062.1        FILFFLDTYLWYIIVNTIFSVGKSFYLGISILTPWRNIFTRLPKRIYSKILATTDMEIKYKPKVLISQVWNAIIISMYREHLLAI
KAJ1045660.1        FILFFLDTYLWYIIVNTIFSVGKSFYLGISILTPWRNIFTRLPKRIYSKILATTDMEIKYKPKVLISQVWNAIIISMYREHLLAI
CAF1573557.1        FILFFLDTYLWYIVVNTVFSVGKSFYLGISILTPWRNIFTRLPKRIYSKILATTDMEIKYKPKVLISQIWNAIIISMYREHLLAI
```

```
                        770       780       790       800       810       820       830       840       850
AYN77787.1          DHVQKLLYHQVPSEIEGKRTLRAPTFFVSQDDNNFETEFFPRNSEAERRISFFAQSLATPILEPLPVDNMPTFTVFTPHYSEKIL
AYN77790.1          DHVQKLLYHQVPSEIEGKRTLRAPTFFVSQDDNNFETEFFPRNSEAERRISFFAQSLATPILEPLPVDNMPTFTVFTPHYSEKIL
AYN77793.1          DHVQKLLYHQVPSEIEGKRTLRAPTFFVSQDDNNFETEFFPRNSEAERRISFFAQSLATPILEPLPVDNMPTFTVFTPHYSEKIL
PIS57928.1          DHVQKLLYHQVPSEIEGKRTLRAPTFFVSQDDNNFETEFFPRNSEAERRISFFAQSLATPILEPLPVDNMPTFTVFTPHYSEKIL
PIS58465.1          DHVQKLLYHQVPSEIEGKRTLRAPTFFVSQDDNNFETEFFPRNSEAERRISFFAQSLATPILEPLPVDNMPTFTVFTPHYSEKIL
PSK74959.1          DHVQKLLYHQVPSEIEGKRTLRAPTFFVSQDDNNFETEFFPRNSEAERRISFFAQSLATPILEPLPVDNMPTFTVFTPHYSEKIL
QE020537.1          DHVQKLLYHQVPSEIEGKRTLRAPTFFVSQDDNNFETEFFPRNSEAERRISFFAQSLATPILEPLPVDNMPTFTVFTPHYSEKIL
QRG37633.1          DHVQKLLYHQVPSEIEGKRTLRAPTFFVSQDDNNFETEFFPRNSEAERRISFFAQSLATPILEPLPVDNMPTFTVFTPHYSEKIL
QUE40283.1          DHVQKLLYHQVPSEIEGKRTLRAPTFFVSQDDNNFETEFFPRNSEAERRISFFAQSLATPILEPLPVDNMPTFTVFTPHYSEKIL
QWW23701.1          DHVQKLLYHQVPSEIEGKRTLRAPTFFVSQDDNNFETEFFPRNSEAERRISFFAQSLATPILEPLPVDNMPTFTVFTPHYSEKIL
WEX98079.1          DHVQKLLYHQVPSEIEGKRTLRAPTFFVSQDDNNFETEFFPRNSEAERRISFFAQSLATPILEPLPVDNMPTFTVFTPHYSEKIL
```

```
              851       860       870       880       890       900       910       920       930
AYN77787.1    LSLREIIREDDQFSRVTLLEYLKQLHPVEWDCFVKDTKILAEETAAYENADEEERSNEDGLKAKIDDLPFYCIGFKSAAPEYTLR
AYN77790.1    LSLREIIREDDQFSRVTLLEYLKQLHPVEWDCFVKDTKILAEETAAYENADEEERSNEDGLKAKIDDLPFYCIGFKSAAPEYTLR
AYN77793.1    LSLREIIREDDQFSRVTLLEYLKQLHPVEWDCFVKDTKILAEETAAYENADEEERSNEDGLKAKIDDLPFYCIGFKSAAPEYTLR
PIS57928.1    LSLREIIREDDQFSRVTLLEYLKQLHPVEWDCFVKDTKILAEETAAYENADEEERSNEDGLKAKIDDLPFYCIGFKSAAPEYTLR
PIS58465.1    LSLREIIREDDQFSRVTLLEYLKQLHPVEWDCFVKDTKILAEETAAYENADEEERSNEDGLKAKIDDLPFYCIGFKSAAPEYTLR
PSK74959.1    LSLREIIREDDQFSRVTLLEYLKQLHPVEWDCFVKDTKILAEETAAYENADEEERSNEDGLKAKIDDLPFYCIGFKSAAPEYTLR
QE020537.1    LSLREIIREDDQFSRVTLLEYLKQLHPVEWDCFVKDTKILAEETAAYENADEEERSNEDGLKAKIDDLPFYCIGFKSAAPEYTLR
QRG37633.1    LSLREIIREDDQFSRVTLLEYLKQLHPVEWDCFVKDTKILAEETAAYENADEEERSNEDGLKAKIDDLPFYCIGFKSAAPEYTLR
QUE40283.1    LSLREIIREDDQFSRVTLLEYLKQLHPVEWDCFVKDTKILAEETAAYENADEEERSNEDGLKAKIDDLPFYCIGFKSAAPEYTLR
QWW23701.1    LSLREIIREDDQFSRVTLLEYLKQLHPVEWDCFVKDTKILAEETAAYENADEEERSNEDGLKAKIDDLPFYCIGFKSAAPEYTLR
WEX98079.1    LSLREIIREDDQFSRVTLLEYLKQLHPVEWDCFVKDTKILAEETAAYENADEEERSNEDGLKAKIDDLPFYCIGFKSAAPEYTLR

                  940       950       960       970       980       990       1000      1010      1020
AYN77787.1    TRIWASLRSQTLYRTVSGFMNYARAIKLLYRVENPELVQYFGGDPEGLELALEKMARRKFKFVVSMQRLAKFKEDEMENAEFLLR
AYN77790.1    TRIWASLRSQTLYRTVSGFMNYARAIKLLYRVENPELVQYFGGDPEGLELALEKMARRKFKFVVSMQRLAKFKEDEMENAEFLLR
AYN77793.1    TRIWASLRSQTLYRTVSGFMNYARAIKLLYRVENPELVQYFGGDPEGLELALEKMARRKFKFVVSMQRLAKFKEDEMENAEFLLR
PIS57928.1    TRIWASLRSQTLYRTVSGFMNYARAIKLLYRVENPELVQYFGGDPEGLELALEKMARRKFKFVVSMQRLAKFKEDEMENAEFLLR
PIS58465.1    TRIWASLRSQTLYRTVSGFMNYARAIKLLYRVENPELVQYFGGDPEGLELALEKMARRKFKFVVSMQRLAKFKEDEMENAEFLLR
PSK74959.1    TRIWASLRSQTLYRTVSGFMNYARAIKLLYRVENPELVQYFGGDPEGLELALEKMARRKFKFVVSMQRLAKFKEDEMENAEFLLR
QE020537.1    TRIWASLRSQTLYRTVSGFMNYARAIKLLYRVENPELVQYFGGDPEGLELALEKMARRKFKFVVSMQRLAKFKEDEMENAEFLLR
QRG37633.1    TRIWASLRSQTLYRTVSGFMNYARAIKLLYRVENPELVQYFGGDPEGLELALEKMARRKFKFVVSMQRLAKFKEDEMENAEFLLR
QUE40283.1    TRIWASLRSQTLYRTVSGFMNYARAIKLLYRVENPELVQYFGGDPEGLELALEKMARRKFKFVVSMQRLAKFKEDEMENAEFLLR
QWW23701.1    TRIWASLRSQTLYRTVSGFMNYARAIKLLYRVENPELVQYFGGDPEGLELALEKMARRKFKFVVSMQRLAKFKEDEMENAEFLLR
WEX98079.1    TRIWASLRSQTLYRTVSGFMNYARAIKLLYRVENPELVQYFGGDPEGLELALEKMARRKFKFVVSMQRLAKFKEDEMENAEFLLR

              1021      1030      1040      1050      1060      1070      1080      1090      1100
AYN77787.1    AYPDLQIAYLDEEPPLNEDEEPRVYSALIDGHCEVLDNGRRRPKFRVQLSGNPILGDGKSDNQNHAIIFHRGEYIQLIDANQDNY
AYN77790.1    AYPDLQIAYLDEEPPLNEDEEPRVYSALIDGHCEVLDNGRRRPKFRVQLSGNPILGDGKSDNQNHAIIFHRGEYIQLIDANQDNY
AYN77793.1    AYPDLQIAYLDEEPPLNEDEEPRVYSALIDGHCEVLDNGRRRPKFRVQLSGNPILGDGKSDNQNHAIIFHRGEYIQLIDANQDNY
PIS57928.1    AYPDLQIAYLDEEPPLNEDEEPRVYSALIDGHCEVLDNGRRRPKFRVQLSGNPILGDGKSDNQNHAIIFHRGEYIQLIDANQDNY
PIS58465.1    AYPDLQIAYLDEEPPLNEDEEPRVYSALIDGHCEVLDNGRRRPKFRVQLSGNPILGDGKSDNQNHAIIFHRGEYIQLIDANQDNY
PSK74959.1    AYPDLQIAYLDEEPPLNEDEEPRVYSALIDGHCEVLDNGRRRPKFRVQLSGNPILGDGKSDNQNHAIIFHRGEYIQLIDANQDNY
QE020537.1    AYPDLQIAYLDEEPPLNEDEEPRVYSALIDGHCEVLDNGRRRPKFRVQLSGNPILGDGKSDNQNHAIIFHRGEYIQLIDANQDNY
QRG37633.1    AYPDLQIAYLDEEPPLNEDEEPRVYSALIDGHCEVLDNGRRRPKFRVQLSGNPILGDGKSDNQNHAIIFHRGEYIQLIDANQDNY
QUE40283.1    AYPDLQIAYLDEEPPLNEDEEPRVYSALIDGHCEVLDNGRRRPKFRVQLSGNPILGDGKSDNQNHAIIFHRGEYIQLIDANQDNY
QWW23701.1    AYPDLQIAYLDEEPPLNEDEEPRVYSALIDGHCEVLDNGRRRPKFRVQLSGNPILGDGKSDNQNHAIIFHRGEYIQLIDANQDNY
WEX98079.1    AYPDLQIAYLDEEPPLNEDEEPRVYSALIDGHCEVLDNGRRRPKFRVQLSGNPILGDGKSDNQNHAIIFHRGEYIQLIDANQDNY

                  1110      1120      1130      1140      1150      1160      1170      1180      1190
AYN77787.1    LEECLKIRSVLAEFEELNVEHVNPYAPGLKNNNDEKPAPVAILGAREYIFSENSGVLGDVAAGKEQTFGTLFARTLAQIGGKLHY
AYN77790.1    LEECLKIRSVLAEFEELNVEHVNPYAPGLKNNNDEKPAPVAILGAREYIFSENSGVLGDVAAGKEQTFGTLFARTLAQIGGKLHY
AYN77793.1    LEECLKIRSVLAEFEELNVEHVNPYAPGLKNNNDEKPAPVAILGAREYIFSENSGVLGDVAAGKEQTFGTLFARTLAQIGGKLHY
PIS57928.1    LEECLKIRSVLAEFEELNVEHVNPYAPGLKNNNDEKPAPVAILGAREYIFSENSGVLGDVAAGKEQTFGTLFARTLAQIGGKLHY
PIS58465.1    LEECLKIRSVLAEFEELNVEHVNPYAPGLKNNNDEKPAPVAILGAREYIFSENSGVLGDVAAGKEQTFGTLFARTLAQIGGKLHY
PSK74959.1    LEECLKIRSVLAEFEELNVEHVNPYAPGLKNNNDEKPAPVAILGAREYIFSENSGVLGDVAAGKEQTFGTLFARTLAQIGGKLHY
QE020537.1    LEECLKIRSVLAEFEELNVEHVNPYAPGLKNNNDEKPAPVAILGAREYIFSENSGVLGDVAAGKEQTFGTLFARTLAQIGGKLHY
QRG37633.1    LEECLKIRSVLAEFEELNVEHVNPYAPGLKNNNDEKPAPVAILGAREYIFSENSGVLGDVAAGKEQTFGTLFARTLAQIGGKLHY
QUE40283.1    LEECLKIRSVLAEFEELNVEHVNPYAPGLKNNNDEKPAPVAILGAREYIFSENSGVLGDVAAGKEQTFGTLFARTLAQIGGKLHY
QWW23701.1    LEECLKIRSVLAEFEELNVEHVNPYAPGLKNNNDEKPAPVAILGAREYIFSENSGVLGDVAAGKEQTFGTLFARTLAQIGGKLHY
WEX98079.1    LEECLKIRSVLAEFEELNVEHVNPYAPGLKNNNDEKPAPVAILGAREYIFSENSGVLGDVAAGKEQTFGTLFARTLAQIGGKLHY
```

```
             1191     1200      1210      1220      1230      1240      1250      1260      1270
AYN77787.1   GHPDFLNATFMLTRGGVSKAQKGLHLNEDIYAGMTAMLRGGRIKHCEYYQCGKGRDMGFGSICNFTTKIGAGMGEQMLSREYYYL
AYN77790.1   GHPDFLNATFMLTRGGVSKAQKGLHLNEDIYAGMTAMLRGGRIKHCEYYQCGKGRDMGFGSICNFTTKIGAGMGEQMLSREYYYL
AYN77793.1   GHPDFLNATFMLTRGGVSKAQKGLHLNEDIYAGMTAMLRGGRIKHCEYYQCGKGRDMGFGSICNFTTKIGAGMGEQMLSREYYYL
PIS57928.1   GHPDFLNATFMLTRGGVSKAQKGLHLNEDIYAGMTAMLRGGRIKHCEYYQCGKGRDMGFGSICNFTTKIGAGMGEQMLSREYYYL
PIS58465.1   GHPDFLNATFMLTRGGVSKAQKGLHLNEDIYAGMTAMLRGGRIKHCEYYQCGKGRDMGFGSICNFTTKIGAGMGEQMLSREYYYL
PSK74959.1   GHPDFLNATFMLTRGGVSKAQKGLHLNEDIYAGMTAMLRGGRIKHCEYYQCGKGRDMGFGSICNFTTKIGAGMGEQMLSREYYYL
QE020537.1   GHPDFLNATFMLTRGGVSKAQKGLHLNEDIYAGMTAMLRGGRIKHCEYYQCGKGRDMGFGSICNFTTKIGAGMGEQMLSREYYYL
QRG37633.1   GHPDFLNATFMLTRGGVSKAQKGLHLNEDIYAGMTAMLRGGRIKHCEYYQCGKGRDMGFGSICNFTTKIGAGMGEQMLSREYYYL
QUE40283.1   GHPDFLNATFMLTRGGVSKAQKGLHLNEDIYAGMTAMLRGGRIKHCEYYQCGKGRDMGFGSICNFTTKIGAGMGEQMLSREYYYL
QWW23701.1   GHPDFLNATFMLTRGGVSKAQKGLHLNEDIYAGMTAMLRGGRIKHCEYYQCGKGRDMGFGSICNFTTKIGAGMGEQMLSREYYYL
WEX98079.1   GHPDFLNATFMLTRGGVSKAQKGLHLNEDIYAGMTAMLRGGRIKHCEYYQCGKGRDMGFGSICNFTTKIGAGMGEQMLSREYYYL

                 1280      1290      1300      1310      1320      1330      1340      1350      1360
AYN77787.1   STQLPLDRFLSFYYGHPGFHINNLFIQLSLQTFMLVLANLNSLAHESILCDYDRNVPITDPLRPFGCYNLSPAIDWIRRYTLSIF
AYN77790.1   STQLPLDRFLSFYYGHPGFHINNLFIQLSLQTFMLVLANLNSLAHESILCDYDRNVPITDPLRPFGCYNLSPAIDWIRRYTLSIF
AYN77793.1   STQLPLDRFLSFYYGHPGFHINNLFIQLSLQTFMLVLANLNSLAHESILCDYDRNVPITDPLRPFGCYNLSPAIDWIRRYTLSIF
PIS57928.1   STQLPLDRFLSFYYGHPGFHINNLFIQLSLQTFMLVLANLNSLAHESILCDYDRNVPITDPLRPFGCYNLSPAIDWIRRYTLSIF
PIS58465.1   STQLPLDRFLSFYYGHPGFHINNLFIQLSLQTFMLVLANLNSLAHESILCDYDRNVPITDPLRPFGCYNLSPAIDWIRRYTLSIF
PSK74959.1   STQLPLDRFLSFYYGHPGFHINNLFIQLSLQTFMLVLANLNSLAHESILCDYDRNVPITDPLRPFGCYNLSPAIDWIRRYTLSIF
QE020537.1   STQLPLDRFLSFYYGHPGFHINNLFIQLSLQTFMLVLANLNSLAHESILCDYDRNVPITDPLRPFGCYNLSPAIDWIRRYTLSIF
QRG37633.1   STQLPLDRFLSFYYGHPGFHINNLFIQLSLQTFMLVLANLNSLAHESILCDYDRNVPITDPLRPFGCYNLSPAIDWIRRYTLSIF
QUE40283.1   STQLPLDRFLSFYYGHPGFHINNLFIQLSLQTFMLVLANLNSLAHESILCDYDRNVPITDPLRPFGCYNLSPAIDWIRRYTLSIF
QWW23701.1   STQLPLDRFLSFYYGHPGFHINNLFIQLSLQTFMLVLANLNSLAHESILCDYDRNVPITDPLRPFGCYNLSPAIDWIRRYTLSIF
WEX98079.1   STQLPLDRFLSFYYGHPGFHINNLFIQLSLQTFMLVLANLNSLAHESILCDYDRNVPITDPLRPFGCYNLSPAIDWIRRYTLSIF

             1361     1370      1380      1390      1400      1410      1420      1430      1440
AYN77787.1   IVFWISFIPLVVQELIERGLWKATQRFFRHFISLSPMFEVFLAQIYSNSLFTDLTVGGARYISTGRGFATSRIPFSILFSRFADS
AYN77790.1   IVFWISFIPLVVQELIERGLWKATQRFFRHFISLSPMFEVFLAQIYSNSLFTDLTVGGARYISTGRGFATSRIPFSILFSRFADS
AYN77793.1   IVFWISFIPLVVQELIERGLWKATQRFFRHFISLSPMFEVFLAQIYSNSLFTDLTVGGARYISTGRGFATSRIPFSILFSRFADS
PIS57928.1   IVFWISFIPLVVQELIERGLWKATQRFFRHFISLSPMFEVFLAQIYSNSLFTDLTVGGARYISTGRGFATSRIPFSILFSRFADS
PIS58465.1   IVFWISFIPLVVQELIERGLWKATQRFFRHFISLSPMFEVFLAQIYSNSLFTDLTVGGARYISTGRGFATSRIPFSILFSRFADS
PSK74959.1   IVFWISFIPLVVQELIERGLWKATQRFFRHFISLSPMFEVFLAQIYSNSLFTDLTVGGARYISTGRGFATSRIPFSILFSRFADS
QE020537.1   IVFWISFIPLVVQELIERGLWKATQRFFRHFISLSPMFEVFLAQIYSNSLFTDLTVGGARYISTGRGFATSRIPFSILFSRFADS
QRG37633.1   IVFWISFIPLVVQELIERGLWKATQRFFRHFISLSPMFEVFLAQIYSNSLFTDLTVGGARYISTGRGFATSRIPFSILFSRFADS
QUE40283.1   IVFWISFIPLVVQELIERGLWKATQRFFRHFISLSPMFEVFLAQIYSNSLFTDLTVGGARYISTGRGFATSRIPFSILFSRFADS
QWW23701.1   IVFWISFIPLVVQELIERGLWKATQRFFRHFISLSPMFEVFLAQIYSNSLFTDLTVGGARYISTGRGFATSRIPFSILFSRFADS
WEX98079.1   IVFWISFIPLVVQELIERGLWKATQRFFRHFISLSPMFEVFLAQIYSNSLFTDLTVGGARYISTGRGFATSRIPFSILFSRFADS

                 1450      1460      1470      1480      1490      1500      1510      1520      1530
AYN77787.1   AIYMGSRSMLILLFGSVAHWQAPLLWFWASLSALMFSPFLFNPHQFAWEDFFIDYRDFIRWLSRGNTKWHRNSWIGYIKLSRSRV
AYN77790.1   AIYMGSRSMLILLFGSVAHWQAPLLWFWASLSALMFSPFLFNPHQFAWEDFFIDYRDFIRWLSRGNTKWHRNSWIGYIKLSRSRV
AYN77793.1   AIYMGSRSMLILLFGSVAHWQAPLLWFWASLSALMFSPFLFNPHQFAWEDFFIDYRDFIRWLSRGNTKWHRNSWIGYIKLSRSRV
PIS57928.1   AIYMGSRSMLILLFGSVAHWQAPLLWFWASLSALMFSPFLFNPHQFAWEDFFIDYRDFIRWLSRGNTKWHRNSWIGYIKLSRSRV
PIS58465.1   AIYMGSRSMLILLFGSVAHWQAPLLWFWASLSALMFSPFLFNPHQFAWEDFFIDYRDFIRWLSRGNTKWHRNSWIGYIKLSRSRV
PSK74959.1   AIYMGSRSMLILLFGSVAHWQAPLLWFWASLSALMFSPFLFNPHQFAWEDFFIDYRDFIRWLSRGNTKWHRNSWIGYIKLSRSRV
QE020537.1   AIYMGSRSMLILLFGSVAHWQAPLLWFWASLSALMFSPFLFNPHQFAWEDFFIDYRDFIRWLSRGNTKWHRNSWIGYIKLSRSRV
QRG37633.1   AIYMGSRSMLILLFGSVAHWQAPLLWFWASLSALMFSPFLFNPHQFAWEDFFIDYRDFIRWLSRGNTKWHRNSWIGYIKLSRSRV
QUE40283.1   AIYMGSRSMLILLFGSVAHWQAPLLWFWASLSALMFSPFLFNPHQFAWEDFFIDYRDFIRWLSRGNTKWHRNSWIGYIKLSRSRV
QWW23701.1   AIYMGSRSMLILLFGSVAHWQAPLLWFWASLSALMFSPFLFNPHQFAWEDFFIDYRDFIRWLSRGNTKWHRNSWIGYIKLSRSRV
WEX98079.1   AIYMGSRSMLILLFGSVAHWQAPLLWFWASLSALMFSPFLFNPHQFAWEDFFIDYRDFIRWLSRGNTKWHRNSWIGYIKLSRSRV
```

```
             1531     1540       1550       1560       1570       1580       1590       1600       1610
AYN77787.1   TGFKRKLTGDISEKSAGDASRAHRSNVFMADFLPCLFYAAGLFVAYTYVNAQTGVTRWSVDGRDSTEPIKVNSVVRIVICALAPV
AYN77790.1   TGFKRKLTGDISEKSAGDASRAHRSNVFMADFLPCLFYAAGLFVAYTYVNAQTGVTRWSVDGRDSTEPIKVNSVVRIVICALAPV
AYN77793.1   TGFKRKLTGDISEKSAGDASRAHRSNVFMADFLPCLFYAAGLFVAYTYVNAQTGVTRWSVDGRDSTEPIKVNSVVRIVICALAPV
PIS57928.1   TGFKRKLTGDISEKSAGDASRAHRSNVFMADFLPCLFYAAGLFVAYTYVNAQTGVTRWSVDGRDSTEPIKVNSVVRIVICALAPV
PIS58465.1   TGFKRKLTGDISEKSAGDASRAHRSNVFMADFLPCLFYAAGLFVAYTYVNAQTGVTRWSVDGRDSTEPIKVNSVVRIVICALAPV
PSK74959.1   TGFKRKLTGDISEKSAGDASRAHRSNVFMADFLPCLFYAAGLFVAYTYVNAQTGVTRWSVDGRDSTEPIKVNSVVRIVICALAPV
QEO20537.1   TGFKRKLTGDISEKSAGDASRAHRSNVFMADFLPCLFYAAGIFVAYTYVNAQTGVTRWSVDGRDSTEPIKVNSVVRIVICALAPV
QRG37633.1   TGFKRKLTGDISEKSAGDASRAHRSNVFMADFLPCLFYAAGLFVAYTYVNAQTGVTRWSVDGRDSTEPIKVNSVVRIVICALAPV
QUE40283.1   TGFKRKLTGDISEKSAGDASRAHRSNVFMADFLPCLFYAAGLFVAYTYVNAQTGVTRWSVDGRDSTEPIKVNSVVRIVICALAPV
QWW23701.1   TGFKRKLTGDISEKSAGDASRAHRSNVFMADFLPCLFYAAGLFVAYTYVNAQTGVTRWSVDGRDSTEPIKVNSVVRIVICALAPV
WEX98079.1   TGFKRKLTGDISEKSAGDASRAHRSNVFMADFLPCLFYAAGLFVAYTYVNAQTGVTRWSVDGRDSTEPIKVNSVVRIVICALAPV

                 1620       1630       1640       1650       1660       1670       1680       1690      1700
AYN77787.1   VIDIGCLGVCLGMACCAGPMLGLCCKKTGAVIAGVAHGVAVIVHLVFFIVMWVLEGFNFARMLLGIVTMIYIQRVLFKILTLLFL
AYN77790.1   VIDIGCLGVCLGMACCAGPMLGLCCKKTGAVIAGVAHGVAVIVHLVFFIVMWVLEGFNFARMLLGIVTMIYIQRVLFKILTLLFL
AYN77793.1   VIDIGCLGVCLGMACCAGPMLGLCCKKTGAVIAGVAHGVAVIVHLVFFIVMWVLEGFNFARMLLGIVTMIYIQRVLFKILTLLFL
PIS57928.1   VIDIGCLGVCLGMACCAGPMLGLCCKKTGAVIAGVAHGVAVIVHLVFFIVMWVLEGFNFARMLLGIVTMIYIQRVLFKILTLLFL
PIS58465.1   VIDIGCLGVCLGMACCAGPMLGLCCKKTGAVIAGVAHGVAVIVHLVFFIVMWVLEGFNFARMLLGIVTMIYIQRVLFKILTLLFL
PSK74959.1   VIDIGCLGVCLGMACCAGPMLGLCCKKTGAVIAGVAHGVAVIVHLVFFIVMWVLEGFNFARMLLGIVTMIYIQRVLFKILTLLFL
QEO20537.1   VIDIGCLGVCLGMACCAGPMLGLCCKKTGAVIAGVAHGVAVIVHLVFFIVMWVLEGFNFARMLLGIVTMIYIQRVLFKILTLLFL
QRG37633.1   VIDIGCLGVCLGMACCAGPMLGLCCKKTGAVIAGVAHGVAVIVHLVFFIVMWVLEGFNFARMLLGIVTMIYIQRVLFKILTLLFL
QUE40283.1   VIDIGCLGVCLGMACCAGPMLGLCCKKTGAVIAGVAHGVAVIVHLVFFIVMWVLEGFNFARMLLGIVTMIYIQRVLFKILTLLFL
QWW23701.1   VIDIGCLGVCLGMACCAGPMLGLCCKKTGAVIAGVAHGVAVIVHLVFFIVMWVLEGFNFARMLLGIVTMIYIQRVLFKILTLLFL
WEX98079.1   VIDIGCLGVCLGMACCAGPMLGLCCKKTGAVIAGVAHGVAVIVHLVFFIVMWVLEGFNFARMLLGIVTMIYIQRVLFKILTLLFL

             1701     1710       1720       1730       1740       1750       1760       1770       1780
AYN77787.1   TREFKNDKSNTAFWTGKWYNTGMGYMAATQPAREFVAKIIEMSEFAGDFILAHLILFIQLPILCIPLIDRWHSTMLFWLKPSRLI
AYN77790.1   TREFKNDKSNTAFWTGKWYNTGMGYMAATQPAREFVAKIIEMSEFAGDFILAHLILFIQLPILCIPLIDRWHSTMLFWLKPSRLI
AYN77793.1   TREFKNDKSNTAFWTGKWYNTGMGYMAATQPAREFVAKIIEMSEFAGDFILAHLILFIQLPILCIPLIDRWHSTMLFWLKPSRLI
PIS57928.1   TREFKNDKSNTAFWTGKWYNTGMGYMAATQPAREFVAKIIEMSEFAGDFILAHLILFIQLPILCIPLIDRWHSTMLFWLKPSRLI
PIS58465.1   TREFKNDKSNTAFWTGKWYNTGMGYMAATQPAREFVAKIIEMSEFAGDFILAHLILFIQLPILCIPLIDRWHSTMLFWLKPSRLI
PSK74959.1   TREFKNDKSNTAFWTGKWYNTGMGYMAATQPAREFVAKIIEMSEFAGDFILAHLILFIQLPILCIPLIDRWHSTMLFWLKPSRLI
QEO20537.1   TREFKNDKSNTAFWTGKWYNTGMGYMAATQPAREFVAKIIEMSEFAGDFILAHLILFIQLPILCIPLIDRWHSTMLFWLKPSRLI
QRG37633.1   TREFKNDKSNTAFWTGKWYNTGMGYMAATQPAREFVAKIIEMSEFAGDFILAHLILFIQLPILCIPLIDRWHSTMLFWLKPSRLI
QUE40283.1   TREFKNDKSNTAFWTGKWYNTGMGYMAATQPAREFVAKIIEMSEFAGDFILAHLILFIQLPILCIPLIDRWHSTMLFWLKPSRLI
QWW23701.1   TREFKNDKSNTAFWTGKWYNTGMGYMAATQPAREFVAKIIEMSEFAGDFILAHLILFIQLPILCIPLIDRWHSTMLFWLKPSRLI
WEX98079.1   TREFKNDKSNTAFWTGKWYNTGMGYMAATQPAREFVAKIIEMSEFAGDFILAHLILFIQLPILCIPLIDRWHSTMLFWLKPSRLI

                 1790       1800       1810       1820       1830       1840       1850       1860      1870
AYN77787.1   RPPIYSLKQAKLRKRIVRKYCTLYFAILVLFIVIIAAPAVAGRFIADNLGANMSGTFEGLFQPRKVKNNDTGKVSSWWSTSDVKI
AYN77790.1   RPPIYSLKQAKLRKRIVRKYCTLYFAILVLFIVIIAAPAVAGRFIADNLGANMSGTFEGLFQPRKVKNNDTGKVSSWWSTSDVKI
AYN77793.1   RPPIYSLKQAKLRKRIVRKYCTLYFAILVLFIVIIAAPAVAGRFIADNLGANMSGTFEGLFQPRKVKNNDTGKVSSWWSTSDVKI
PIS57928.1   RPPIYSLKQAKLRKRIVRKYCTLYFAILVLFIVIIAAPAVAGRFIADNLGANMSGTFEGLFQPRKVKNNDTGKVSSWWSTSDVKI
PIS58465.1   RPPIYSLKQAKLRKRIVRKYCTLYFAILVLFIVIIAAPAVAGRFIADNLGANMSGTFEGLFQPRKVKNNDTGKVSSWWSTSDVKI
PSK74959.1   RPPIYSLKQAKLRKRIVRKYCTLYFAILVLFIVIIAAPAVAGRFIADNLGANMSGTFEGLFQPRKVKNNDTGKVSSWWSTSDVKI
QEO20537.1   RPPIYSLKQAKLRKRIVRKYCTLYFAILVLFIVIIAAPAVAGRFIADNLGANMSGTFEGLFQPRKVKNNDTGKVSSWWSTSDVKI
QRG37633.1   RPPIYSLKQAKLRKRIVRKYCTLYFAILVLFVVIIAAPAVAGRFIADNLGANMSGTFEGLFQPRKVKNNDTGKVSSWWSTSDVKI
QUE40283.1   RPPIYSLKQAKLRKRIVRKYCTLYFAILVLFIVIIAAPAVAGRFIADNLGANMSGTFEGLFQPRKVKNNDTGKVSSWWSTSDVKI
QWW23701.1   RPPIYSLKQAKLRKRIVRKYCTLYFAILVLFIVIIAAPAVAGRFIADNLGANMSGTFEGLFQPRKVKNNDTGKVSSWWSTSDVKI
WEX98079.1   RPPIYSLKQAKLRKRIVRKYCTLYFAILVLFIVIIAAPAVAGRFIADNLGANMSGTFEGLFQPRKVKNNDTGKVSSWWSTSDVKI
```

	1871 1880
AYN77787.1	TFWSFTPTTSNVYTTKAF
AYN77790.1	TFWSFTPTTSNVYTTKAF
AYN77793.1	TFWSFTPTTSNVYTTKAF
PIS57928.1	TFWSFTPTTSNVYTTKAF
PIS58465.1	TFWSFTPTTSNVYTTKAF
PSK74959.1	TFWSFTPTTSNVYTTKAF
QEO20537.1	TFWSFTPTTSNVYTTKAF
QRG37633.1	TFWSFTPTTSNVYTTKAF
QUE40283.1	TFWSFTPTTSNVYTTKAF
QWW23701.1	TFWSFTPTTSNVYTTKAF
WEX98079.1	TFWSFTPTTSNVYTTKAF

Table S3. SSSCPreds data of FKS1 for *Candida albicans*.

	1 10 20 30 40 50 60 70 80
ADB43261.1	MSYNDNNNHYYDPNQQGGMPPHQGGEGYYQQQYDDMGQQPHQQDYYDPNAQYQQQPYDMDGYQDQANYGGQPMNAQGYNADPEAF
BAA21535.1	MSYNDNNNHYYDPNQQGGMPPHQGGEGYYQQQYDDMGQQPHQQDYYDPNAQYQQQPYDMDGYQDQANYGGQPMNAQGYNADPEAF
CAX44487.1	MSYNDNNNHYYDPNQQGGMPPHQGGEGYYQQQYDDMGQQPHQQDYYDPNAQYQQQPYDMDGYQDQANYGGQPMNAQGYNADPEAF
KGR15377.1	MSYNDNNNHYYDPNQQGGMPPHQGGEGYYQQQYDDMGQQPHQQDYYDPNAQYQQQPYDMDGYQDQANYGGQPMNAQGYNADPEAF
KGR21277.1	MSYNDNNNHYYDPNQQGGMPPHQGGEGYYQQQYDDMGQQPHQQDYYDPNAQYQQQPYDMDGYQDQANYGGQPMNAQGYNADPEAF
KGR23302.1	MSYNDNNNHYYDPNQQGGMPPHQGGEGYYQQQYDDMGQQPHQQDYYDPNAQYQQQPYDMDGYQDQANYGGQPMNAQGYNADPEAF
RLP61548.1	MSYNDNNNHYYDPNQQGGMPPHQGGEGYYQQQYDDMGQQPHQQDYYDPNAQYQQQPYDMDGYQDQANYGGQPMNAQGYNADPEAF
RLP61548.1_D648Y	MSYNDNNNHYYDPNQQGGMPPHQGGEGYYQQQYDDMGQQPHQQDYYDPNAQYQQQPYDMDGYQDQANYGGQPMNAQGYNADPEAF
RLP61548.1_F641L	MSYNDNNNHYYDPNQQGGMPPHQGGEGYYQQQYDDMGQQPHQQDYYDPNAQYQQQPYDMDGYQDQANYGGQPMNAQGYNADPEAF
RLP61548.1_F641S	MSYNDNNNHYYDPNQQGGMPPHQGGEGYYQQQYDDMGQQPHQQDYYDPNAQYQQQPYDMDGYQDQANYGGQPMNAQGYNADPEAF
RLP61548.1_P649H	MSYNDNNNHYYDPNQQGGMPPHQGGEGYYQQQYDDMGQQPHQQDYYDPNAQYQQQPYDMDGYQDQANYGGQPMNAQGYNADPEAF
RLP61548.1_S645F	MSYNDNNNHYYDPNQQGGMPPHQGGEGYYQQQYDDMGQQPHQQDYYDPNAQYQQQPYDMDGYQDQANYGGQPMNAQGYNADPEAF
RLP61548.1_S645P	MSYNDNNNHYYDPNQQGGMPPHQGGEGYYQQQYDDMGQQPHQQDYYDPNAQYQQQPYDMDGYQDQANYGGQPMNAQGYNADPEAF
RLP61548.1_S645Y	MSYNDNNNHYYDPNQQGGMPPHQGGEGYYQQQYDDMGQQPHQQDYYDPNAQYQQQPYDMDGYQDQANYGGQPMNAQGYNADPEAF

	90 100 110 120 130 140 150 160 170
ADB43261.1	SDFSYGGQTPGTPGYBQYGTQYTPSQMSYGGDPRSSGASTPIYGGQGQGYDPTQFNMSSNLPYPAWSADPQAPIKIEHIEDIFID
BAA21535.1	SDFSYGGQTPGTPGYDQYGTQYTPSQMSYGGDPRSSGASTPIYGGQGQGYDPTQFNMSSNLPYPAWSADPQAPIKIEHIEDIFID
CAX44487.1	SDFSYGGQTPGTPGYDQYGTQYTPSQMSYGGDPRSSGASTPIYGGQGQGYDPTQFNMSSNLPYPAWSADPQAPIKIEHIEDIFID
KGR15377.1	SDFSYGGQTPGTPGYDQYGTQYTPSQMSYGGDPRSSGASTPIYGGQGQGYDPTQFNMSSNLPYPAWSADPQAPIKIEHIEDIFID
KGR21277.1	SDFSYGGQTPGTPGYDQYGTQYTPSQMSYGGDPRSSGASTPIYGGQGQGYDPTQFNMSSNLPYPAWSADPQAPIKIEHIEDIFID
KGR23302.1	SDFSYGGQTPGTPGYDQYGTQYTPSQMSYGGDPRSSGASTPIYGGQGQGYDPTQFNMSSNLPYPAWSADPQAPIKIEHIEDIFID
RLP61548.1	SDFSYGGQTPGTPGYDQYGTQYTPSQMSYGGDPRSSGASTPIYGGQGQGYDPTQFNMSSNLPYPAWSADPQAPIKIEHIEDIFID
RLP61548.1_D648Y	SDFSYGGQTPGTPGYDQYGTQYTPSQMSYGGDPRSSGASTPIYGGQGQGYDPTQFNMSSNLPYPAWSADPQAPIKIEHIEDIFID
RLP61548.1_F641L	SDFSYGGQTPGTPGYDQYGTQYTPSQMSYGGDPRSSGASTPIYGGQGQGYDPTQFNMSSNLPYPAWSADPQAPIKIEHIEDIFID
RLP61548.1_F641S	SDFSYGGQTPGTPGYDQYGTQYTPSQMSYGGDPRSSGASTPIYGGQGQGYDPTQFNMSSNLPYPAWSADPQAPIKIEHIEDIFID
RLP61548.1_P649H	SDFSYGGQTPGTPGYDQYGTQYTPSQMSYGGDPRSSGASTPIYGGQGQGYDPTQFNMSSNLPYPAWSADPQAPIKIEHIEDIFID
RLP61548.1_S645F	SDFSYGGQTPGTPGYDQYGTQYTPSQMSYGGDPRSSGASTPIYGGQGQGYDPTQFNMSSNLPYPAWSADPQAPIKIEHIEDIFID
RLP61548.1_S645P	SDFSYGGQTPGTPGYDQYGTQYTPSQMSYGGDPRSSGASTPIYGGQGQGYDPTQFNMSSNLPYPAWSADPQAPIKIEHIEDIFID
RLP61548.1_S645Y	SDFSYGGQTPGTPGYDQYGTQYTPSQMSYGGDPRSSGASTPIYGGQGQGYDPTQFNMSSNLPYPAWSADPQAPIKIEHIEDIFID

	171 180 190 200 210 220 230 240 250
ADB43261.1	LTNKFGFQRDSMRNMFDYFMTLLDSRSSRMSPAQALLSLHADYIGGDNANYRKWYFSSQQDLDDSLGFANMTLGKIGRKARKASK
BAA21535.1	LTNKFGFQRDSMRNMFDYFMTLLDSRSSRMSPAQALLSLHADYIGGDNANYRKWYFSSQQDLDDSLGFANMTLGKIGRKARKASK
CAX44487.1	LANKFGFQRDSMRNMFDYFMTLLDSRSSRMSPAQALLSLHADYIGGDNANYRKWFFSSQQDLDDSLGFANMTLGKIGRKARKASK
KGR15377.1	LTNKFGFQRDSMRNMFDYFMTLLDSRSSRMSPAQALLSLHADYIGGDNANYRKWYFSSQQDLDDSLGFANMTLGKIGRKARKASK
KGR21277.1	LTNKFGFQRDSMRNMFDYFMTLLDSRSSRMSPAQALLSLHADYIGGDNANYRKWYFSSQQDLDDSLGFANMTLGKIGRKARKASK
KGR23302.1	LTNKFGFQRDSMRNMFDYFMTLLDSRSSRMSPAQALLSLHADYIGGDNANYRKWYFSSQQDLDDSLGFANMTLGKIGRKARKASK
RLP61548.1	LTNKFGFQRDSMRNMFDYFMTLLDSRSSRMSPAQALLSLHADYIGGDNANYRKWYFSSQQDLDDSLGFANMTLGKIGRKARKASK
RLP61548.1_D648Y	LTNKFGFQRDSMRNMFDYFMTLLDSRSSRMSPAQALLSLHADYIGGDNANYRKWYFSSQQDLDDSLGFANMTLGKIGRKARKASK
RLP61548.1_F641L	LTNKFGFQRDSMRNMFDYFMTLLDSRSSRMSPAQALLSLHADYIGGDNANYRKWYFSSQQDLDDSLGFANMTLGKIGRKARKASK
RLP61548.1_F641S	LTNKFGFQRDSMRNMFDYFMTLLDSRSSRMSPAQALLSLHADYIGGDNANYRKWYFSSQQDLDDSLGFANMTLGKIGRKARKASK
RLP61548.1_P649H	LTNKFGFQRDSMRNMFDYFMTLLDSRSSRMSPAQALLSLHADYIGGDNANYRKWYFSSQQDLDDSLGFANMTLGKIGRKARKASK
RLP61548.1_S645F	LTNKFGFQRDSMRNMFDYFMTLLDSRSSRMSPAQALLSLHADYIGGDNANYRKWYFSSQQDLDDSLGFANMTLGKIGRKARKASK
RLP61548.1_S645P	LTNKFGFQRDSMRNMFDYFMTLLDSRSSRMSPAQALLSLHADYIGGDNANYRKWYFSSQQDLDDSLGFANMTLGKIGRKARKASK
RLP61548.1_S645Y	LTNKFGFQRDSMRNMFDYFMTLLDSRSSRMSPAQALLSLHADYIGGDNANYRKWYFSSQQDLDDSLGFANMTLGKIGRKARKASK

```
                   260       270       280       290       300       310       320       330       340
ADB43261.1         KSKKARKAAEEHGQDVDALANELEGDYSLEAAEIRWKAKMNSLTPEERVRDLALYLLIWGEANQVRFTPECLCYIYKSATDYLNS
BAA21535.1         KSKKARKAAEEHGQDVDALANELEGDYSLEAAEIRWKAKMNSLTPEERVRDLALYLLIWGEANQVRFTPECLCYIYKSATDYLNS
CAX44487.1         KSKKARKAAEEHGQDVDALANELEGDYSLEAAEIRWKAKMNSLTPEERVRDLALYLLIWGEANQVRFTPECLCYIYKAATDYLNS
KGR15377.1         KSKKARKAAEEHGQDVDALANELEGDYSLEAAEIRWKAKMNSLTPEERVRDLALYLLIWGEANQVRFTPECLCYIYKSATDYLNS
KGR21277.1         KSKKARKAAEEHGQDVDALANELEGDYSLEAAEIRWKAKMNSLTPEERVRDLALYLLIWGEANQVRFTPECLCYIYKSATDYLNS
KGR23302.1         KSKKARKAAEEHGQDVDALANELEGDYSLEAAEIRWKAKMNSLTPEERVRDLALYLLIWGEANQVRFTPECLCYIYKSATDYLNS
RLP61548.1         KSKKARKAAEEHGQDVDALANELEGDYSLEAAEIRWKAKMNSLTPEERVRDLALYLLIWGEANQVRFTPECLCYIYKSATDYLNS
RLP61548.1_D648Y   KSKKARKAAEEHGQDVDALANELEGDYSLEAAEIRWKAKMNSLTPEERVRDLALYLLIWGEANQVRFTPECLCYIYKSATDYLNS
RLP61548.1_F641L   KSKKARKAAEEHGQDVDALANELEGDYSLEAAEIRWKAKMNSLTPEERVRDLALYLLIWGEANQVRFTPECLCYIYKSATDYLNS
RLP61548.1_F641S   KSKKARKAAEEHGQDVDALANELEGDYSLEAAEIRWKAKMNSLTPEERVRDLALYLLIWGEANQVRFTPECLCYIYKSATDYLNS
RLP61548.1_P649H   KSKKARKAAEEHGQDVDALANELEGDYSLEAAEIRWKAKMNSLTPEERVRDLALYLLIWGEANQVRFTPECLCYIYKSATDYLNS
RLP61548.1_S645F   KSKKARKAAEEHGQDVDALANELEGDYSLEAAEIRWKAKMNSLTPEERVRDLALYLLIWGEANQVRFTPECLCYIYKSATDYLNS
RLP61548.1_S645P   KSKKARKAAEEHGQDVDALANELEGDYSLEAAEIRWKAKMNSLTPEERVRDLALYLLIWGEANQVRFTPECLCYIYKSATDYLNS
RLP61548.1_S645Y   KSKKARKAAEEHGQDVDALANELEGDYSLEAAEIRWKAKMNSLTPEERVRDLALYLLIWGEANQVRFTPECLCYIYKSATDYLNS
```

```
                   341      350       360       370       380       390       400       410       420
ADB43261.1         PLCQQRQEPVPEGDYLNRVITPLYRFIRSQVYEIYDGRFVKREKDHNKVIGYDDVNQLFWYPEGISRIIFEDGTRLVDIPQEERF
BAA21535.1         PLCQQRQEPVPEGDYLNRVITPLYRFIRSQVYEIYDGRFVKREKDHNKVIGYDDVNQLFWYPEGISRIIFEDGTRLVDIPQEERF
CAX44487.1         PLCQQRQEPVLEGDYLNRVITPLYRFIRSQVYEIYDGRFVKREKDHNKVIGYDDVNQLFWYPEGISRIIFEDGTRLVDIPQEERF
KGR15377.1         PLCQQRQEPVPEGDYLNRVITPLYRFIRSQVYEIYDGRFVKREKDHNKVIGYDDVNQLFWYPEGISRIIFEDGTRLVDIPQEERF
KGR21277.1         PLCQQRQEPVPEGDYLNRVITPLYRFIRSQVYEIYDGRFVKREKDHNKVIGYDDVNQLFWYPEGISRIIFEDGTRLVDIPQEERF
KGR23302.1         PLCQQRQEPVPEGDYLNRVITPLYRFIRSQVYEIYDGRFVKREKDHNKVIGYDDVNQLFWYPEGISRIIFEDGTRLVDIPQEERF
RLP61548.1         PLCQQRQEPVPEGDYLNRVITPLYRFIRSQVYEIYDGRFVKREKDHNKVIGYDDVNQLFWYPEGISRIIFEDGTRLVDIPQEERF
RLP61548.1_D648Y   PLCQQRQEPVPEGDYLNRVITPLYRFIRSQVYEIYDGRFVKREKDHNKVIGYDDVNQLFWYPEGISRIIFEDGTRLVDIPQEERF
RLP61548.1_F641L   PLCQQRQEPVPEGDYLNRVITPLYRFIRSQVYEIYDGRFVKREKDHNKVIGYDDVNQLFWYPEGISRIIFEDGTRLVDIPQEERF
RLP61548.1_F641S   PLCQQRQEPVPEGDYLNRVITPLYRFIRSQVYEIYDGRFVKREKDHNKVIGYDDVNQLFWYPEGISRIIFEDGTRLVDIPQEERF
RLP61548.1_P649H   PLCQQRQEPVPEGDYLNRVITPLYRFIRSQVYEIYDGRFVKREKDHNKVIGYDDVNQLFWYPEGISRIIFEDGTRLVDIPQEERF
RLP61548.1_S645F   PLCQQRQEPVPEGDYLNRVITPLYRFIRSQVYEIYDGRFVKREKDHNKVIGYDDVNQLFWYPEGISRIIFEDGTRLVDIPQEERF
RLP61548.1_S645P   PLCQQRQEPVPEGDYLNRVITPLYRFIRSQVYEIYDGRFVKREKDHNKVIGYDDVNQLFWYPEGISRIIFEDGTRLVDIPQEERF
RLP61548.1_S645Y   PLCQQRQEPVPEGDYLNRVITPLYRFIRSQVYEIYDGRFVKREKDHNKVIGYDDVNQLFWYPEGISRIIFEDGTRLVDIPQEERF
```

```
                   430       440       450       460       470       480       490       500       510
ADB43261.1         LKLGEVEWKNVFFKTYKEIRTWLHFVTNFNRIWIIHGTIYWMYTAYNSPTLYTKHYVQTINQQPLASSRWAACAIGGVLASFIQI
BAA21535.1         LKLGEVEWKNVFFKTYKEIRTWLHFVTNFNRIWIIHGTIYWMYTAYNSPTLYTKHYVQTINQQPLASSRWAACAIGGVLASFIQI
CAX44487.1         LKLGEVEWKNVFFKTYKEIRTWLHFVTNFNRIWIIHGTIYWMYTAYNSPTLYTKHYVQTMNQQPLASSRWAACAIGGVLAAFIQI
KGR15377.1         LKLGEVEWKNVFFKTYKEIRTWLHFVTNFNRIWIIHGTIYWMYTAYNSPTLYTKHYVQTINQQPLASSRWAACAIGGVLASFIQI
KGR21277.1         LKLGEVEWKNVFFKTYKEIRTWLHFVTNFNRIWIIHGTIYWMYTAYNSPTLYTKHYVQTINQQPLASSRWAACAIGGVLASFIQI
KGR23302.1         LKLGEVEWKNVFFKTYKEIRTWLHFVTNFNRIWIIHGTIYWMYTAYNSPTLYTKHYVQTINQQPLASSRWAACAIGGVLASFIQI
RLP61548.1         LKLGEVEWKNVFFKTYKEIRTWLHFVTNFNRIWIIHGTIYWMYTAYNSPTLYTKHYVQTINQQPLASSRWAACAIGGVLASFIQI
RLP61548.1_D648Y   LKLGEVEWKNVFFKTYKEIRTWLHFVTNFNRIWIIHGTIYWMYTAYNSPTLYTKHYVQTINQQPLASSRWAACAIGGVLASFIQI
RLP61548.1_F641L   LKLGEVEWKNVFFKTYKEIRTWLHFVTNFNRIWIIHGTIYWMYTAYNSPTLYTKHYVQTINQQPLASSRWAACAIGGVLASFIQI
RLP61548.1_F641S   LKLGEVEWKNVFFKTYKEIRTWLHFVTNFNRIWIIHGTIYWMYTAYNSPTLYTKHYVQTINQQPLASSRWAACAIGGVLASFIQI
RLP61548.1_P649H   LKLGEVEWKNVFFKTYKEIRTWLHFVTNFNRIWIIHGTIYWMYTAYNSPTLYTKHYVQTINQQPLASSRWAACAIGGVLASFIQI
RLP61548.1_S645F   LKLGEVEWKNVFFKTYKEIRTWLHFVTNFNRIWIIHGTIYWMYTAYNSPTLYTKHYVQTINQQPLASSRWAACAIGGVLASFIQI
RLP61548.1_S645P   LKLGEVEWKNVFFKTYKEIRTWLHFVTNFNRIWIIHGTIYWMYTAYNSPTLYTKHYVQTINQQPLASSRWAACAIGGVLASFIQI
RLP61548.1_S645Y   LKLGEVEWKNVFFKTYKEIRTWLHFVTNFNRIWIIHGTIYWMYTAYNSPTLYTKHYVQTINQQPLASSRWAACAIGGVLASFIQI
```

```
                     511      520        530       540        550       560       570       580       590
ADB43261.1           LATLFEWIFVPREWAGAQHLSRRMLFLVLIFLLNLVPPVYTFQITKLVIYSKSAYAVSIVGFFIAVATLVFFAVMPLGGLFTSYM
BAA21535.1           LATLFEWIFVPREWAGAQHLSRRMLFLVLIFLLNLVPPVYTFQITKLVIYSKSAYAVSIVGFFIAVATLVFFAVMPLGGLFTSYM
CAX44487.1           LATLFEWIFVPREWAGAQHLSRRMVFLVLIFFLNLVPPVYTFQITKLVIYSKSAYAVSVVGFFIAVATLVFFAVMPLGGLFTSYM
KGR15377.1           LATLFEWIFVPREWAGAQHLSRRMLFLVLIFLLNLVPPVYTFQITKLVIYSKSAYAVSIVGFFIAVATLVFFAVMPLGGLFTSYM
KGR21277.1           LATLFEWIFVPREWAGAQHLSRRMLFLVLIFLLNLVPPVYTFQITKLVIYSKSAYAVSIVGFFIAVATLVFFAVMPLGGLFTSYM
KGR23302.1           LATLFEWIFVPREWAGAQHLSRRMLFLVLIFLLNLVPPVYTFQITKLVIYSKSAYAVSIVGFFIAVATLVFFAVMPLGGLFTSYM
RLP61548.1           LATLFEWIFVPREWAGAQHLSRRMLFLVLIFLLNLVPPVYTFQITKLVIYSKSAYAVSIVGFFIAVATLVFFAVMPLGGLFTSYM
RLP61548.1_D648Y     LATLFEWIFVPREWAGAQHLSRRMLFLVLIFLLNLVPPVYTFQITKLVIYSKSAYAVSIVGFFIAVATLVFFAVMPLGGLFTSYM
RLP61548.1_F641L     LATLFEWIFVPREWAGAQHLSRRMLFLVLIFLLNLVPPVYTFQITKLVIYSKSAYAVSIVGFFIAVATLVFFAVMPLGGLFTSYM
RLP61548.1_F641S     LATLFEWIFVPREWAGAQHLSRRMLFLVLIFLLNLVPPVYTFQITKLVIYSKSAYAVSIVGFFIAVATLVFFAVMPLGGLFTSYM
RLP61548.1_P649H     LATLFEWIFVPREWAGAQHLSRRMLFLVLIFLLNLVPPVYTFQITKLVIYSKSAYAVSIVGFFIAVATLVFFAVMPLGGLFTSYM
RLP61548.1_S645F     LATLFEWIFVPREWAGAQHLSRRMLFLVLIFLLNLVPPVYTFQITKLVIYSKSAYAVSIVGFFIAVATLVFFAVMPLGGLFTSYM
RLP61548.1_S645P     LATLFEWIFVPREWAGAQHLSRRMLFLVLIFLLNLVPPVYTFQITKLVIYSKSAYAVSIVGFFIAVATLVFFAVMPLGGLFTSYM
RLP61548.1_S645Y     LATLFEWIFVPREWAGAQHLSRRMLFLVLIFLLNLVPPVYTFQITKLVIYSKSAYAVSIVGFFIAVATLVFFAVMPLGGLFTSYM

                         600        610       620        630       640        650       660        670       680
ADB43261.1           NKRSRRYIASQTFTANYIKLKGLDMWMSYLLWFLVFLAKLVESYFFLTLSLRDPIRNLSTMTMRCVGEVWYKDIVCRNQAKIVLG
BAA21535.1           NKRSRRYIASQTFTANYIKLKGLDMWMSYLLWFLVFLAKLVESYFFSTLSLRDPIRNLSTMTMRCVGEVWYKDIVCRNQAKIVLG
CAX44487.1           NKRSRRYIASQTFTANYIKLKGLDMWMSYLLWFLVFLAKLVESYFFLTLSLRDPIRNLSTMSMRCVGEVWYKDIVCKNQAKIVLG
KGR15377.1           NKRSRRYIASQTFTANYIKLKGLDMWMSYLLWFLVFLAKLVESYFFLTLSLRDPIRNLSTMTMRCVGEVWYKDIVCRNQAKIVLG
KGR21277.1           NKRSRRYIASQTFTANYIKLKGLDMWMSYLLWFLVFLAKLVESYFFLTLSLRDPIRNLSTMTMRCVGEVWYKDIVCRNQAKIVLG
KGR23302.1           NKRSRRYIASQTFTANYIKLKGLDMWMSYLLWFLVFLAKLVESYFFLTLSLRDPIRNLSTMTMRCVGEVWYKDIVCRNQAKIVLG
RLP61548.1           NKRSRRYIASQTFTANYIKLKGLDMWMSYLLWFLVFLAKLVESYFFLTLSLRDPIRNLSTMTMRCVGEVWYKDIVCRNQAKIVLG
RLP61548.1_F641L     NKRSRRYIASQTFTANYIKLKGLDMWMSYLLWFLVFLAKLVESYFLLTLSLRDPIRNLSTMTMRCVGEVWYKDIVCRNQAKIVLG
RLP61548.1_F641S     NKRSRRYIASQTFTANYIKLKGLDMWMSYLLWFLVFLAKLVESYFSLTLSLRDPIRNLSTMTMRCVGEVWYKDIVCRNQAKIVLG
RLP61548.1_S645F     NKRSRRYIASQTFTANYIKLKGLDMWMSYLLWFLVFLAKLVESYFFLTLFLRDPIRNLSTMTMRCVGEVWYKDIVCRNQAKIVLG
RLP61548.1_S645P     NKRSRRYIASQTFTANYIKLKGLDMWMSYLLWFLVFLAKLVESYFFLTLPLRDPIRNLSTMTMRCVGEVWYKDIVCRNQAKIVLG
RLP61548.1_S645Y     NKRSRRYIASQTFTANYIKLKGLDMWMSYLLWFLVFLAKLVESYFFLTLYLRDPIRNLSTMTMRCVGEVWYKDIVCRNQAKIVLG
RLP61548.1_D648Y     NKRSRRYIASQTFTANYIKLKGLDMWMSYLLWFLVFLAKLVESYFFLTLSLRYPIRNLSTMTMRCVGEVWYKDIVCRNQAKIVLG
RLP61548.1_P649H     NKRSRRYIASQTFTANYIKLKGLDMWMSYLLWFLVFLAKLVESYFFLTLSLRDHIRNLSTMTMRCVGEVWYKDIVCRNQAKIVLG

                     681      690        700       710        720       730       740        750       760
ADB43261.1           LMYLVDLLLFFLDTYMWYIICNCIFSIGRSFYLGISILTPWRNIFTRLPKRIYSKILATTEMEIKYKPKVLISQIWNAIVISMYR
BAA21535.1           LMYLVDLLLFFLDTYMWYIICNCIFSIGRSFYLGISILTPWRNIFTRLPKRIYSKILATTEMEIKYKPKVLISQIWNAIVISMYR
CAX44487.1           LMYLVDLLLFFLDTYMWYIICNCIFSIGRSFYLGISILTPWRNIFTRLPKRIYSKILATTEMEIKYKPKVLISQIWNAIVISMYR
KGR15377.1           LMYLVDLLLFFLDTYMWYIICNCIFSIGRSFYLGISILTPWRNIFTRLPKRIYSKILATTEMEIKYKPKVLISQIWNAIVISMYR
KGR21277.1           LMYLVDLLLFFLDTYMWYIICNCIFSIGRSFYLGISILTPWRNIFTRLPKRIYSKILATTEMEIKYKPKVLISQIWNAIVISMYR
KGR23302.1           LMYLVDLLLFFLDTYMWYIICNCIFSIGRSFYLGISILTPWRNIFTRLPKRIYSKILATTEMEIKYKPKVLISQIWNAIVISMYR
RLP61548.1           LMYLVDLLLFFLDTYMWYIICNCIFSIGRSFYLGISILTPWRNIFTRLPKRIYSKILATTEMEIKYKPKVLISQIWNAIVISMYR
RLP61548.1_D648Y     LMYLVDLLLFFLDTYMWYIICNCIFSIGRSFYLGISILTPWRNIFTRLPKRIYSKILATTEMEIKYKPKVLISQIWNAIVISMYR
RLP61548.1_F641L     LMYLVDLLLFFLDTYMWYIICNCIFSIGRSFYLGISILTPWRNIFTRLPKRIYSKILATTEMEIKYKPKVLISQIWNAIVISMYR
RLP61548.1_F641S     LMYLVDLLLFFLDTYMWYIICNCIFSIGRSFYLGISILTPWRNIFTRLPKRIYSKILATTEMEIKYKPKVLISQIWNAIVISMYR
RLP61548.1_P649H     LMYLVDLLLFFLDTYMWYIICNCIFSIGRSFYLGISILTPWRNIFTRLPKRIYSKILATTEMEIKYKPKVLISQIWNAIVISMYR
RLP61548.1_S645F     LMYLVDLLLFFLDTYMWYIICNCIFSIGRSFYLGISILTPWRNIFTRLPKRIYSKILATTEMEIKYKPKVLISQIWNAIVISMYR
RLP61548.1_S645P     LMYLVDLLLFFLDTYMWYIICNCIFSIGRSFYLGISILTPWRNIFTRLPKRIYSKILATTEMEIKYKPKVLISQIWNAIVISMYR
RLP61548.1_S645Y     LMYLVDLLLFFLDTYMWYIICNCIFSIGRSFYLGISILTPWRNIFTRLPKRIYSKILATTEMEIKYKPKVLISQIWNAIVISMYR
```

```
                      770       780       790       800       810       820       830       840       850
ADB43261.1            EHLLAIDHVQKLLYHQVPSEIEGKRTLRAPTFFVSQDDNNFETEFFPRNSEAERRISFFAQSLATPMPEPLPVDNMPTFTVFTPH
BAA21535.1            EHLLAIDHVQKLLYHQVPSEIEGKRTLRAPTFFVSQDDNNFETEFFPRNSEAERRISFFAQSLATPMPEPLPVDNMPTFTVFTPH
CAX44487.1            EHLLAIDHVQKLLYHQVPSEIEGKRTLRAPTFFVSQDDNNFETEFFPRNSEAERRISFFAQSLATPMPEPLPVDNMPTFTVFTPH
KGR15377.1            EHLLAIDHVQKLLYHQVPSEIEGKRTLRAPTFFVSQDDNNFETEFFPRNSEAERRISFFAQSLATPMPEPLPVDNMPTFTVFTPH
KGR21277.1            EHLLAIDHVQKLLYHQVPSEIEGKRTLRAPTFFVSQDDNNFETEFFPRNSEAERRISFFAQSLATPMPEPLPVDNMPTFTVFTPH
KGR23302.1            EHLLAIDHVQKLLYHQVPSEIEGKRTLRAPTFFVSQDDNNFETEFFPRNSEAERRISFFAQSLATPMPEPLPVDNMPTFTVFTPH
RLP61548.1            EHLLAIDHVQKLLYHQVPSEIEGKRTLRAPTFFVSQDDNNFETEFFPRNSEAERRISFFAQSLATPMPEPLPVDNMPTFTVFTPH
RLP61548.1_D648Y      EHLLAIDHVQKLLYHQVPSEIEGKRTLRAPTFFVSQDDNNFETEFFPRNSEAERRISFFAQSLATPMPEPLPVDNMPTFTVFTPH
RLP61548.1_F641L      EHLLAIDHVQKLLYHQVPSEIEGKRTLRAPTFFVSQDDNNFETEFFPRNSEAERRISFFAQSLATPMPEPLPVDNMPTFTVFTPH
RLP61548.1_F641S      EHLLAIDHVQKLLYHQVPSEIEGKRTLRAPTFFVSQDDNNFETEFFPRNSEAERRISFFAQSLATPMPEPLPVDNMPTFTVFTPH
RLP61548.1_P649H      EHLLAIDHVQKLLYHQVPSEIEGKRTLRAPTFFVSQDDNNFETEFFPRNSEAERRISFFAQSLATPMPEPLPVDNMPTFTVFTPH
RLP61548.1_S645F      EHLLAIDHVQKLLYHQVPSEIEGKRTLRAPTFFVSQDDNNFETEFFPRNSEAERRISFFAQSLATPMPEPLPVDNMPTFTVFTPH
RLP61548.1_S645P      EHLLAIDHVQKLLYHQVPSEIEGKRTLRAPTFFVSQDDNNFETEFFPRNSEAERRISFFAQSLATPMPEPLPVDNMPTFTVFTPH
RLP61548.1_S645Y      EHLLAIDHVQKLLYHQVPSEIEGKRTLRAPTFFVSQDDNNFETEFFPRNSEAERRISFFAQSLATPMPEPLPVDNMPTFTVFTPH

                      851      860       870       880       890       900       910       920       930
ADB43261.1            YSEKILLSLREIIREDDQFSRVTLLEYLKQLHPVEWDCFVKDTKILAEETAAYENGDDSEKLSEDGLKSKIDDLPFYCIGFKSAA
BAA21535.1            YSEKILLSLREIIREDDQFSRVTLLEYLKQLHPVEWDCFVKDTKILAEETAAYENGDDSEKLSEDGLKSKIDDLPFYCIGFKSAA
CAX44487.1            YSEKILLSLREIIREDDQFSRVTLLEYLKQLHPVEWDCFVKDTKILAEETAAYENGDDSEKLSEDGLKSKIDDLPFYCIGFKSAA
KGR15377.1            YSEKILLSLREIIREDDQFSRVTLLEYLKQLHPVEWDCFVKDTKILAEETAAYENGDDSEKLSEDGLKSKIDDLPFYCIGFKSAA
KGR21277.1            YSEKILLSLREIIREDDQFSRVTLLEYLKQLHPVEWDCFVKDTKILAEETAAYENGDDSEKLSEDGLKSKIDDLPFYCIGFKSAA
KGR23302.1            YSEKILLSLREIIREDDQFSRVTLLEYLKQLHPVEWDCFVKDTKILAEETAAYENGDDSEKLSEDGLKSKIDDLPFYCIGFKSAA
RLP61548.1            YSEKILLSLREIIREDDQFSRVTLLEYLKQLHPVEWDCFVKDTKILAEETAAYENGDDSEKLSEDGLKSKIDDLPFYCIGFKSAA
RLP61548.1_D648Y      YSEKILLSLREIIREDDQFSRVTLLEYLKQLHPVEWDCFVKDTKILAEETAAYENGDDSEKLSEDGLKSKIDDLPFYCIGFKSAA
RLP61548.1_F641L      YSEKILLSLREIIREDDQFSRVTLLEYLKQLHPVEWDCFVKDTKILAEETAAYENGDDSEKLSEDGLKSKIDDLPFYCIGFKSAA
RLP61548.1_F641S      YSEKILLSLREIIREDDQFSRVTLLEYLKQLHPVEWDCFVKDTKILAEETAAYENGDDSEKLSEDGLKSKIDDLPFYCIGFKSAA
RLP61548.1_P649H      YSEKILLSLREIIREDDQFSRVTLLEYLKQLHPVEWDCFVKDTKILAEETAAYENGDDSEKLSEDGLKSKIDDLPFYCIGFKSAA
RLP61548.1_S645F      YSEKILLSLREIIREDDQFSRVTLLEYLKQLHPVEWDCFVKDTKILAEETAAYENGDDSEKLSEDGLKSKIDDLPFYCIGFKSAA
RLP61548.1_S645P      YSEKILLSLREIIREDDQFSRVTLLEYLKQLHPVEWDCFVKDTKILAEETAAYENGDDSEKLSEDGLKSKIDDLPFYCIGFKSAA
RLP61548.1_S645Y      YSEKILLSLREIIREDDQFSRVTLLEYLKQLHPVEWDCFVKDTKILAEETAAYENGDDSEKLSEDGLKSKIDDLPFYCIGFKSAA

                          940       950       960       970       980       990      1000      1010      1020
ADB43261.1            PEYTLRTRIWASLRSQTLYRTVSGFMNYARAIKLLYRVENPELVQYFGGDPEGLELALERMARRKFRFLVSMQRLSKFKDDEMEN
BAA21535.1            PEYTLRTRIWASLRSQTLYRTVSGFMNYARAIKLLYRVENPELVQYFGGDPEGLELALERMARRKFRFLVSMQRLSKFKDDEMEN
CAX44487.1            PEYTLRTRIWASLRSQTLYRTVSGFMNYARAIKLLYRVENPELVQYFGGDPEGLELALERMARRKFRFLVSMQRLSKFKDDEMEN
KGR15377.1            PEYTLRTRIWASLRSQTLYRTVSGFMNYARAIKLLYRVENPELVQYFGGDPEGLELALERMARRKFRFLVSMQRLSKFKDDEMEN
KGR21277.1            PEYTLRTRIWASLRSQTLYRTVSGFMNYARAIKLLYRVENPELVQYFGGDPEGLELALERMARRKFRFLVSMQRLSKFKDDEMEN
KGR23302.1            PEYTLRTRIWASLRSQTLYRTVSGFMNYARAIKLLYRVENPELVQYFGGDPEGLELALERMARRKFRFLVSMQRLSKFKDDEMEN
RLP61548.1            PEYTLRTRIWASLRSQTLYRTVSGFMNYARAIKLLYRVENPELVQYFGGDPEGLELALERMARRKFRFLVSMQRLSKFKDDEMEN
RLP61548.1_D648Y      PEYTLRTRIWASLRSQTLYRTVSGFMNYARAIKLLYRVENPELVQYFGGDPEGLELALERMARRKFRFLVSMQRLSKFKDDEMEN
RLP61548.1_F641L      PEYTLRTRIWASLRSQTLYRTVSGFMNYARAIKLLYRVENPELVQYFGGDPEGLELALERMARRKFRFLVSMQRLSKFKDDEMEN
RLP61548.1_F641S      PEYTLRTRIWASLRSQTLYRTVSGFMNYARAIKLLYRVENPELVQYFGGDPEGLELALERMARRKFRFLVSMQRLSKFKDDEMEN
RLP61548.1_P649H      PEYTLRTRIWASLRSQTLYRTVSGFMNYARAIKLLYRVENPELVQYFGGDPEGLELALERMARRKFRFLVSMQRLSKFKDDEMEN
RLP61548.1_S645F      PEYTLRTRIWASLRSQTLYRTVSGFMNYARAIKLLYRVENPELVQYFGGDPEGLELALERMARRKFRFLVSMQRLSKFKDDEMEN
RLP61548.1_S645P      PEYTLRTRIWASLRSQTLYRTVSGFMNYARAIKLLYRVENPELVQYFGGDPEGLELALERMARRKFRFLVSMQRLSKFKDDEMEN
RLP61548.1_S645Y      PEYTLRTRIWASLRSQTLYRTVSGFMNYARAIKLLYRVENPELVQYFGGDPEGLELALERMARRKFRFLVSMQRLSKFKDDEMEN
```

```
                   1021      1030       1040       1050       1060       1070       1080       1090       1100
ADB43261.1         AEFLLRAYPDLQIAYLDEEPALNEDEEPRVYSALIDGHCEMLENGRRRPKFRVQLSGNPILGDGKSDNQNHAVIFHRGEYIQLID
BAA21535.1         AEFLLRAYPDLQIAYLDEEPALNEDEEPRVYSALIDGHCEMLENGRRRPKFRVQLSGNPILGDGKSDNQNHAVIFHRGEYIQLID
CAX44487.1         AEFLLRAYPDLQIAYLDEEPALNEDEEPRVYSALIDGHCEMLENGRRRPKFRVQLSGNPILGDGKSDNQNHAVIFHRGEYIQLID
KGR15377.1         AEFLLRAYPDLQIAYLDEEPALNEDEEPRVYSALIDGHCEMLENGRRRPKFRVQLSGNPILGDGKSDNQNHAVIFHRGEYIQLID
KGR21277.1         AEFLLRAYPDLQIAYLDEEPALNEDEEPRVYSALIDGHCEMLENGRRRPKFRVQLSGNPILGDGKSDNQNHAVIFHRGEYIQLID
KGR23302.1         AEFLLRAYPDLQIAYLDEEPALNEDEEPRVYSALIDGHCEMLENGRRRPKFRVQLSGNPILGDGKSDNQNHAVIFHRGEYIQLID
RLP61548.1         AEFLLRAYPDLQIAYLDEEPALNEDEEPRVYSALIDGHCEMLENGRRRPKFRVQLSGNPILGDGKSDNQNHAVIFHRGEYIQLID
RLP61548.1_D648Y   AEFLLRAYPDLQIAYLDEEPALNEDEEPRVYSALIDGHCEMLENGRRRPKFRVQLSGNPILGDGKSDNQNHAVIFHRGEYIQLID
RLP61548.1_F641L   AEFLLRAYPDLQIAYLDEEPALNEDEEPRVYSALIDGHCEMLENGRRRPKFRVQLSGNPILGDGKSDNQNHAVIFHRGEYIQLID
RLP61548.1_F641S   AEFLLRAYPDLQIAYLDEEPALNEDEEPRVYSALIDGHCEMLENGRRRPKFRVQLSGNPILGDGKSDNQNHAVIFHRGEYIQLID
RLP61548.1_P649H   AEFLLRAYPDLQIAYLDEEPALNEDEEPRVYSALIDGHCEMLENGRRRPKFRVQLSGNPILGDGKSDNQNHAVIFHRGEYIQLID
RLP61548.1_S645F   AEFLLRAYPDLQIAYLDEEPALNEDEEPRVYSALIDGHCEMLENGRRRPKFRVQLSGNPILGDGKSDNQNHAVIFHRGEYIQLID
RLP61548.1_S645P   AEFLLRAYPDLQIAYLDEEPALNEDEEPRVYSALIDGHCEMLENGRRRPKFRVQLSGNPILGDGKSDNQNHAVIFHRGEYIQLID
RLP61548.1_S645Y   AEFLLRAYPDLQIAYLDEEPALNEDEEPRVYSALIDGHCEMLENGRRRPKFRVQLSGNPILGDGKSDNQNHAVIFHRGEYIQLID

                       1110       1120       1130       1140       1150       1160       1170       1180       1190
ADB43261.1         ANQDNYLEECLKIRSVLAEFEEMNVEHVNPYAPNLKSEDNNTKKDPVAFLGAREYIFSENSGVLGDVAAGKEQTFGTLFARTLAQ
BAA21535.1         ANQDNYLEECLKIRSVLAEFEEMNVEHVNPYAPNLKSEDNNTKKDPVAFLGAREYIFSENSGVLGDVAAGKEQTFGTLFARTLAQ
CAX44487.1         ANQDNYLEECLKIRSVLAEFEEMNVEHVNPYAPNLKSEDNSTKKDPVAFLGAREYIFSENSGVLGDVAAGKEQTFGTLFARTLAQ
KGR15377.1         ANQDNYLEECLKIRSVLAEFEEMNVEHVNPYAPNLKSEDNNTKKDPVAFLGAREYIFSENSGVLGDVAAGKEQTFGTLFARTLAQ
KGR21277.1         ANQDNYLEECLKIRSVLAEFEEMNVEHVNPYAPNLKSEDNNTKKDPVAFLGAREYIFSENSGVLGDVAAGKEQTFGTLFARTLAQ
KGR23302.1         ANQDNYLEECLKIRSVLAEFEEMNVEHVNPYAPNLKSEDNNTKKDPVAFLGAREYIFSENSGVLGDVAAGKEQTFGTLFARTLAQ
RLP61548.1         ANQDNYLEECLKIRSVLAEFEEMNVEHVNPYAPNLKSEDNNTKKDPVAFLGAREYIFSENSGVLGDVAAGKEQTFGTLFARTLAQ
RLP61548.1_D648Y   ANQDNYLEECLKIRSVLAEFEEMNVEHVNPYAPNLKSEDNNTKKDPVAFLGAREYIFSENSGVLGDVAAGKEQTFGTLFARTLAQ
RLP61548.1_F641L   ANQDNYLEECLKIRSVLAEFEEMNVEHVNPYAPNLKSEDNNTKKDPVAFLGAREYIFSENSGVLGDVAAGKEQTFGTLFARTLAQ
RLP61548.1_F641S   ANQDNYLEECLKIRSVLAEFEEMNVEHVNPYAPNLKSEDNNTKKDPVAFLGAREYIFSENSGVLGDVAAGKEQTFGTLFARTLAQ
RLP61548.1_P649H   ANQDNYLEECLKIRSVLAEFEEMNVEHVNPYAPNLKSEDNNTKKDPVAFLGAREYIFSENSGVLGDVAAGKEQTFGTLFARTLAQ
RLP61548.1_S645F   ANQDNYLEECLKIRSVLAEFEEMNVEHVNPYAPNLKSEDNNTKKDPVAFLGAREYIFSENSGVLGDVAAGKEQTFGTLFARTLAQ
RLP61548.1_S645P   ANQDNYLEECLKIRSVLAEFEEMNVEHVNPYAPNLKSEDNNTKKDPVAFLGAREYIFSENSGVLGDVAAGKEQTFGTLFARTLAQ
RLP61548.1_S645Y   ANQDNYLEECLKIRSVLAEFEEMNVEHVNPYAPNLKSEDNNTKKDPVAFLGAREYIFSENSGVLGDVAAGKEQTFGTLFARTLAQ

                   1191      1200       1210       1220       1230       1240       1250       1260       1270
ADB43261.1         IGGKLHYGHPDFLNATFMLTRGGVSKAQKGLHLNEDIYAGMNAMMRGGKIKHCEYYQCGKGRDLGFGSILNFTTKIGAGMGEQML
BAA21535.1         IGGKLHYGHPDFLNATFMLTRGGVSKAQKGLHLNEDIYAGMNAMMRGGKIKHCEYYQCGKGRDLGFGSILNFTTKIGAGMGEQML
CAX44487.1         IGGKLHYGHPDFLNATFMLTRGGVSKAQKGLHLNEDIYAGMNAMMRGGKIKHCEYYQCGKGRDLGFGSILNFTTKIGAGMGEQML
KGR15377.1         IGGKLHYGHPDFLNATFMLTRGGVSKAQKGLHLNEDIYAGMNAMMRGGKIKHCEYYQCGKGRDLGFGSILNFTTKIGAGMGEQML
KGR21277.1         IGGKLHYGHPDFLNATFMLTRGGVSKAQKGLHLNEDIYAGMNAMMRGGKIKHCEYYQCGKGRDLGFGSILNFTTKIGAGMGEQML
KGR23302.1         IGGKLHYGHPDFLNATFMLTRGGVSKAQKGLHLNEDIYAGMNAMMRGGKIKHCEYYQCGKGRDLGFGSILNFTTKIGAGMGEQML
RLP61548.1         IGGKLHYGHPDFLNATFMLTRGGVSKAQKGLHLNEDIYAGMNAMMRGGKIKHCEYYQCGKGRDLGFGSILNFTTKIGAGMGEQML
RLP61548.1_D648Y   IGGKLHYGHPDFLNATFMLTRGGVSKAQKGLHLNEDIYAGMNAMMRGGKIKHCEYYQCGKGRDLGFGSILNFTTKIGAGMGEQML
RLP61548.1_F641L   IGGKLHYGHPDFLNATFMLTRGGVSKAQKGLHLNEDIYAGMNAMMRGGKIKHCEYYQCGKGRDLGFGSILNFTTKIGAGMGEQML
RLP61548.1_F641S   IGGKLHYGHPDFLNATFMLTRGGVSKAQKGLHLNEDIYAGMNAMMRGGKIKHCEYYQCGKGRDLGFGSILNFTTKIGAGMGEQML
RLP61548.1_P649H   IGGKLHYGHPDFLNATFMLTRGGVSKAQKGLHLNEDIYAGMNAMMRGGKIKHCEYYQCGKGRDLGFGSILNFTTKIGAGMGEQML
RLP61548.1_S645F   IGGKLHYGHPDFLNATFMLTRGGVSKAQKGLHLNEDIYAGMNAMMRGGKIKHCEYYQCGKGRDLGFGSILNFTTKIGAGMGEQML
RLP61548.1_S645P   IGGKLHYGHPDFLNATFMLTRGGVSKAQKGLHLNEDIYAGMNAMMRGGKIKHCEYYQCGKGRDLGFGSILNFTTKIGAGMGEQML
RLP61548.1_S645Y   IGGKLHYGHPDFLNATFMLTRGGVSKAQKGLHLNEDIYAGMNAMMRGGKIKHCEYYQCGKGRDLGFGSILNFTTKIGAGMGEQML
```

```
                        1280      1290      1300      1310      1320      1330      1340      1350      1360
ADB43261.1              SREYFYLGTQLPLDRFLSFYYGHPGFHINNLFIQLSLQVFILVLANLNSLAHEAIMCSYNKDVPVTDVLYPFGCYNIAPAVDWIR
BAA21535.1              SREYFYLGTQLPLDRFLSFYYGHPGFHINNLFIQLSLQVFILVLGNLNSLAHEAIMCSYNKDVPVTDVLYPFGCYNIAPAVDWIR
CAX44487.1              SREYFYLGTQLPLDRFLSFYYGHPGFHINNLFIQLSLQVFILVLANLNSLAHEAIMCSYNKDVPVTDVLYPFGCYNIAPAVDWIR
KGR15377.1              SREYFYLGTQLPLDRFLSFYYGHPGFHINNLFIQLSLQVFILVLANLNSLAHEAIMCSYNKDVPVTDVLYPFGCYNIAPAVDWIR
KGR21277.1              SREYFYLGTQLPLDRFLSFYYGHPGFHINNLFIQLSLQVFILVLANLNSLAHEAIMCSYNKDVPVTDVLYPFGCYNIAPAVDWIR
KGR23302.1              SREYFYLGTQLPLDRFLSFYYGHPGFHINNLFIQLSLQVFILVLANLNSLAHEAIMCSYNKDVPVTDVLYPFGCYNIAPAVDWIR
RLP61548.1              SREYFYLGTQLPLDRFLSFYYGHPGFHINNLFIQLSLQVFILVLANLNSLAHEAIMCSYNKDVPVTDVLYPFGCYNIAPAVDWIR
RLP61548.1_D648Y        SREYFYLGTQLPLDRFLSFYYGHPGFHINNLFIQLSLQVFILVLANLNSLAHEAIMCSYNKDVPVTDVLYPFGCYNIAPAVDWIR
RLP61548.1_F641L        SREYFYLGTQLPLDRFLSFYYGHPGFHINNLFIQLSLQVFILVLANLNSLAHEAIMCSYNKDVPVTDVLYPFGCYNIAPAVDWIR
RLP61548.1_F641S        SREYFYLGTQLPLDRFLSFYYGHPGFHINNLFIQLSLQVFILVLANLNSLAHEAIMCSYNKDVPVTDVLYPFGCYNIAPAVDWIR
RLP61548.1_P649H        SREYFYLGTQLPLDRFLSFYYGHPGFHINNLFIQLSLQVFILVLANLNSLAHEAIMCSYNKDVPVTDVLYPFGCYNIAPAVDWIR
RLP61548.1_S645F        SREYFYLGTQLPLDRFLSFYYGHPGFHINNLFIQLSLQVFILVLANLNSLAHEAIMCSYNKDVPVTDVLYPFGCYNIAPAVDWIR
RLP61548.1_S645P        SREYFYLGTQLPLDRFLSFYYGHPGFHINNLFIQLSLQVFILVLANLNSLAHEAIMCSYNKDVPVTDVLYPFGCYNIAPAVDWIR
RLP61548.1_S645Y        SREYFYLGTQLPLDRFLSFYYGHPGFHINNLFIQLSLQVFILVLANLNSLAHEAIMCSYNKDVPVTDVLYPFGCYNIAPAVDWIR

                        1361     1370      1380      1390      1400      1410      1420      1430      1440
ADB43261.1              RYTLSIFIVFFISFIPLVVQELIERGVWKAFQRFVRHFISMSPFFEVFVAQIYSSSVFTDLTVGGARYISTGRGFATSRIPFSIL
BAA21535.1              RYTLSIFIVFFISFIPLVVQELIERGVWKAFQRFVRHFISMSPFFEVFVAQIYSSSVFTDLTVGGARYISTGRGFATSRIPFSIL
CAX44487.1              RYTLSIFIVFFISFIPLVVQELIERGVWKAFQRFVRHFISMSPFFEVFVAQIYSSSVFTDLTVGGARYISTGRGFATSRIPFSIL
KGR15377.1              RYTLSIFIVFFISFIPLVVQELIERGVWKAFQRFVRHFISMSPFFEVFVAQIYSSSVFTDLTVGGARYISTGRGFATSRIPFSIL
KGR21277.1              RYTLSIFIVFFISFIPLVVQELIERGVWKAFQRFVRHFISMSPFFEVFVAQIYSSSVFTDLTVGGARYISTGRGFATSRIPFSIL
KGR23302.1              RYTLSIFIVFFISFIPLVVQELIERGVWKAFQRFVRHFISMSPFFEVFVAQIYSSSVFTDLTVGGARYISTGRGFATSRIPFSIL
RLP61548.1              RYTLSIFIVFFISFIPLVVQELIERGVWKAFQRFVRHFISMSPFFEVFVAQIYSSSVFTDLTVGGARYISTGRGFATSRIPFSIL
RLP61548.1_D648Y        RYTLSIFIVFFISFIPLVVQELIERGVWKAFQRFVRHFISMSPFFEVFVAQIYSSSVFTDLTVGGARYISTGRGFATSRIPFSIL
RLP61548.1_F641L        RYTLSIFIVFFISFIPLVVQELIERGVWKAFQRFVRHFISMSPFFEVFVAQIYSSSVFTDLTVGGARYISTGRGFATSRIPFSIL
RLP61548.1_F641S        RYTLSIFIVFFISFIPLVVQELIERGVWKAFQRFVRHFISMSPFFEVFVAQIYSSSVFTDLTVGGARYISTGRGFATSRIPFSIL
RLP61548.1_P649H        RYTLSIFIVFFISFIPLVVQELIERGVWKAFQRFVRHFISMSPFFEVFVAQIYSSSVFTDLTVGGARYISTGRGFATSRIPFSIL
RLP61548.1_S645F        RYTLSIFIVFFISFIPLVVQELIERGVWKAFQRFVRHFISMSPFFEVFVAQIYSSSVFTDLTVGGARYISTGRGFATSRIPFSIL
RLP61548.1_S645P        RYTLSIFIVFFISFIPLVVQELIERGVWKAFQRFVRHFISMSPFFEVFVAQIYSSSVFTDLTVGGARYISTGRGFATSRIPFSIL
RLP61548.1_S645Y        RYTLSIFIVFFISFIPLVVQELIERGVWKAFQRFVRHFISMSPFFEVFVAQIYSSSVFTDLTVGGARYISTGRGFATSRIPFSIL

                        1450      1460      1470      1480      1490      1500      1510      1520      1530
ADB43261.1              YSRFADSSIYMGARLMLILLFGTVSHWQAPLLWFWASLSALMFSPFIFNPHQFAWEDFFLDYRDFIRWLSRGNTKWHRNSWIGYV
BAA21535.1              YSRFADSSIYMGARLMLILLFGTVSHWQAPLLWFWASLSALMFSPFIFNPHQFAWEDFFLDYRDFIRWLSRGNTKWHRNSWIGYV
CAX44487.1              YSRFADSSIYMGARLMLILLFGTVSHWQAPLLWFWASLSALMFSPFIFNPHQFAWEDFFLDYRDFIRWLSRGNTKWHRNSWIGYV
KGR15377.1              YSRFADSSIYMGARLMLILLFGTVSHWQAPLLWFWASLSALMFSPFIFNPHQFAWEDFFLDYRDFIRWLSRGNTKWHRNSWIGYV
KGR21277.1              YSRFADSSIYMGARLMLILLFGTVSHWQAPLLWFWASLSALMFSPFIFNPHQFAWEDFFLDYRDFIRWLSRGNTKWHRNSWIGYV
KGR23302.1              YSRFADSSIYMGARLMLILLFGTVSHWQAPLLWFWASLSALMFSPFIFNPHQFAWEDFFLDYRDFIRWLSRGNTKWHRNSWIGYV
RLP61548.1              YSRFADSSIYMGARLMLILLFGTVSHWQAPLLWFWASLSALMFSPFIFNPHQFAWEDFFLDYRDFIRWLSRGNTKWHRNSWIGYV
RLP61548.1_D648Y        YSRFADSSIYMGARLMLILLFGTVSHWQAPLLWFWASLSALMFSPFIFNPHQFAWEDFFLDYRDFIRWLSRGNTKWHRNSWIGYV
RLP61548.1_F641L        YSRFADSSIYMGARLMLILLFGTVSHWQAPLLWFWASLSALMFSPFIFNPHQFAWEDFFLDYRDFIRWLSRGNTKWHRNSWIGYV
RLP61548.1_F641S        YSRFADSSIYMGARLMLILLFGTVSHWQAPLLWFWASLSALMFSPFIFNPHQFAWEDFFLDYRDFIRWLSRGNTKWHRNSWIGYV
RLP61548.1_P649H        YSRFADSSIYMGARLMLILLFGTVSHWQAPLLWFWASLSALMFSPFIFNPHQFAWEDFFLDYRDFIRWLSRGNTKWHRNSWIGYV
RLP61548.1_S645F        YSRFADSSIYMGARLMLILLFGTVSHWQAPLLWFWASLSALMFSPFIFNPHQFAWEDFFLDYRDFIRWLSRGNTKWHRNSWIGYV
RLP61548.1_S645P        YSRFADSSIYMGARLMLILLFGTVSHWQAPLLWFWASLSALMFSPFIFNPHQFAWEDFFLDYRDFIRWLSRGNTKWHRNSWIGYV
RLP61548.1_S645Y        YSRFADSSIYMGARLMLILLFGTVSHWQAPLLWFWASLSALMFSPFIFNPHQFAWEDFFLDYRDFIRWLSRGNTKWHRNSWIGYV
```

```
                      1531      1540      1550      1560      1570      1580      1590      1600      1610
ADB43261.1            RLSRSRITGFKRKLTGDVSEKAAGDASRAHRSNVLFADFLPTLIYTAGLYVAYTFINAQTGVTSYPYEINGSTDPQPVNSTLRLI
BAA21535.1            RLSRSRITGFKRKLTGDVSEKAAGDASRAHRSNVLFADFLPTLIYTAGLYVAYTFINAQTGVTSYPYEINGSTDPQPVNSTLRLI
CAX44487.1            RLSRSRITGFKRKLTGDVSEKAAGDASRAHRSNVLFADFLPTLIYTAGLYVAYTFINAQTGVTSYPYEILGSTDPQPVNSTLRLI
KGR15377.1            RLSRSRITGFKRKLTGDVSEKAAGDASRAHRSNVLFADFLPTLIYTAGLYVAYTFINAQTGVTSYPYEINGSTDPQPVNSTLRLI
KGR21277.1            RLSRSRITGFKRKLTGDVSEKAAGDASRAHRSNVLFADFLPTLIYTAGLYVAYTFINAQTGVTSYPYEINGSTDPQPVNSTLRLI
KGR23302.1            RLSRSRITGFKRKLTGDVSEKAAGDASRAHRSNVLFADFLPTLIYTAGLYVAYTFINAQTGVTSYPYEINGSTDPQPVNSTLRLI
RLP61548.1            RLSRSRITGFKRKLTGDVSEKAAGDASRAHRSNVLFADFLPTLIYTAGLYVAYTFINAQTGVTSYPYEINGSTDPQPVNSTLRLI
RLP61548.1_D648Y      RLSRSRITGFKRKLTGDVSEKAAGDASRAHRSNVLFADFLPTLIYTAGLYVAYTFINAQTGVTSYPYEINGSTDPQPVNSTLRLI
RLP61548.1_F641L      RLSRSRITGFKRKLTGDVSEKAAGDASRAHRSNVLFADFLPTLIYTAGLYVAYTFINAQTGVTSYPYEINGSTDPQPVNSTLRLI
RLP61548.1_F641S      RLSRSRITGFKRKLTGDVSEKAAGDASRAHRSNVLFADFLPTLIYTAGLYVAYTFINAQTGVTSYPYEINGSTDPQPVNSTLRLI
RLP61548.1_P649H      RLSRSRITGFKRKLTGDVSEKAAGDASRAHRSNVLFADFLPTLIYTAGLYVAYTFINAQTGVTSYPYEINGSTDPQPVNSTLRLI
RLP61548.1_S645F      RLSRSRITGFKRKLTGDVSEKAAGDASRAHRSNVLFADFLPTLIYTAGLYVAYTFINAQTGVTSYPYEINGSTDPQPVNSTLRLI
RLP61548.1_S645P      RLSRSRITGFKRKLTGDVSEKAAGDASRAHRSNVLFADFLPTLIYTAGLYVAYTFINAQTGVTSYPYEINGSTDPQPVNSTLRLI
RLP61548.1_S645Y      RLSRSRITGFKRKLTGDVSEKAAGDASRAHRSNVLFADFLPTLIYTAGLYVAYTFINAQTGVTSYPYEINGSTDPQPVNSTLRLI
```

```
                          1620      1630      1640      1650      1660      1670      1680      1690      1700
ADB43261.1            ICALAPVVIDMGCLGVCLAMACCAGPMLGLCCKKTGAVIAGVAHGVAVIVHIIFFIVMWVTEGFNFARLMLGIATMIYVQRLLFK
BAA21535.1            ICALAPVVIDMGCLGVCLAMACCAGPMLGLCCKKTGAVIAGVAHGVAVIVHIIFFIVMWVTEGFNFARLMLGIATMIYVQRLLFK
CAX44487.1            ICSLAPVVIDMGCLGVCLAMACCAGPMLGLCCKKTGAVIAGVAHGVAVIVHIIFFIVMWVTEGFNFARLMLGIATMIYVQRLLFK
KGR15377.1            ICALAPVVIDMGCLGVCLAMACCAGPMLGLCCKKTGAVIAGVAHGVAVIVHIIFFIVMWVTEGFNFARLMLGIATMIYVQRLLFK
KGR21277.1            ICALAPVVIDMGCLGVCLAMACCAGPMLGLCCKKTGAVIAGVAHGVAVIVHIIFFIVMWVTEGFNFARLMLGIATMIYVQRLLFK
KGR23302.1            ICALAPVVIDMGCLGVCLAMACCAGPMLGLCCKKTGAVIAGVAHGVAVIVHIIFFIVMWVTEGFNFARLMLGIATMIYVQRLLFK
RLP61548.1            ICALAPVVIDMGCLGVCLAMACCAGPMLGLCCKKTGAVIAGVAHGVAVIVHIIFFIVMWVTEGFNFARLMLGIATMIYVQRLLFK
RLP61548.1_D648Y      ICALAPVVIDMGCLGVCLAMACCAGPMLGLCCKKTGAVIAGVAHGVAVIVHIIFFIVMWVTEGFNFARLMLGIATMIYVQRLLFK
RLP61548.1_F641L      ICALAPVVIDMGCLGVCLAMACCAGPMLGLCCKKTGAVIAGVAHGVAVIVHIIFFIVMWVTEGFNFARLMLGIATMIYVQRLLFK
RLP61548.1_F641S      ICALAPVVIDMGCLGVCLAMACCAGPMLGLCCKKTGAVIAGVAHGVAVIVHIIFFIVMWVTEGFNFARLMLGIATMIYVQRLLFK
RLP61548.1_P649H      ICALAPVVIDMGCLGVCLAMACCAGPMLGLCCKKTGAVIAGVAHGVAVIVHIIFFIVMWVTEGFNFARLMLGIATMIYVQRLLFK
RLP61548.1_S645F      ICALAPVVIDMGCLGVCLAMACCAGPMLGLCCKKTGAVIAGVAHGVAVIVHIIFFIVMWVTEGFNFARLMLGIATMIYVQRLLFK
RLP61548.1_S645P      ICALAPVVIDMGCLGVCLAMACCAGPMLGLCCKKTGAVIAGVAHGVAVIVHIIFFIVMWVTEGFNFARLMLGIATMIYVQRLLFK
RLP61548.1_S645Y      ICALAPVVIDMGCLGVCLAMACCAGPMLGLCCKKTGAVIAGVAHGVAVIVHIIFFIVMWVTEGFNFARLMLGIATMIYVQRLLFK
```

```
                      1701      1710      1720      1730      1740      1750      1760      1770      1780
ADB43261.1            FLTLCFLTREFKNDKANTAFWTGKWYNTGMGWMAFTQPSREFVAKIIEMSEFAGDFVLAHIILFCQLPFLFIPLVDRWHSMMLFW
BAA21535.1            FLTLCFLTREFKNDKANTAFWTGKWYNTGMGWMAFTQPSREFVAKIIEMSEFAGDFVLAHIILFCQLPLLFIPLVDRWHSMMLFW
CAX44487.1            FLTLCFLTREFKNDKANTAFWTGKWYNTGMGWMAFTQPAREFVAKIIEMSEFAGDFVLAHIILFCQLPFLFIPLVDRWHSMMLFW
KGR15377.1            FLTLCFLTREFKNDKANTAFWTGKWYNTGMGWMAFTQPSREFVAKIIEMSEFAGDFVLAHIILFCQLPFLFIPLVDRWHSMMLFW
KGR21277.1            FLTLCFLTREFKNDKANTAFWTGKWYNTGMGWMAFTQPSREFVAKIIEMSEFAGDFVLAHIILFCQLPFLFIPLVDRWHSMMLFW
KGR23302.1            FLTLCFLTREFKNDKANTAFWTGKWYNTGMGWMAFTQPSREFVAKIIEMSEFAGDFVLAHIILFCQLPFLFIPLVDRWHSMMLFW
RLP61548.1            FLTLCFLTREFKNDKANTAFWTGKWYNTGMGWMAFTQPSREFVAKIIEMSEFAGDFVLAHIILFCQLPFLFIPLVDRWHSMMLFW
RLP61548.1_D648Y      FLTLCFLTREFKNDKANTAFWTGKWYNTGMGWMAFTQPSREFVAKIIEMSEFAGDFVLAHIILFCQLPFLFIPLVDRWHSMMLFW
RLP61548.1_F641L      FLTLCFLTREFKNDKANTAFWTGKWYNTGMGWMAFTQPSREFVAKIIEMSEFAGDFVLAHIILFCQLPFLFIPLVDRWHSMMLFW
RLP61548.1_F641S      FLTLCFLTREFKNDKANTAFWTGKWYNTGMGWMAFTQPSREFVAKIIEMSEFAGDFVLAHIILFCQLPFLFIPLVDRWHSMMLFW
RLP61548.1_P649H      FLTLCFLTREFKNDKANTAFWTGKWYNTGMGWMAFTQPSREFVAKIIEMSEFAGDFVLAHIILFCQLPFLFIPLVDRWHSMMLFW
RLP61548.1_S645F      FLTLCFLTREFKNDKANTAFWTGKWYNTGMGWMAFTQPSREFVAKIIEMSEFAGDFVLAHIILFCQLPFLFIPLVDRWHSMMLFW
RLP61548.1_S645P      FLTLCFLTREFKNDKANTAFWTGKWYNTGMGWMAFTQPSREFVAKIIEMSEFAGDFVLAHIILFCQLPFLFIPLVDRWHSMMLFW
RLP61548.1_S645Y      FLTLCFLTREFKNDKANTAFWTGKWYNTGMGWMAFTQPSREFVAKIIEMSEFAGDFVLAHIILFCQLPFLFIPLVDRWHSMMLFW
```

	1790 1800 1810 1820 1830 1840 1850 1860 1870
ADB43261.1	LKPSRLIRPPIYSLKQARLRKRMVRKYCVLYFAVLILFIVIIVAPAVASGQIXVDQFANIGGSGSIADGLFQPRNVSNNDTGNHR
BAA21535.1	LKPSRLIRPPIYSLKQARLRKRMVRKYCVLYFAVLILFIVIIVAPAVASGQIAVDQFANIGGSGSIADGLFQPRNVSNNDTGNHR
CAX44487.1	LKPSRLIRPPIYSLKQARLRKRMVRKYCVLYFAVLILFIVIIVAPAVASGQIAVDQFANIGGSGSIANGLFQPRNVSNNDTGNHR
KGR15377.1	LKPSRLIRPPIYSLKQARLRKRMVRKYCVLYFAVLILFIVIIVAPAVASGQIPVDQFANIGGSGSIADGLFQPRNVSNNDTGNHR
KGR21277.1	LKPSRLIRPPIYSLKQARLRKRMVRKYCVLYFAVLILFIVIIVAPAVASGQIAVDQFANIGGSGSIADGLFQPRNVSNNDTGNHR
KGR23302.1	LKPSRLIRPPIYSLKQARLRKRMVRKYCVLYFAVLILFIVIIVAPAVASGQIPVDQFANIGGSGSIADGLFQPRNVSNNDTGNHR
RLP61548.1	LKPSRLIRPPIYSLKQARLRKRMVRKYCVLYFAVLILFIVIIVAPAVASGQIAVDQFANIGGSGSIADGLFQPRNVSNNDTGNHR
RLP61548.1_D648Y	LKPSRLIRPPIYSLKQARLRKRMVRKYCVLYFAVLILFIVIIVAPAVASGQIAVDQFANIGGSGSIADGLFQPRNVSNNDTGNHR
RLP61548.1_F641L	LKPSRLIRPPIYSLKQARLRKRMVRKYCVLYFAVLILFIVIIVAPAVASGQIAVDQFANIGGSGSIADGLFQPRNVSNNDTGNHR
RLP61548.1_F641S	LKPSRLIRPPIYSLKQARLRKRMVRKYCVLYFAVLILFIVIIVAPAVASGQIAVDQFANIGGSGSIADGLFQPRNVSNNDTGNHR
RLP61548.1_P649H	LKPSRLIRPPIYSLKQARLRKRMVRKYCVLYFAVLILFIVIIVAPAVASGQIAVDQFANIGGSGSIADGLFQPRNVSNNDTGNHR
RLP61548.1_S645F	LKPSRLIRPPIYSLKQARLRKRMVRKYCVLYFAVLILFIVIIVAPAVASGQIAVDQFANIGGSGSIADGLFQPRNVSNNDTGNHR
RLP61548.1_S645P	LKPSRLIRPPIYSLKQARLRKRMVRKYCVLYFAVLILFIVIIVAPAVASGQIAVDQFANIGGSGSIADGLFQPRNVSNNDTGNHR
RLP61548.1_S645Y	LKPSRLIRPPIYSLKQARLRKRMVRKYCVLYFAVLILFIVIIVAPAVASGQIAVDQFANIGGSGSIADGLFQPRNVSNNDTGNHR

	1871 1880 1890
ADB43261.1	PKTYTWSYLSTRFTGSTTPYSTNPFRV
BAA21535.1	PKTYTWSYLSTRFTGSTTPYSTNPFRV
CAX44487.1	PKSYSWTYLSTRFTGSTTHYSTNPFRI
KGR15377.1	PKTYTWSYLSTRFTGTTTPYSTNPFRV
KGR21277.1	PKTYTWSYLSTRFTGSTTPYSTNPFRV
KGR23302.1	PKTYTWSYLSTRFTGTTTPYSTNPFRV
RLP61548.1	PKTYTWSYLSTRFTGSTTPYSTNPFRV
RLP61548.1_D648Y	PKTYTWSYLSTRFTGSTTPYSTNPFRV
RLP61548.1_F641L	PKTYTWSYLSTRFTGSTTPYSTNPFRV
RLP61548.1_F641S	PKTYTWSYLSTRFTGSTTPYSTNPFRV
RLP61548.1_P649H	PKTYTWSYLSTRFTGSTTPYSTNPFRV
RLP61548.1_S645F	PKTYTWSYLSTRFTGSTTPYSTNPFRV
RLP61548.1_S645P	PKTYTWSYLSTRFTGSTTPYSTNPFRV
RLP61548.1_S645Y	PKTYTWSYLSTRFTGSTTPYSTNPFRV

Table S4. SSSCPreds data of FKS1 for *Candida glabrata*.

	1 10 20 30 40 50 60 70 80
KAI8383591.1	MSYDQGGNGNWQNTDPNGNYYYNGAENNEFYDQD
	YASQQPEQQQGGEGYYDEYGQPNYNYMNDPQQGQMPQQQPGGYENDGYYDSYYNNQMNAGVGNGLGPDQTNFSDFSSYGPPPFQN
KAI8387093.1	MSYNNNGQQMSDQSYYDNNQGYYQPEDQQNGQAMYGDEGYYDPNISGGDYYNQPPPPPNMMGQDMENFSDFSSYGPPGVQGYDYQ
KAI8387093.1_D632E	MSYNNNGQQMSDQSYYDNNQGYYQPEDQQNGQAMYGDEGYYDPNISGGDYYNQPPPPPNMMGQDMENFSDFSSYGPPGVQGYDYQ
KAI8387093.1_D632G	MSYNNNGQQMSDQSYYDNNQGYYQPEDQQNGQAMYGDEGYYDPNISGGDYYNQPPPPPNMMGQDMENFSDFSSYGPPGVQGYDYQ
KAI8387093.1_D632H	MSYNNNGQQMSDQSYYDNNQGYYQPEDQQNGQAMYGDEGYYDPNISGGDYYNQPPPPPNMMGQDMENFSDFSSYGPPGVQGYDYQ
KAI8387093.1_D632Y	MSYNNNGQQMSDQSYYDNNQGYYQPEDQQNGQAMYGDEGYYDPNISGGDYYNQPPPPPNMMGQDMENFSDFSSYGPPGVQGYDYQ
KAI8387093.1_F625C	MSYNNNGQQMSDQSYYDNNQGYYQPEDQQNGQAMYGDEGYYDPNISGGDYYNQPPPPPNMMGQDMENFSDFSSYGPPGVQGYDYQ
KAI8387093.1_F625S	MSYNNNGQQMSDQSYYDNNQGYYQPEDQQNGQAMYGDEGYYDPNISGGDYYNQPPPPPNMMGQDMENFSDFSSYGPPGVQGYDYQ
KAI8387093.1_I634V	MSYNNNGQQMSDQSYYDNNQGYYQPEDQQNGQAMYGDEGYYDPNISGGDYYNQPPPPPNMMGQDMENFSDFSSYGPPGVQGYDYQ
KAI8387093.1_S629F	MSYNNNGQQMSDQSYYDNNQGYYQPEDQQNGQAMYGDEGYYDPNISGGDYYNQPPPPPNMMGQDMENFSDFSSYGPPGVQGYDYQ
KAI8387093.1_S629P	MSYNNNGQQMSDQSYYDNNQGYYQPEDQQNGQAMYGDEGYYDPNISGGDYYNQPPPPPNMMGQDMENFSDFSSYGPPGVQGYDYQ
KAI8397588.1	MSYNNNGQQMSDQGYYDNNQGYYQPEDQQNGQAMYGDEGYYDPNISGGDYYNQPPPPPNMMGQDMENFSDFSSYGPPGVQGYDYQ

	90 100 110 120 130 140 150 160 170
KAI8383591.1	NQANYTPSQLSYSNNGMGSNGMNMSGSSTPVYGNYDPNAIAMTLPNDPYPAWTADPQSPVSIEQIEDVFIDLTNKFGFQRDSMRN
KAI8387093.1	NMNQSGQYTPSQMSYNGDPNSGSSTPIYGNGMSAYDPNAIAMALPNDPYPAWTADSQSPVPIEQIEDVFIDLTNKFGFQRDSMRN
KAI8387093.1_D632E	NMNQSGQYTPSQMSYNGDPNSGSSTPIYGNGMSAYDPNAIAMALPNDPYPAWTADSQSPVPIEQIEDVFIDLTNKFGFQRDSMRN
KAI8387093.1_D632G	NMNQSGQYTPSQMSYNGDPNSGSSTPIYGNGMSAYDPNAIAMALPNDPYPAWTADSQSPVPIEQIEDVFIDLTNKFGFQRDSMRN
KAI8387093.1_D632H	NMNQSGQYTPSQMSYNGDPNSGSSTPIYGNGMSAYDPNAIAMALPNDPYPAWTADSQSPVPIEQIEDVFIDLTNKFGFQRDSMRN
KAI8387093.1_D632Y	NMNQSGQYTPSQMSYNGDPNSGSSTPIYGNGMSAYDPNAIAMALPNDPYPAWTADSQSPVPIEQIEDVFIDLTNKFGFQRDSMRN
KAI8387093.1_F625C	NMNQSGQYTPSQMSYNGDPNSGSSTPIYGNGMSAYDPNAIAMALPNDPYPAWTADSQSPVPIEQIEDVFIDLTNKFGFQRDSMRN
KAI8387093.1_F625S	NMNQSGQYTPSQMSYNGDPNSGSSTPIYGNGMSAYDPNAIAMALPNDPYPAWTADSQSPVPIEQIEDVFIDLTNKFGFQRDSMRN
KAI8387093.1_I634V	NMNQSGQYTPSQMSYNGDPNSGSSTPIYGNGMSAYDPNAIAMALPNDPYPAWTADSQSPVPIEQIEDVFIDLTNKFGFQRDSMRN
KAI8387093.1_S629F	NMNQSGQYTPSQMSYNGDPNSGSSTPIYGNGMSAYDPNAIAMALPNDPYPAWTADSQSPVPIEQIEDVFIDLTNKFGFQRDSMRN
KAI8387093.1_S629P	NMNQSGQYTPSQMSYNGDPNSGSSTPIYGNGMSAYDPNAIAMALPNDPYPAWTADSQSPVPIEQIEDVFIDLTNKFGFQRDSMRN
KAI8397588.1	NMNQSGQYTPSQMSYNGDPNSGSSTPIYGNGMSAYDPNAIAMALPNDPYPAWTADSQSPVPIEQIEDVFIDLTNKFGFQRDSMRN

	171 180 190 200 210 220 230 240 250
KAI8383591.1	IFDLFMTLLDSRTSRMSPDQALLSVHADYIGGDTANYKKWYFAAQLDMDDEVGFRNMNLGKLSRKARKAKKKNKKAMEEANPEDA
KAI8387093.1	MFDHFMTLLDSRTSRMAPDQALLSLHADYIGGDTANYKKWYFAAQLDMDDEVGFRNMSLGKLSRKARKAKKKNKKAMEEANPEDA
KAI8387093.1_D632E	MFDHFMTLLDSRTSRMAPDQALLSLHADYIGGDTANYKKWYFAAQLDMDDEVGFRNMSLGKLSRKARKAKKKNKKAMEEANPEDA
KAI8387093.1_D632G	MFDHFMTLLDSRTSRMAPDQALLSLHADYIGGDTANYKKWYFAAQLDMDDEVGFRNMSLGKLSRKARKAKKKNKKAMEEANPEDA
KAI8387093.1_D632H	MFDHFMTLLDSRTSRMAPDQALLSLHADYIGGDTANYKKWYFAAQLDMDDEVGFRNMSLGKLSRKARKAKKKNKKAMEEANPEDA
KAI8387093.1_D632Y	MFDHFMTLLDSRTSRMAPDQALLSLHADYIGGDTANYKKWYFAAQLDMDDEVGFRNMSLGKLSRKARKAKKKNKKAMEEANPEDA
KAI8387093.1_F625C	MFDHFMTLLDSRTSRMAPDQALLSLHADYIGGDTANYKKWYFAAQLDMDDEVGFRNMSLGKLSRKARKAKKKNKKAMEEANPEDA
KAI8387093.1_F625S	MFDHFMTLLDSRTSRMAPDQALLSLHADYIGGDTANYKKWYFAAQLDMDDEVGFRNMSLGKLSRKARKAKKKNKKAMEEANPEDA
KAI8387093.1_I634V	MFDHFMTLLDSRTSRMAPDQALLSLHADYIGGDTANYKKWYFAAQLDMDDEVGFRNMSLGKLSRKARKAKKKNKKAMEEANPEDA
KAI8387093.1_S629F	MFDHFMTLLDSRTSRMAPDQALLSLHADYIGGDTANYKKWYFAAQLDMDDEVGFRNMSLGKLSRKARKAKKKNKKAMEEANPEDA
KAI8387093.1_S629P	MFDHFMTLLDSRTSRMAPDQALLSLHADYIGGDTANYKKWYFAAQLDMDDEVGFRNMSLGKLSRKARKAKKKNKKAMEEANPEDA
KAI8397588.1	MFDHFMTLLDSRTSRMAPDQALLSLHADYIGGDTANYKKWYFAAQLDMDDEVGFRNMSLGKLSRKARKAKKKNKKAMEEANPEDA

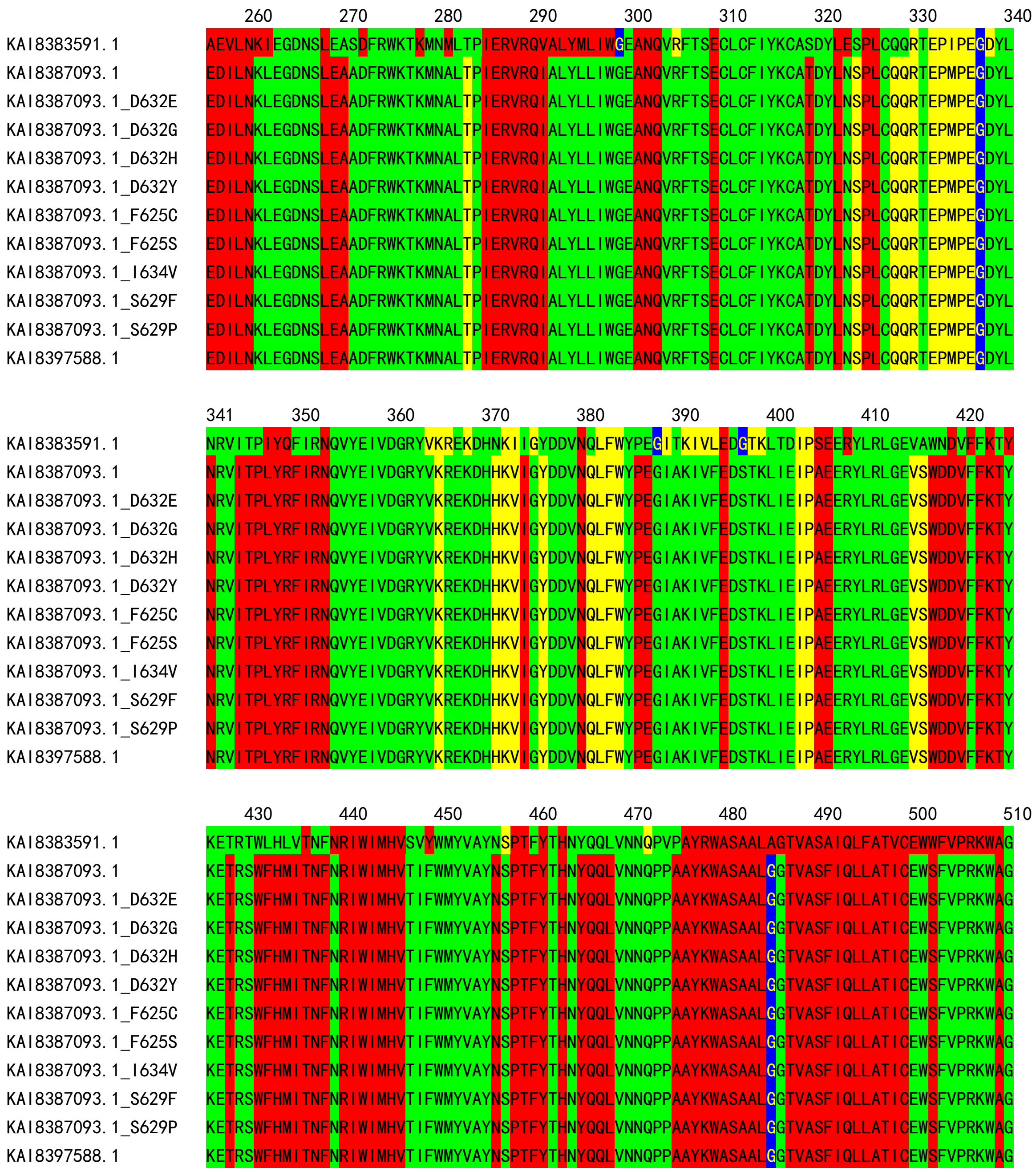
260 270 280 290 300 310 320 330 340
KAI8383591.1 AEVLNKIEGDNSLEASDFRWKTKMNMLTPIERVRQVALYMLIWGEANQVRFTSECLCFIYKCASDYLESPLCQQRTEPIPEGDYL
KAI8387093.1 EDILNKLEGDNSLEAADFRWKTKMNALTPIERVRQIALYLLIWGEANQVRFTSECLCFIYKCATDYLNSPLCQQRTEPMPEGDYL
KAI8387093.1_D632E EDILNKLEGDNSLEAADFRWKTKMNALTPIERVRQIALYLLIWGEANQVRFTSECLCFIYKCATDYLNSPLCQQRTEPMPEGDYL
KAI8387093.1_D632G EDILNKLEGDNSLEAADFRWKTKMNALTPIERVRQIALYLLIWGEANQVRFTSECLCFIYKCATDYLNSPLCQQRTEPMPEGDYL
KAI8387093.1_D632H EDILNKLEGDNSLEAADFRWKTKMNALTPIERVRQIALYLLIWGEANQVRFTSECLCFIYKCATDYLNSPLCQQRTEPMPEGDYL
KAI8387093.1_D632Y EDILNKLEGDNSLEAADFRWKTKMNALTPIERVRQIALYLLIWGEANQVRFTSECLCFIYKCATDYLNSPLCQQRTEPMPEGDYL
KAI8387093.1_F625C EDILNKLEGDNSLEAADFRWKTKMNALTPIERVRQIALYLLIWGEANQVRFTSECLCFIYKCATDYLNSPLCQQRTEPMPEGDYL
KAI8387093.1_F625S EDILNKLEGDNSLEAADFRWKTKMNALTPIERVRQIALYLLIWGEANQVRFTSECLCFIYKCATDYLNSPLCQQRTEPMPEGDYL
KAI8387093.1_I634V EDILNKLEGDNSLEAADFRWKTKMNALTPIERVRQIALYLLIWGEANQVRFTSECLCFIYKCATDYLNSPLCQQRTEPMPEGDYL
KAI8387093.1_S629F EDILNKLEGDNSLEAADFRWKTKMNALTPIERVRQIALYLLIWGEANQVRFTSECLCFIYKCATDYLNSPLCQQRTEPMPEGDYL
KAI8387093.1_S629P EDILNKLEGDNSLEAADFRWKTKMNALTPIERVRQIALYLLIWGEANQVRFTSECLCFIYKCATDYLNSPLCQQRTEPMPEGDYL
KAI8397588.1 EDILNKLEGDNSLEAADFRWKTKMNALTPIERVRQIALYLLIWGEANQVRFTSECLCFIYKCATDYLNSPLCQQRTEPMPEGDYL
341 350 360 370 380 390 400 410 420
KAI8383591.1 NRVITPIYQFIRNQVYEIVDGRYVKREKDHNKIIGYDDVNQLFWYPEGITKIVLEDGTKLTDIPSEERYLRLGEVAWNDVFFKTY
KAI8387093.1 NRVITPLYRFIRNQVYEIVDGRYVKREKDHHKVIGYDDVNQLFWYPEGIAKIVFEDSTKLIEIPAEERYLRLGEVSWDDVFFKTY
KAI8387093.1_D632E NRVITPLYRFIRNQVYEIVDGRYVKREKDHHKVIGYDDVNQLFWYPEGIAKIVFEDSTKLIEIPAEERYLRLGEVSWDDVFFKTY
KAI8387093.1_D632G NRVITPLYRFIRNQVYEIVDGRYVKREKDHHKVIGYDDVNQLFWYPEGIAKIVFEDSTKLIEIPAEERYLRLGEVSWDDVFFKTY
KAI8387093.1_D632H NRVITPLYRFIRNQVYEIVDGRYVKREKDHHKVIGYDDVNQLFWYPEGIAKIVFEDSTKLIEIPAEERYLRLGEVSWDDVFFKTY
KAI8387093.1_D632Y NRVITPLYRFIRNQVYEIVDGRYVKREKDHHKVIGYDDVNQLFWYPEGIAKIVFEDSTKLIEIPAEERYLRLGEVSWDDVFFKTY
KAI8387093.1_F625C NRVITPLYRFIRNQVYEIVDGRYVKREKDHHKVIGYDDVNQLFWYPEGIAKIVFEDSTKLIEIPAEERYLRLGEVSWDDVFFKTY
KAI8387093.1_F625S NRVITPLYRFIRNQVYEIVDGRYVKREKDHHKVIGYDDVNQLFWYPEGIAKIVFEDSTKLIEIPAEERYLRLGEVSWDDVFFKTY
KAI8387093.1_I634V NRVITPLYRFIRNQVYEIVDGRYVKREKDHHKVIGYDDVNQLFWYPEGIAKIVFEDSTKLIEIPAEERYLRLGEVSWDDVFFKTY
KAI8387093.1_S629F NRVITPLYRFIRNQVYEIVDGRYVKREKDHHKVIGYDDVNQLFWYPEGIAKIVFEDSTKLIEIPAEERYLRLGEVSWDDVFFKTY
KAI8387093.1_S629P NRVITPLYRFIRNQVYEIVDGRYVKREKDHHKVIGYDDVNQLFWYPEGIAKIVFEDSTKLIEIPAEERYLRLGEVSWDDVFFKTY
KAI8397588.1 NRVITPLYRFIRNQVYEIVDGRYVKREKDHHKVIGYDDVNQLFWYPEGIAKIVFEDSTKLIEIPAEERYLRLGEVSWDDVFFKTY
430 440 450 460 470 480 490 500 510
KAI8383591.1 KETRTWLHLVTNFNRIWIMHVSVYWMYVAYNSPTFYTHNYQQLVNNQPVPAYRWASAALAGTVASAIQLFATVCEWWFVPRKWAG
KAI8387093.1 KETRSWFHMITNFNRIWIMHVTIFWMYVAYNSPTFYTHNYQQLVNNQPPAAYKWASAALGGTVASFIQLLATICEWSFVPRKWAG
KAI8387093.1_D632E KETRSWFHMITNFNRIWIMHVTIFWMYVAYNSPTFYTHNYQQLVNNQPPAAYKWASAALGGTVASFIQLLATICEWSFVPRKWAG
KAI8387093.1_D632G KETRSWFHMITNFNRIWIMHVTIFWMYVAYNSPTFYTHNYQQLVNNQPPAAYKWASAALGGTVASFIQLLATICEWSFVPRKWAG
KAI8387093.1_D632H KETRSWFHMITNFNRIWIMHVTIFWMYVAYNSPTFYTHNYQQLVNNQPPAAYKWASAALGGTVASFIQLLATICEWSFVPRKWAG
KAI8387093.1_D632Y KETRSWFHMITNFNRIWIMHVTIFWMYVAYNSPTFYTHNYQQLVNNQPPAAYKWASAALGGTVASFIQLLATICEWSFVPRKWAG
KAI8387093.1_F625C KETRSWFHMITNFNRIWIMHVTIFWMYVAYNSPTFYTHNYQQLVNNQPPAAYKWASAALGGTVASFIQLLATICEWSFVPRKWAG
KAI8387093.1_F625S KETRSWFHMITNFNRIWIMHVTIFWMYVAYNSPTFYTHNYQQLVNNQPPAAYKWASAALGGTVASFIQLLATICEWSFVPRKWAG
KAI8387093.1_I634V KETRSWFHMITNFNRIWIMHVTIFWMYVAYNSPTFYTHNYQQLVNNQPPAAYKWASAALGGTVASFIQLLATICEWSFVPRKWAG
KAI8387093.1_S629F KETRSWFHMITNFNRIWIMHVTIFWMYVAYNSPTFYTHNYQQLVNNQPPAAYKWASAALGGTVASFIQLLATICEWSFVPRKWAG
KAI8387093.1_S629P KETRSWFHMITNFNRIWIMHVTIFWMYVAYNSPTFYTHNYQQLVNNQPPAAYKWASAALGGTVASFIQLLATICEWSFVPRKWAG
KAI8397588.1 KETRSWFHMITNFNRIWIMHVTIFWMYVAYNSPTFYTHNYQQLVNNQPPAAYKWASAALGGTVASFIQLLATICEWSFVPRKWAG

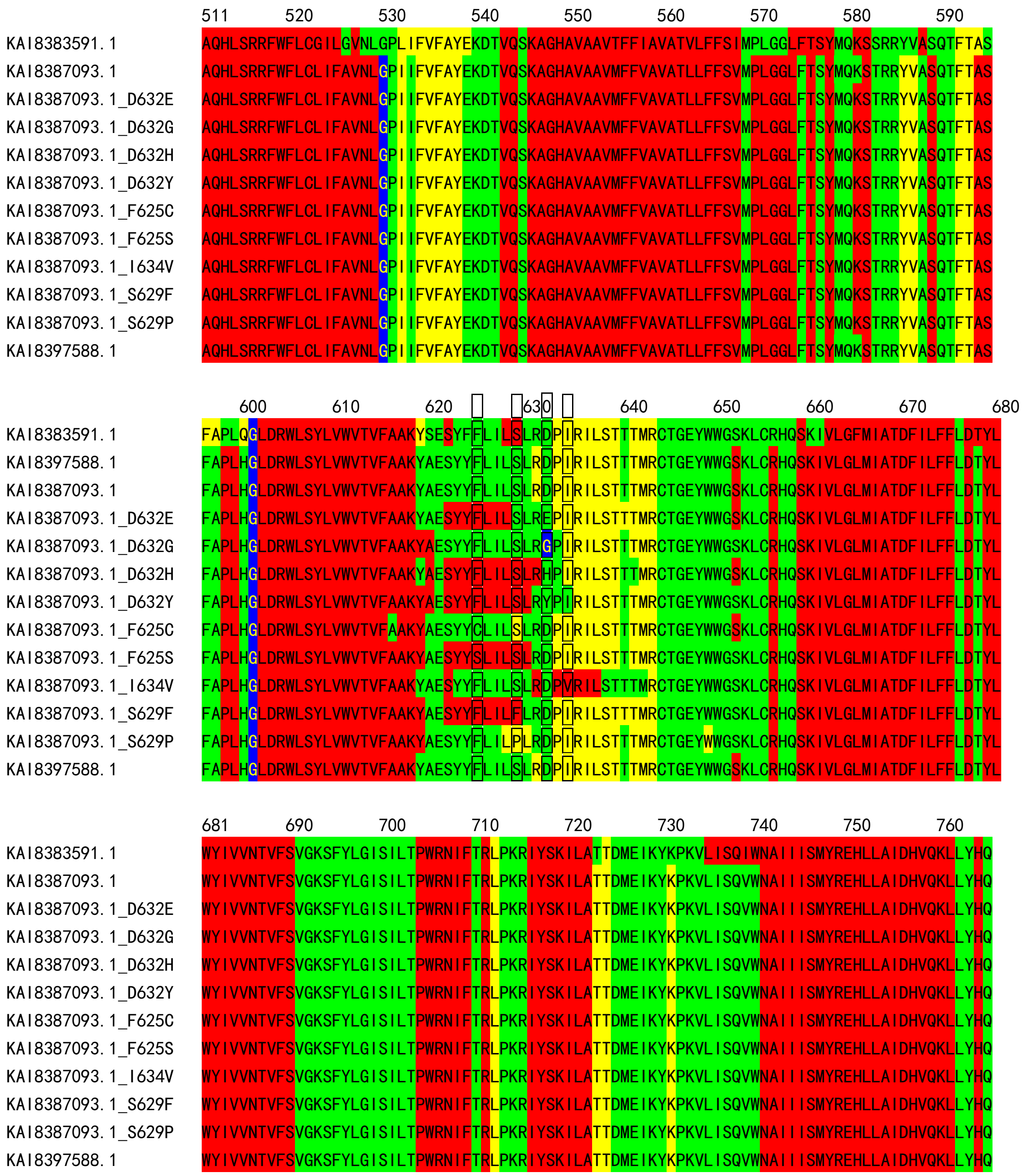
511 520 530 540 550 560 570 580 590
KAI8383591.1 AQHLSRRFWFLCGILGVNLGPLIFVFAYEKDTVQSKAGHAVAAVTFFIAVATVLFFSIMPLGGLFTSYMQKSSRRYVASQTFTAS
KAI8387093.1 AQHLSRRFWFLCLIFAVNLGPIIFVFAYEKDTVQSKAGHAVAAVMFFVAVATLLFFSVMPLGGLFTSYMQKSTRRYVASQTFTAS
KAI8387093.1_D632E AQHLSRRFWFLCLIFAVNLGPIIFVFAYEKDTVQSKAGHAVAAVMFFVAVATLLFFSVMPLGGLFTSYMQKSTRRYVASQTFTAS
KAI8387093.1_D632G AQHLSRRFWFLCLIFAVNLGPIIFVFAYEKDTVQSKAGHAVAAVMFFVAVATLLFFSVMPLGGLFTSYMQKSTRRYVASQTFTAS
KAI8387093.1_D632H AQHLSRRFWFLCLIFAVNLGPIIFVFAYEKDTVQSKAGHAVAAVMFFVAVATLLFFSVMPLGGLFTSYMQKSTRRYVASQTFTAS
KAI8387093.1_D632Y AQHLSRRFWFLCLIFAVNLGPIIFVFAYEKDTVQSKAGHAVAAVMFFVAVATLLFFSVMPLGGLFTSYMQKSTRRYVASQTFTAS
KAI8387093.1_F625C AQHLSRRFWFLCLIFAVNLGPIIFVFAYEKDTVQSKAGHAVAAVMFFVAVATLLFFSVMPLGGLFTSYMQKSTRRYVASQTFTAS
KAI8387093.1_F625S AQHLSRRFWFLCLIFAVNLGPIIFVFAYEKDTVQSKAGHAVAAVMFFVAVATLLFFSVMPLGGLFTSYMQKSTRRYVASQTFTAS
KAI8387093.1_I634V AQHLSRRFWFLCLIFAVNLGPIIFVFAYEKDTVQSKAGHAVAAVMFFVAVATLLFFSVMPLGGLFTSYMQKSTRRYVASQTFTAS
KAI8387093.1_S629F AQHLSRRFWFLCLIFAVNLGPIIFVFAYEKDTVQSKAGHAVAAVMFFVAVATLLFFSVMPLGGLFTSYMQKSTRRYVASQTFTAS
KAI8387093.1_S629P AQHLSRRFWFLCLIFAVNLGPIIFVFAYEKDTVQSKAGHAVAAVMFFVAVATLLFFSVMPLGGLFTSYMQKSTRRYVASQTFTAS
KAI8397588.1 AQHLSRRFWFLCLIFAVNLGPIIFVFAYEKDTVQSKAGHAVAAVMFFVAVATLLFFSVMPLGGLFTSYMQKSTRRYVASQTFTAS
600 610 620 630 640 650 660 670 680
KAI8383591.1 FAPLQGLDRWLSYLVWVTVFAAKYSESYFFLILSLRDPIRILSTTTMRCTGEYWWGSKLCRHQSKIVLGFMIATDFILFFLDTYL
KAI8397588.1 FAPLHGLDRWLSYLVWVTVFAAKYAESYYFLILSLRDPIRILSTTTMRCTGEYWWGSKLCRHQSKIVLGLMIATDFILFFLDTYL
KAI8387093.1 FAPLHGLDRWLSYLVWVTVFAAKYAESYYFLILSLRDPIRILSTTTMRCTGEYWWGSKLCRHQSKIVLGLMIATDFILFFLDTYL
KAI8387093.1_D632E FAPLHGLDRWLSYLVWVTVFAAKYAESYYFLILSLREPIRILSTTTMRCTGEYWWGSKLCRHQSKIVLGLMIATDFILFFLDTYL
KAI8387093.1_D632G FAPLHGLDRWLSYLVWVTVFAAKYAESYYFLILSLRGPIRILSTTTMRCTGEYWWGSKLCRHQSKIVLGLMIATDFILFFLDTYL
KAI8387093.1_D632H FAPLHGLDRWLSYLVWVTVFAAKYAESYYFLILSLRHPIRILSTTTMRCTGEYWWGSKLCRHQSKIVLGLMIATDFILFFLDTYL
KAI8387093.1_D632Y FAPLHGLDRWLSYLVWVTVFAAKYAESYYFLILSLRYPIRILSTTTMRCTGEYWWGSKLCRHQSKIVLGLMIATDFILFFLDTYL
KAI8387093.1_F625C FAPLHGLDRWLSYLVWVTVFAAKYAESYYCLILSLRDPIRILSTTTMRCTGEYWWGSKLCRHQSKIVLGLMIATDFILFFLDTYL
KAI8387093.1_F625S FAPLHGLDRWLSYLVWVTVFAAKYAESYYSLILSLRDPIRILSTTTMRCTGEYWWGSKLCRHQSKIVLGLMIATDFILFFLDTYL
KAI8387093.1_I634V FAPLHGLDRWLSYLVWVTVFAAKYAESYYFLILSLRDPVRILSTTTMRCTGEYWWGSKLCRHQSKIVLGLMIATDFILFFLDTYL
KAI8387093.1_S629F FAPLHGLDRWLSYLVWVTVFAAKYAESYYFLILFLRDPIRILSTTTMRCTGEYWWGSKLCRHQSKIVLGLMIATDFILFFLDTYL
KAI8387093.1_S629P FAPLHGLDRWLSYLVWVTVFAAKYAESYYFLILPLRDPIRILSTTTMRCTGEYWWGSKLCRHQSKIVLGLMIATDFILFFLDTYL
KAI8397588.1 FAPLHGLDRWLSYLVWVTVFAAKYAESYYFLILSLRDPIRILSTTTMRCTGEYWWGSKLCRHQSKIVLGLMIATDFILFFLDTYL
681 690 700 710 720 730 740 750 760
KAI8383591.1 WYIVVNTVFSVGKSFYLGISILTPWRNIFTRLPKRIYSKILATTDMEIKYKPKVLISQIWNAIIISMYREHLLAIDHVQKLLYHQ
KAI8387093.1 WYIVVNTVFSVGKSFYLGISILTPWRNIFTRLPKRIYSKILATTDMEIKYKPKVLISQVWNAIIISMYREHLLAIDHVQKLLYHQ
KAI8387093.1_D632E WYIVVNTVFSVGKSFYLGISILTPWRNIFTRLPKRIYSKILATTDMEIKYKPKVLISQVWNAIIISMYREHLLAIDHVQKLLYHQ
KAI8387093.1_D632G WYIVVNTVFSVGKSFYLGISILTPWRNIFTRLPKRIYSKILATTDMEIKYKPKVLISQVWNAIIISMYREHLLAIDHVQKLLYHQ
KAI8387093.1_D632H WYIVVNTVFSVGKSFYLGISILTPWRNIFTRLPKRIYSKILATTDMEIKYKPKVLISQVWNAIIISMYREHLLAIDHVQKLLYHQ
KAI8387093.1_D632Y WYIVVNTVFSVGKSFYLGISILTPWRNIFTRLPKRIYSKILATTDMEIKYKPKVLISQVWNAIIISMYREHLLAIDHVQKLLYHQ
KAI8387093.1_F625C WYIVVNTVFSVGKSFYLGISILTPWRNIFTRLPKRIYSKILATTDMEIKYKPKVLISQVWNAIIISMYREHLLAIDHVQKLLYHQ
KAI8387093.1_F625S WYIVVNTVFSVGKSFYLGISILTPWRNIFTRLPKRIYSKILATTDMEIKYKPKVLISQVWNAIIISMYREHLLAIDHVQKLLYHQ
KAI8387093.1_I634V WYIVVNTVFSVGKSFYLGISILTPWRNIFTRLPKRIYSKILATTDMEIKYKPKVLISQVWNAIIISMYREHLLAIDHVQKLLYHQ
KAI8387093.1_S629F WYIVVNTVFSVGKSFYLGISILTPWRNIFTRLPKRIYSKILATTDMEIKYKPKVLISQVWNAIIISMYREHLLAIDHVQKLLYHQ
KAI8387093.1_S629P WYIVVNTVFSVGKSFYLGISILTPWRNIFTRLPKRIYSKILATTDMEIKYKPKVLISQVWNAIIISMYREHLLAIDHVQKLLYHQ
KAI8397588.1 WYIVVNTVFSVGKSFYLGISILTPWRNIFTRLPKRIYSKILATTDMEIKYKPKVLISQVWNAIIISMYREHLLAIDHVQKLLYHQ

```
                           770       780       790       800       810       820       830       840       850
KAI8383591.1               VPSEIEGKRTLRAPTFFVSQDDNNFETEFFPRNSEAERRISFFAQSLATPMPEPLPVDNMPTFTVLTPHYSERILLSLREIIRED
KAI8387093.1               VPSEIEGKRTLRAPTFFVSQDDNNFETEFFPRNSEAERRISFFAQSLATPMPEPLPVDNMPTFTVLTPHYAERILLSLREIIRED
KAI8387093.1_D632E         VPSEIEGKRTLRAPTFFVSQDDNNFETEFFPRNSEAERRISFFAQSLATPMPEPLPVDNMPTFTVLTPHYAERILLSLREIIRED
KAI8387093.1_D632G         VPSEIEGKRTLRAPTFFVSQDDNNFETEFFPRNSEAERRISFFAQSLATPMPEPLPVDNMPTFTVLTPHYAERILLSLREIIRED
KAI8387093.1_D632H         VPSEIEGKRTLRAPTFFVSQDDNNFETEFFPRNSEAERRISFFAQSLATPMPEPLPVDNMPTFTVLTPHYAERILLSLREIIRED
KAI8387093.1_D632Y         VPSEIEGKRTLRAPTFFVSQDDNNFETEFFPRNSEAERRISFFAQSLATPMPEPLPVDNMPTFTVLTPHYAERILLSLREIIRED
KAI8387093.1_F625C         VPSEIEGKRTLRAPTFFVSQDDNNFETEFFPRNSEAERRISFFAQSLATPMPEPLPVDNMPTFTVLTPHYAERILLSLREIIRED
KAI8387093.1_F625S         VPSEIEGKRTLRAPTFFVSQDDNNFETEFFPRNSEAERRISFFAQSLATPMPEPLPVDNMPTFTVLTPHYAERILLSLREIIRED
KAI8387093.1_I634V         VPSEIEGKRTLRAPTFFVSQDDNNFETEFFPRNSEAERRISFFAQSLATPMPEPLPVDNMPTFTVLTPHYAERILLSLREIIRED
KAI8387093.1_S629F         VPSEIEGKRTLRAPTFFVSQDDNNFETEFFPRNSEAERRISFFAQSLATPMPEPLPVDNMPTFTVLTPHYAERILLSLREIIRED
KAI8387093.1_S629P         VPSEIEGKRTLRAPTFFVSQDDNNFETEFFPRNSEAERRISFFAQSLATPMPEPLPVDNMPTFTVLTPHYAERILLSLREIIRED
KAI8397588.1               VPSEIEGKRTLRAPTFFVSQDDNNFETEFFPRNSEAERRISFFAQSLATPMPEPLPVDNMPTFTVLTPHYAERILLSLREIIRED

                           851       860       870       880       890       900       910       920       930
KAI8383591.1               DQFSRVTLLEYLKQLHPVEWECFVKDTKILAEETAAYENEEPQDPEKSDALKTQIDDLPFYCIGFKSAAPEYTLRTRIWASLRSQ
KAI8387093.1               DQFSRVTLLEYLKQLHPVEWECFVKDTKILAEETAAYEGMDDQDPEKEDALKNQIDDLPFYCIGFKSAAPEYTLRTRIWASLRSQ
KAI8387093.1_D632E         DQFSRVTLLEYLKQLHPVEWECFVKDTKILAEETAAYEGMDDQDPEKEDALKNQIDDLPFYCIGFKSAAPEYTLRTRIWASLRSQ
KAI8387093.1_D632G         DQFSRVTLLEYLKQLHPVEWECFVKDTKILAEETAAYEGMDDQDPEKEDALKNQIDDLPFYCIGFKSAAPEYTLRTRIWASLRSQ
KAI8387093.1_D632H         DQFSRVTLLEYLKQLHPVEWECFVKDTKILAEETAAYEGMDDQDPEKEDALKNQIDDLPFYCIGFKSAAPEYTLRTRIWASLRSQ
KAI8387093.1_D632Y         DQFSRVTLLEYLKQLHPVEWECFVKDTKILAEETAAYEGMDDQDPEKEDALKNQIDDLPFYCIGFKSAAPEYTLRTRIWASLRSQ
KAI8387093.1_F625C         DQFSRVTLLEYLKQLHPVEWECFVKDTKILAEETAAYEGMDDQDPEKEDALKNQIDDLPFYCIGFKSAAPEYTLRTRIWASLRSQ
KAI8387093.1_F625S         DQFSRVTLLEYLKQLHPVEWECFVKDTKILAEETAAYEGMDDQDPEKEDALKNQIDDLPFYCIGFKSAAPEYTLRTRIWASLRSQ
KAI8387093.1_I634V         DQFSRVTLLEYLKQLHPVEWECFVKDTKILAEETAAYEGMDDQDPEKEDALKNQIDDLPFYCIGFKSAAPEYTLRTRIWASLRSQ
KAI8387093.1_S629F         DQFSRVTLLEYLKQLHPVEWECFVKDTKILAEETAAYEGMDDQDPEKEDALKNQIDDLPFYCIGFKSAAPEYTLRTRIWASLRSQ
KAI8387093.1_S629P         DQFSRVTLLEYLKQLHPVEWECFVKDTKILAEETAAYEGMDDQDPEKEDALKNQIDDLPFYCIGFKSAAPEYTLRTRIWASLRSQ
KAI8397588.1               DQFSRVTLLEYLKQLHPVEWECFVKDTKILAEETAAYEGMDDQDPEKEDALKNQIDDLPFYCIGFKSAAPEYTLRTRIWASLRSQ

                           940       950       960       970       980       990       1000      1010      1020
KAI8383591.1               TLYRTVSGFMNYARAIKLLYRVENPEIVQMFGGNAEGLERELEKMARRKFKFLVSMQRLAKFKPHELENTEFLLRAYPDLQIAYL
KAI8387093.1               TLYRTVSGFMNYARAIKLLYRVENPEIVQMFGGNAEGLERELEKMARRKFKFLVSMQRLAKFKPHELENAEFLLRAYPDLQIAYL
KAI8387093.1_D632E         TLYRTVSGFMNYARAIKLLYRVENPEIVQMFGGNAEGLERELEKMARRKFKFLVSMQRLAKFKPHELENAEFLLRAYPDLQIAYL
KAI8387093.1_D632G         TLYRTVSGFMNYARAIKLLYRVENPEIVQMFGGNAEGLERELEKMARRKFKFLVSMQRLAKFKPHELENAEFLLRAYPDLQIAYL
KAI8387093.1_D632H         TLYRTVSGFMNYARAIKLLYRVENPEIVQMFGGNAEGLERELEKMARRKFKFLVSMQRLAKFKPHELENAEFLLRAYPDLQIAYL
KAI8387093.1_D632Y         TLYRTVSGFMNYARAIKLLYRVENPEIVQMFGGNAEGLERELEKMARRKFKFLVSMQRLAKFKPHELENAEFLLRAYPDLQIAYL
KAI8387093.1_F625C         TLYRTVSGFMNYARAIKLLYRVENPEIVQMFGGNAEGLERELEKMARRKFKFLVSMQRLAKFKPHELENAEFLLRAYPDLQIAYL
KAI8387093.1_F625S         TLYRTVSGFMNYARAIKLLYRVENPEIVQMFGGNAEGLERELEKMARRKFKFLVSMQRLAKFKPHELENAEFLLRAYPDLQIAYL
KAI8387093.1_I634V         TLYRTVSGFMNYARAIKLLYRVENPEIVQMFGGNAEGLERELEKMARRKFKFLVSMQRLAKFKPHELENAEFLLRAYPDLQIAYL
KAI8387093.1_S629F         TLYRTVSGFMNYARAIKLLYRVENPEIVQMFGGNAEGLERELEKMARRKFKFLVSMQRLAKFKPHELENAEFLLRAYPDLQIAYL
KAI8387093.1_S629P         TLYRTVSGFMNYARAIKLLYRVENPEIVQMFGGNAEGLERELEKMARRKFKFLVSMQRLAKFKPHELENAEFLLRAYPDLQIAYL
KAI8397588.1               TLYRTVSGFMNYARAIKLLYRVENPEIVQMFGGNAEGLERELEKMARRKFKFLVSMQRLAKFKPHELENAEFLLRAYPDLQIAYL
```

```
                        1021      1030      1040      1050      1060      1070      1080      1090      1100
KAI8383591.1            DEEPPLNEGEEPRIYSALIDGHCEMLENGRRRPKFRVQLSGNPILGDGKSDNQNHALIFYRGEYIQLIDANQDNYLEECLKIRSV
KAI8387093.1            DEEPPLNEGEEPRIYSALIDGHCEILENGRRRPKFRVQLSGNPILGDGKSDNQNHALIFYRGEYIQLIDANQDNYLEECLKIRSV
KAI8387093.1_D632E      DEEPPLNEGEEPRIYSALIDGHCEILENGRRRPKFRVQLSGNPILGDGKSDNQNHALIFYRGEYIQLIDANQDNYLEECLKIRSV
KAI8387093.1_D632G      DEEPPLNEGEEPRIYSALIDGHCEILENGRRRPKFRVQLSGNPILGDGKSDNQNHALIFYRGEYIQLIDANQDNYLEECLKIRSV
KAI8387093.1_D632H      DEEPPLNEGEEPRIYSALIDGHCEILENGRRRPKFRVQLSGNPILGDGKSDNQNHALIFYRGEYIQLIDANQDNYLEECLKIRSV
KAI8387093.1_D632Y      DEEPPLNEGEEPRIYSALIDGHCEILENGRRRPKFRVQLSGNPILGDGKSDNQNHALIFYRGEYIQLIDANQDNYLEECLKIRSV
KAI8387093.1_F625C      DEEPPLNEGEEPRIYSALIDGHCEILENGRRRPKFRVQLSGNPILGDGKSDNQNHALIFYRGEYIQLIDANQDNYLEECLKIRSV
KAI8387093.1_F625S      DEEPPLNEGEEPRIYSALIDGHCEILENGRRRPKFRVQLSGNPILGDGKSDNQNHALIFYRGEYIQLIDANQDNYLEECLKIRSV
KAI8387093.1_I634V      DEEPPLNEGEEPRIYSALIDGHCEILENGRRRPKFRVQLSGNPILGDGKSDNQNHALIFYRGEYIQLIDANQDNYLEECLKIRSV
KAI8387093.1_S629F      DEEPPLNEGEEPRIYSALIDGHCEILENGRRRPKFRVQLSGNPILGDGKSDNQNHALIFYRGEYIQLIDANQDNYLEECLKIRSV
KAI8387093.1_S629P      DEEPPLNEGEEPRIYSALIDGHCEILENGRRRPKFRVQLSGNPILGDGKSDNQNHALIFYRGEYIQLIDANQDNYLEECLKIRSV
KAI8397588.1            DEEPPLNEGEEPRIYSALIDGHCEILENGRRRPKFRVQLSGNPILGDGKSDNQNHALIFYRGEYIQLIDANQDNYLEECLKIRSV
```

```
                            1110      1120      1130      1140      1150      1160      1170      1180      1190
KAI8383591.1            LAEFEELNAEPVYPYTPGVKYEDQKTNHPVAIVGAREYIFSENSGVLGDVAAGKEQTFGTLFARTLAQIGGKLHYGHPDFINATF
KAI8387093.1            LAEFEELNAEQVYPYSPGVKYEDQNTNHPVAIVGAREYIFSENSGVLGDVAAGKEQTFGTLFARTLAQIGGKLHYGHPDFINATF
KAI8387093.1_D632E      LAEFEELNAEQVYPYSPGVKYEDQNTNHPVAIVGAREYIFSENSGVLGDVAAGKEQTFGTLFARTLAQIGGKLHYGHPDFINATF
KAI8387093.1_D632G      LAEFEELNAEQVYPYSPGVKYEDQNTNHPVAIVGAREYIFSENSGVLGDVAAGKEQTFGTLFARTLAQIGGKLHYGHPDFINATF
KAI8387093.1_D632H      LAEFEELNAEQVYPYSPGVKYEDQNTNHPVAIVGAREYIFSENSGVLGDVAAGKEQTFGTLFARTLAQIGGKLHYGHPDFINATF
KAI8387093.1_D632Y      LAEFEELNAEQVYPYSPGVKYEDQNTNHPVAIVGAREYIFSENSGVLGDVAAGKEQTFGTLFARTLAQIGGKLHYGHPDFINATF
KAI8387093.1_F625C      LAEFEELNAEQVYPYSPGVKYEDQNTNHPVAIVGAREYIFSENSGVLGDVAAGKEQTFGTLFARTLAQIGGKLHYGHPDFINATF
KAI8387093.1_F625S      LAEFEELNAEQVYPYSPGVKYEDQNTNHPVAIVGAREYIFSENSGVLGDVAAGKEQTFGTLFARTLAQIGGKLHYGHPDFINATF
KAI8387093.1_I634V      LAEFEELNAEQVYPYSPGVKYEDQNTNHPVAIVGAREYIFSENSGVLGDVAAGKEQTFGTLFARTLAQIGGKLHYGHPDFINATF
KAI8387093.1_S629F      LAEFEELNAEQVYPYSPGVKYEDQNTNHPVAIVGAREYIFSENSGVLGDVAAGKEQTFGTLFARTLAQIGGKLHYGHPDFINATF
KAI8387093.1_S629P      LAEFEELNAEQVYPYSPGVKYEDQNTNHPVAIVGAREYIFSENSGVLGDVAAGKEQTFGTLFARTLAQIGGKLHYGHPDFINATF
KAI8397588.1            LAEFEELNAEQVYPYSPGVKYEDQNTNHPVAIVGAREYIFSENSGVLGDVAAGKEQTFGTLFARTLAQIGGKLHYGHPDFINATF
```

```
                        1191      1200      1210      1220      1230      1240      1250      1260      1270
KAI8383591.1            MTTRSGLSKAQKGLHLNEDIYAGMNALLRGGRIKHCEYYQCGKGRDLGFGTILNFTTKIGAGMGEQMLSREYYYLGTQLPVDRFL
KAI8387093.1            MTTRGGISKAQKGLHLNEDIYAGMNALLRGGRIKHCEYYQCGKGRDLGFGTILNFTTKIGAGMGEQMLSREYYYLGTQLPIDRFL
KAI8387093.1_D632E      MTTRGGISKAQKGLHLNEDIYAGMNALLRGGRIKHCEYYQCGKGRDLGFGTILNFTTKIGAGMGEQMLSREYYYLGTQLPIDRFL
KAI8387093.1_D632G      MTTRGGISKAQKGLHLNEDIYAGMNALLRGGRIKHCEYYQCGKGRDLGFGTILNFTTKIGAGMGEQMLSREYYYLGTQLPIDRFL
KAI8387093.1_D632H      MTTRGGISKAQKGLHLNEDIYAGMNALLRGGRIKHCEYYQCGKGRDLGFGTILNFTTKIGAGMGEQMLSREYYYLGTQLPIDRFL
KAI8387093.1_D632Y      MTTRGGISKAQKGLHLNEDIYAGMNALLRGGRIKHCEYYQCGKGRDLGFGTILNFTTKIGAGMGEQMLSREYYYLGTQLPIDRFL
KAI8387093.1_F625C      MTTRGGISKAQKGLHLNEDIYAGMNALLRGGRIKHCEYYQCGKGRDLGFGTILNFTTKIGAGMGEQMLSREYYYLGTQLPIDRFL
KAI8387093.1_F625S      MTTRGGISKAQKGLHLNEDIYAGMNALLRGGRIKHCEYYQCGKGRDLGFGTILNFTTKIGAGMGEQMLSREYYYLGTQLPIDRFL
KAI8387093.1_I634V      MTTRGGISKAQKGLHLNEDIYAGMNALLRGGRIKHCEYYQCGKGRDLGFGTILNFTTKIGAGMGEQMLSREYYYLGTQLPIDRFL
KAI8387093.1_S629F      MTTRGGISKAQKGLHLNEDIYAGMNALLRGGRIKHCEYYQCGKGRDLGFGTILNFTTKIGAGMGEQMLSREYYYLGTQLPIDRFL
KAI8387093.1_S629P      MTTRGGISKAQKGLHLNEDIYAGMNALLRGGRIKHCEYYQCGKGRDLGFGTILNFTTKIGAGMGEQMLSREYYYLGTQLPIDRFL
KAI8397588.1            MTTRGGISKAQKGLHLNEDIYAGMNALLRGGRIKHCEYYQCGKGRDLGFGTILNFTTKIGAGMGEQMLSREYYYLGTQLPIDRFL
```

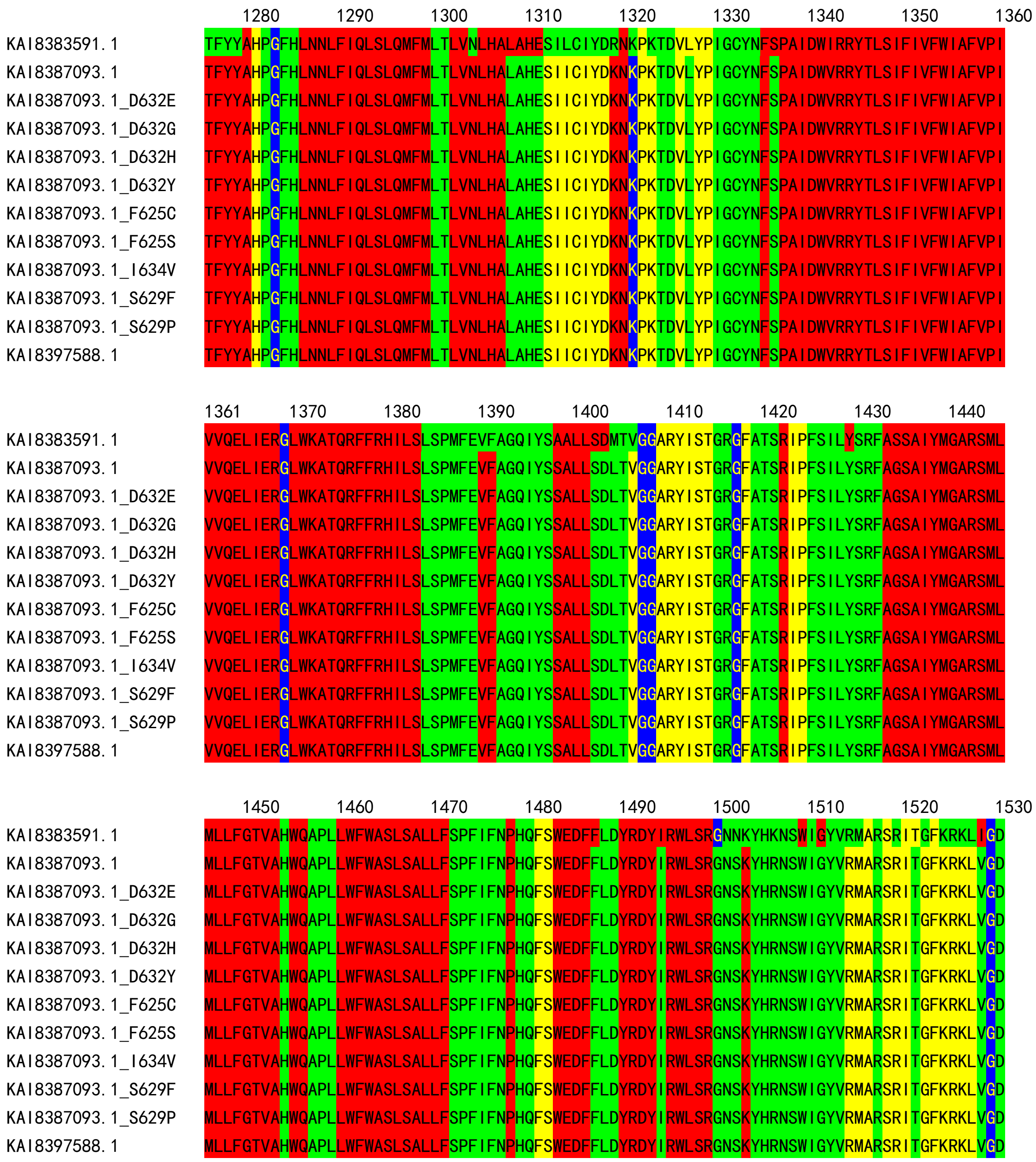
1280 1290 1300 1310 1320 1330 1340 1350 1360
KAI8383591.1 TFYYAHPGFHLNNLFIQLSLQMFMLTLVNLHALAHESILCIYDRNKPKTDVLYPIGCYNFSPAIDWIRRYTLSIFIVFWIAFVPI
KAI8387093.1 TFYYAHPGFHLNNLFIQLSLQMFMLTLVNLHALAHESIICIYDKNKPKTDVLYPIGCYNFSPAIDWVRRYTLSIFIVFWIAFVPI
KAI8387093.1_D632E TFYYAHPGFHLNNLFIQLSLQMFMLTLVNLHALAHESIICIYDKNKPKTDVLYPIGCYNFSPAIDWVRRYTLSIFIVFWIAFVPI
KAI8387093.1_D632G TFYYAHPGFHLNNLFIQLSLQMFMLTLVNLHALAHESIICIYDKNKPKTDVLYPIGCYNFSPAIDWVRRYTLSIFIVFWIAFVPI
KAI8387093.1_D632H TFYYAHPGFHLNNLFIQLSLQMFMLTLVNLHALAHESIICIYDKNKPKTDVLYPIGCYNFSPAIDWVRRYTLSIFIVFWIAFVPI
KAI8387093.1_D632Y TFYYAHPGFHLNNLFIQLSLQMFMLTLVNLHALAHESIICIYDKNKPKTDVLYPIGCYNFSPAIDWVRRYTLSIFIVFWIAFVPI
KAI8387093.1_F625C TFYYAHPGFHLNNLFIQLSLQMFMLTLVNLHALAHESIICIYDKNKPKTDVLYPIGCYNFSPAIDWVRRYTLSIFIVFWIAFVPI
KAI8387093.1_F625S TFYYAHPGFHLNNLFIQLSLQMFMLTLVNLHALAHESIICIYDKNKPKTDVLYPIGCYNFSPAIDWVRRYTLSIFIVFWIAFVPI
KAI8387093.1_I634V TFYYAHPGFHLNNLFIQLSLQMFMLTLVNLHALAHESIICIYDKNKPKTDVLYPIGCYNFSPAIDWVRRYTLSIFIVFWIAFVPI
KAI8387093.1_S629F TFYYAHPGFHLNNLFIQLSLQMFMLTLVNLHALAHESIICIYDKNKPKTDVLYPIGCYNFSPAIDWVRRYTLSIFIVFWIAFVPI
KAI8387093.1_S629P TFYYAHPGFHLNNLFIQLSLQMFMLTLVNLHALAHESIICIYDKNKPKTDVLYPIGCYNFSPAIDWVRRYTLSIFIVFWIAFVPI
KAI8397588.1 TFYYAHPGFHLNNLFIQLSLQMFMLTLVNLHALAHESIICIYDKNKPKTDVLYPIGCYNFSPAIDWVRRYTLSIFIVFWIAFVPI
1361 1370 1380 1390 1400 1410 1420 1430 1440
KAI8383591.1 VVQELIERGLWKATQRFFRHILSLSPMFEVFAGQIYSAALLSDMTVGGARYISTGRGFATSRIPFSILYSRFASSAIYMGARSML
KAI8387093.1 VVQELIERGLWKATQRFFRHILSLSPMFEVFAGQIYSSALLSDLTVGGARYISTGRGFATSRIPFSILYSRFAGSAIYMGARSML
KAI8387093.1_D632E VVQELIERGLWKATQRFFRHILSLSPMFEVFAGQIYSSALLSDLTVGGARYISTGRGFATSRIPFSILYSRFAGSAIYMGARSML
KAI8387093.1_D632G VVQELIERGLWKATQRFFRHILSLSPMFEVFAGQIYSSALLSDLTVGGARYISTGRGFATSRIPFSILYSRFAGSAIYMGARSML
KAI8387093.1_D632H VVQELIERGLWKATQRFFRHILSLSPMFEVFAGQIYSSALLSDLTVGGARYISTGRGFATSRIPFSILYSRFAGSAIYMGARSML
KAI8387093.1_D632Y VVQELIERGLWKATQRFFRHILSLSPMFEVFAGQIYSSALLSDLTVGGARYISTGRGFATSRIPFSILYSRFAGSAIYMGARSML
KAI8387093.1_F625C VVQELIERGLWKATQRFFRHILSLSPMFEVFAGQIYSSALLSDLTVGGARYISTGRGFATSRIPFSILYSRFAGSAIYMGARSML
KAI8387093.1_F625S VVQELIERGLWKATQRFFRHILSLSPMFEVFAGQIYSSALLSDLTVGGARYISTGRGFATSRIPFSILYSRFAGSAIYMGARSML
KAI8387093.1_I634V VVQELIERGLWKATQRFFRHILSLSPMFEVFAGQIYSSALLSDLTVGGARYISTGRGFATSRIPFSILYSRFAGSAIYMGARSML
KAI8387093.1_S629F VVQELIERGLWKATQRFFRHILSLSPMFEVFAGQIYSSALLSDLTVGGARYISTGRGFATSRIPFSILYSRFAGSAIYMGARSML
KAI8387093.1_S629P VVQELIERGLWKATQRFFRHILSLSPMFEVFAGQIYSSALLSDLTVGGARYISTGRGFATSRIPFSILYSRFAGSAIYMGARSML
KAI8397588.1 VVQELIERGLWKATQRFFRHILSLSPMFEVFAGQIYSSALLSDLTVGGARYISTGRGFATSRIPFSILYSRFAGSAIYMGARSML
1450 1460 1470 1480 1490 1500 1510 1520 1530
KAI8383591.1 MLLFGTVAHWQAPLLWFWASLSALLFSPFIFNPHQFSWEDFFLDYRDYIRWLSRGNNKYHKNSWIGYVRMARSRITGFKRKLIGD
KAI8387093.1 MLLFGTVAHWQAPLLWFWASLSALLFSPFIFNPHQFSWEDFFLDYRDYIRWLSRGNSKYHRNSWIGYVRMARSRITGFKRKLVGD
KAI8387093.1_D632E MLLFGTVAHWQAPLLWFWASLSALLFSPFIFNPHQFSWEDFFLDYRDYIRWLSRGNSKYHRNSWIGYVRMARSRITGFKRKLVGD
KAI8387093.1_D632G MLLFGTVAHWQAPLLWFWASLSALLFSPFIFNPHQFSWEDFFLDYRDYIRWLSRGNSKYHRNSWIGYVRMARSRITGFKRKLVGD
KAI8387093.1_D632H MLLFGTVAHWQAPLLWFWASLSALLFSPFIFNPHQFSWEDFFLDYRDYIRWLSRGNSKYHRNSWIGYVRMARSRITGFKRKLVGD
KAI8387093.1_D632Y MLLFGTVAHWQAPLLWFWASLSALLFSPFIFNPHQFSWEDFFLDYRDYIRWLSRGNSKYHRNSWIGYVRMARSRITGFKRKLVGD
KAI8387093.1_F625C MLLFGTVAHWQAPLLWFWASLSALLFSPFIFNPHQFSWEDFFLDYRDYIRWLSRGNSKYHRNSWIGYVRMARSRITGFKRKLVGD
KAI8387093.1_F625S MLLFGTVAHWQAPLLWFWASLSALLFSPFIFNPHQFSWEDFFLDYRDYIRWLSRGNSKYHRNSWIGYVRMARSRITGFKRKLVGD
KAI8387093.1_I634V MLLFGTVAHWQAPLLWFWASLSALLFSPFIFNPHQFSWEDFFLDYRDYIRWLSRGNSKYHRNSWIGYVRMARSRITGFKRKLVGD
KAI8387093.1_S629F MLLFGTVAHWQAPLLWFWASLSALLFSPFIFNPHQFSWEDFFLDYRDYIRWLSRGNSKYHRNSWIGYVRMARSRITGFKRKLVGD
KAI8387093.1_S629P MLLFGTVAHWQAPLLWFWASLSALLFSPFIFNPHQFSWEDFFLDYRDYIRWLSRGNSKYHRNSWIGYVRMARSRITGFKRKLVGD
KAI8397588.1 MLLFGTVAHWQAPLLWFWASLSALLFSPFIFNPHQFSWEDFFLDYRDYIRWLSRGNSKYHRNSWIGYVRMARSRITGFKRKLVGD

```
                     1531     1540      1550      1560      1570      1580      1590      1600      1610
KAI8383591.1         DSEKAAGDANRAHRTNLILAELIPTAINAGSCFIGFTFINAQTGVKATDDDRVNSVLRVVLCTLGPIAVDVGVLFFCLGMSCCSG
KAI8387093.1         ESEKAAGDASRAHRTNLILAEIIPNAIYAAGCFVGFTFINAQTGVKTTDDDRVNSVLRIIICTLAPIVIDIGVLFFVLGMSCCSG
KAI8387093.1_D632E   ESEKAAGDASRAHRTNLILAEIIPNAIYAAGCFVGFTFINAQTGVKTTDDDRVNSVLRIIICTLAPIVIDIGVLFFVLGMSCCSG
KAI8387093.1_D632G   ESEKAAGDASRAHRTNLILAEIIPNAIYAAGCFVGFTFINAQTGVKTTDDDRVNSVLRIIICTLAPIVIDIGVLFFVLGMSCCSG
KAI8387093.1_D632H   ESEKAAGDASRAHRTNLILAEIIPNAIYAAGCFVGFTFINAQTGVKTTDDDRVNSVLRIIICTLAPIVIDIGVLFFVLGMSCCSG
KAI8387093.1_D632Y   ESEKAAGDASRAHRTNLILAEIIPNAIYAAGCFVGFTFINAQTGVKTTDDDRVNSVLRIIICTLAPIVIDIGVLFFVLGMSCCSG
KAI8387093.1_F625C   ESEKAAGDASRAHRTNLILAEIIPNAIYAAGCFVGFTFINAQTGVKTTDDDRVNSVLRIIICTLAPIVIDIGVLFFVLGMSCCSG
KAI8387093.1_F625S   ESEKAAGDASRAHRTNLILAEIIPNAIYAAGCFVGFTFINAQTGVKTTDDDRVNSVLRIIICTLAPIVIDIGVLFFVLGMSCCSG
KAI8387093.1_I634V   ESEKAAGDASRAHRTNLILAEIIPNAIYAAGCFVGFTFINAQTGVKTTDDDRVNSVLRIIICTLAPIVIDIGVLFFVLGMSCCSG
KAI8387093.1_S629F   ESEKAAGDASRAHRTNLILAEIIPNAIYAAGCFVGFTFINAQTGVKTTDDDRVNSVLRIIICTLAPIVIDIGVLFFVLGMSCCSG
KAI8387093.1_S629P   ESEKAAGDASRAHRTNLILAEIIPNAIYAAGCFVGFTFINAQTGVKTTDDDRVNSVLRIIICTLAPIVIDIGVLFFVLGMSCCSG
KAI8397588.1         ESEKAAGDASRAHRTNLILAEIIPNAIYAAGCFVGFTFINAQTGVKTTDDDRVNSVLRIIICTLAPIVIDIGVLFFVLGMSCCSG

                         1620      1630      1640      1650      1660      1670      1680      1690      1700
KAI8383591.1         PLFGMCCKKTGAVMAAVAHGVSVVIHIGFFIVMWVLEGFNFTRMLVGVATVIQCQRFIFQLMTILLLTREFKNDHANTAFWTGKW
KAI8387093.1         PLFGMCCKRTGSVMAGFAHGIAVIVHIGFFIVMWVLEGFNFTRMLLGVVTMIQCQRLIFQCMTVLMLTREFKNDHANTAFWTGKW
KAI8387093.1_D632E   PLFGMCCKRTGSVMAGFAHGIAVIVHIGFFIVMWVLEGFNFTRMLLGVVTMIQCQRLIFQCMTVLMLTREFKNDHANTAFWTGKW
KAI8387093.1_D632G   PLFGMCCKRTGSVMAGFAHGIAVIVHIGFFIVMWVLEGFNFTRMLLGVVTMIQCQRLIFQCMTVLMLTREFKNDHANTAFWTGKW
KAI8387093.1_D632H   PLFGMCCKRTGSVMAGFAHGIAVIVHIGFFIVMWVLEGFNFTRMLLGVVTMIQCQRLIFQCMTVLMLTREFKNDHANTAFWTGKW
KAI8387093.1_D632Y   PLFGMCCKRTGSVMAGFAHGIAVIVHIGFFIVMWVLEGFNFTRMLLGVVTMIQCQRLIFQCMTVLMLTREFKNDHANTAFWTGKW
KAI8387093.1_F625C   PLFGMCCKRTGSVMAGFAHGIAVIVHIGFFIVMWVLEGFNFTRMLLGVVTMIQCQRLIFQCMTVLMLTREFKNDHANTAFWTGKW
KAI8387093.1_F625S   PLFGMCCKRTGSVMAGFAHGIAVIVHIGFFIVMWVLEGFNFTRMLLGVVTMIQCQRLIFQCMTVLMLTREFKNDHANTAFWTGKW
KAI8387093.1_I634V   PLFGMCCKRTGSVMAGFAHGIAVIVHIGFFIVMWVLEGFNFTRMLLGVVTMIQCQRLIFQCMTVLMLTREFKNDHANTAFWTGKW
KAI8387093.1_S629F   PLFGMCCKRTGSVMAGFAHGIAVIVHIGFFIVMWVLEGFNFTRMLLGVVTMIQCQRLIFQCMTVLMLTREFKNDHANTAFWTGKW
KAI8387093.1_S629P   PLFGMCCKRTGSVMAGFAHGIAVIVHIGFFIVMWVLEGFNFTRMLLGVVTMIQCQRLIFQCMTVLMLTREFKNDHANTAFWTGKW
KAI8397588.1         PLFGMCCKRTGSVMAGFAHGIAVIVHIGFFIVMWVLEGFNFTRMLLGVVTMIQCQRLIFQCMTVLMLTREFKNDHANTAFWTGKW

                     1701     1710      1720      1730      1740      1750      1760      1770      1780
KAI8383591.1         YGSGFGYMAWTQPMRELTAKVIEMSEFAADFVLGHVILFAQFPVLCIPAIDKFHSIMLFWLKPSRHIRPPIYSLKQSRLRKRMVK
KAI8387093.1         YGSGFGYMAWTQPTRELTAKVIELSEFAADFVLGHVILFAQFPVLCIPAIDKFHSIMLFWLKPSRQIRPPIYSLKQSRLRKRMVK
KAI8387093.1_D632E   YGSGFGYMAWTQPTRELTAKVIELSEFAADFVLGHVILFAQFPVLCIPAIDKFHSIMLFWLKPSRQIRPPIYSLKQSRLRKRMVK
KAI8387093.1_D632G   YGSGFGYMAWTQPTRELTAKVIELSEFAADFVLGHVILFAQFPVLCIPAIDKFHSIMLFWLKPSRQIRPPIYSLKQSRLRKRMVK
KAI8387093.1_D632H   YGSGFGYMAWTQPTRELTAKVIELSEFAADFVLGHVILFAQFPVLCIPAIDKFHSIMLFWLKPSRQIRPPIYSLKQSRLRKRMVK
KAI8387093.1_D632Y   YGSGFGYMAWTQPTRELTAKVIELSEFAADFVLGHVILFAQFPVLCIPAIDKFHSIMLFWLKPSRQIRPPIYSLKQSRLRKRMVK
KAI8387093.1_F625C   YGSGFGYMAWTQPTRELTAKVIELSEFAADFVLGHVILFAQFPVLCIPAIDKFHSIMLFWLKPSRQIRPPIYSLKQSRLRKRMVK
KAI8387093.1_F625S   YGSGFGYMAWTQPTRELTAKVIELSEFAADFVLGHVILFAQFPVLCIPAIDKFHSIMLFWLKPSRQIRPPIYSLKQSRLRKRMVK
KAI8387093.1_I634V   YGSGFGYMAWTQPTRELTAKVIELSEFAADFVLGHVILFAQFPVLCIPAIDKFHSIMLFWLKPSRQIRPPIYSLKQSRLRKRMVK
KAI8387093.1_S629F   YGSGFGYMAWTQPTRELTAKVIELSEFAADFVLGHVILFAQFPVLCIPAIDKFHSIMLFWLKPSRQIRPPIYSLKQSRLRKRMVK
KAI8387093.1_S629P   YGSGFGYMAWTQPTRELTAKVIELSEFAADFVLGHVILFAQFPVLCIPAIDKFHSIMLFWLKPSRQIRPPIYSLKQSRLRKRMVK
KAI8397588.1         YGSGFGYMAWTQPTRELTAKVIELSEFAADFVLGHVILFAQFPVLCIPAIDKFHSIMLFWLKPSRQIRPPIYSLKQSRLRKRMVK
```

	1790 1800 1810 1820 1830 1840 1850 1860 1870
KAI8383591.1	RYLTLYIIIFLVFAGAIVGPAVAASHVPQDIGHTLTGPFHNIVQPRNKSNN
KAI8387093.1	RYLTLYIIVFLVFAGCIVGPAVASSHVAKDLGHQLTGTFHNLVQPRNVSNNDTGFGISTYSNHYYTHTPSLKTWSTIK
KAI8387093.1_D632E	RYLTLYIIVFLVFAGCIVGPAVASSHVAKDLGHQLTGTFHNLVQPRNVSNNDTGFGISTYSNHYYTHTPSLKTWSTIK
KAI8387093.1_D632G	RYLTLYIIVFLVFAGCIVGPAVASSHVAKDLGHQLTGTFHNLVQPRNVSNNDTGFGISTYSNHYYTHTPSLKTWSTIK
KAI8387093.1_D632H	RYLTLYIIVFLVFAGCIVGPAVASSHVAKDLGHQLTGTFHNLVQPRNVSNNDTGFGISTYSNHYYTHTPSLKTWSTIK
KAI8387093.1_D632Y	RYLTLYIIVFLVFAGCIVGPAVASSHVAKDLGHQLTGTFHNLVQPRNVSNNDTGFGISTYSNHYYTHTPSLKTWSTIK
KAI8387093.1_F625C	RYLTLYIIVFLVFAGCIVGPAVASSHVAKDLGHQLTGTFHNLVQPRNVSNNDTGFGISTYSNHYYTHTPSLKTWSTIK
KAI8387093.1_F625S	RYLTLYIIVFLVFAGCIVGPAVASSHVAKDLGHQLTGTFHNLVQPRNVSNNDTGFGISTYSNHYYTHTPSLKTWSTIK
KAI8387093.1_I634V	RYLTLYIIVFLVFAGCIVGPAVASSHVAKDLGHQLTGTFHNLVQPRNVSNNDTGFGISTYSNHYYTHTPSLKTWSTIK
KAI8387093.1_S629F	RYLTLYIIVFLVFAGCIVGPAVASSHVAKDLGHQLTGTFHNLVQPRNVSNNDTGFGISTYSNHYYTHTPSLKTWSTIK
KAI8387093.1_S629P	RYLTLYIIVFLVFAGCIVGPAVASSHVAKDLGHQLTGTFHNLVQPRNVSNNDTGFGISTYSNHYYTHTPSLKTWSTIK
KAI8397588.1	RYLTLYIIVFLVFAGCIVGPAVASSHVAKDLGHQLTGTFHNLVQPRNVSNNDTGFGISTYSNHYYTHTPSLKTWSTIK

Table S5. SSSCPreds data of FKS1 for *Saccharomyces cerevisiae* with SSSC data of PDB structures.

	1 10 20 30 40 50 60 70 80
CAF1573557.1	MSYNDPNLNGQYYSNGDGT
	GDGNYPTYQVTQDQSAYDEYGQPIYTQNQLDDGYYDPNEQYVDGTQFPQGQDPSQDQGPYNNDASYCNQPPNMMNPSSQDGENFS
KAJ1045660.1	MNTDQQPYQGQTDYTQGPDNGQSQEQDYDQYGQPLYPSQADGYYDPNVAAGTEADMYGQQPPNESYDQDYTNGEYYGQPPNMAAQ
KAJ1538062.1	MNTDQQPYQGQTDYTQGPGNGQSQEQDYDQYGQPLYPSQADGYYDPNVAAGTEADMYGQQPPNESYDQDYTNGEYYGQPPNMAAQ
KAJ1538062.1_S643P	MNTDQQPYQGQTDYTQGPGNGQSQEQDYDQYGQPLYPSQADGYYDPNVAAGTEADMYGQQPPNESYDQDYTNGEYYGQPPNMAAQ

	90 100 110 120 130 140 150 160 170
CAF1573557.1	DFSSYGPPSGTYPNDQYTPSQMSYPDQDGSSGASTPYGNGVVNGNGQYYDPNAIEMALPNDPYPAWTADPQSPLPIEQIEDIFID
KAJ1045660.1	DGENFSDFSSYGPPGTPGYDSYGGQYTPSQMSYGEPNSSGTSTPIYGNYDPNAIAMALPNEPYPAWTADSQSPVSIEQIEDIFID
KAJ1538062.1	DGENFSDFSSYGPPGTPGYDSYGGQYTASQMSYGEPNSSGTSTPIYGNYDPNAIAMALPNEPYPAWTADSQSPVSIEQIEDIFID
KAJ1538062.1_S643P	DGENFSDFSSYGPPGTPGYDSYGGQYTASQMSYGEPNSSGTSTPIYGNYDPNAIAMALPNEPYPAWTADSQSPVSIEQIEDIFID
7ex4_F	EPYPAWTADSQSPVSIEQIEDIFID
7ex4_F	TSSTHHHHSHTSHSSHHHHHHHHHH
7yuy_F	EPYPAWTADSQSPVSIEQIEDIFID
7yuy_F	TSSHHHHTSHHSHSSHHHHHHHHHH

	171 180 190 200 210 220 230 240 250
CAF1573557.1	LTNKFGFQRDSMRNMFDHFMTLLDSRSSRMSPEQALLSLHADYIGGDTANYKKWYFAAQLDMDDEIGFRNMKLGKLSRKARKAKK
KAJ1045660.1	LTNRLGFQRDSMRNMFDHFMVLLDSRSSRMSPDQALLSLHADYIGGDTANYKKWYFAAQLDMDDEVGFRNMSLGKLSRKARKAKK
KAJ1538062.1	LTNRLGFQRDSMRNMFDHFMVLLDSRSSRMSPDQALLSLHADYIGGDTANYKKWYFAAQLDMDDEIGFRNMSLGKLSRKARKAKK
KAJ1538062.1_S643P	LTNRLGFQRDSMRNMFDHFMVLLDSRSSRMSPDQALLSLHADYIGGDTANYKKWYFAAQLDMDDEIGFRNMSLGKLSRKARKAKK
7ex4_F	LTNRLGFQRDSMRNMFDHFMVLLDSRSSRMSPDQALLSLHADYIGGDTANYKKWYFAAQLDMDDEIGFRNMSLX
7ex4_F	HHHHHTSSHHHHHHHHHHHHHHHHHHHHHSSHHHHHHHHHHHHSTSHHSHHHHHHHHHHTTTHHHHHHHHHHTD
7yuy_F	LTNRLGFQRDSMRNMFDHFMVLLDSRSSRMSPDQALLSLHADYIGGDTANYKKWYFAAQLDMDDEIGFRNMSLX
7yuy_F	HHHHHTSSHHHHHHHHHHHHHHHHHHHHHSSHHHHHHHHHHHHSTSHHSHHHHHHHHHHHTTHHHHHHHHHHTD

	260 270 280 290 300 310 320 330 340
CAF1573557.1	KNKKAMQEASPEDTEETLNQIEGDNSLEAADFRWKSKMNQLSPFEMVRQIALFLLCWGEANQVRFTPECLCFIYKCASDYLDSAQ
KAJ1045660.1	KNKKAMEEANPEDTEETLNKIEGDNSLEAADFRWKAKMNQLSPLERVRHIALYLLCWGEANQVRFTAECLCFIYKCALDYLDSPL
KAJ1538062.1	KNKKAMEEANPEDTEETLNKIEGDNSLEAADFRWKAKMNQLSPLERVRHIALYLLCWGEANQVRFTAECLCFIYKCALDYLDSPL
KAJ1538062.1_S643P	KNKKAMEEANPEDTEETLNKIEGDNSLEAADFRWKAKMNQLSPLERVRHIALYLLCWGEANQVRFTAECLCFIYKCALDYLDSPL
7ex4_F	DNSLEAADFRWKAKMNQLSPLERVRHIALYLLCWGEANQVRFTAECLCFIYKCALDYLDSPL
7ex4_F	TTTHHHHHHHHHHHHHHSSHHHHHHHHHHHHHHHHHSSTHHHSHHHHHHHHHHHHHHHHSHH
7yuy_F	DNSLEAADFRWKAKMNQLSPLERVRHIALYLLCWGEANQVRFTAECLCFIYKCALDYLDSPL
7yuy_F	TTTHHHHHHHHHHHHHTSSHHHHHHHHHHHHHHHHHTSTHHHSHHHHHHHHHHHHHHHHSHH

	341 350 360 370 380 390 400 410 420
CAF1573557.1	CQQRPDPLPEGDFLNRVITPLYCFIRSQVYEIVDGRYVKSEKDHNKVIGYDDVNQLFWYPEGIAKIVMEDGTRLIDLPAEERYLK
KAJ1045660.1	CQQRQEPMPEGDFLNRVITPIYHFIRNQVYEIIDGRFVKRERDHNKIVGYDDLNQLFWYPEGIAKIVLEDGTKLIELPLEERYLR
KAJ1538062.1	CQQRQEPMPEGDFLNRVITPIYHFIRNQVYEIVDGRFVKRERDHNKIVGYDDLNQLFWYPEGIAKIVLEDGTKLIELPLEERYLR
KAJ1538062.1_S643P	CQQRQEPMPEGDFLNRVITPIYHFIRNQVYEIVDGRFVKRERDHNKIVGYDDLNQLFWYPEGIAKIVLEDGTKLIELPLEERYLR
7ex4_F	CQQRQEPMPEGDFLNRVITPIYHFIRNQVYEIVDGRFVKRERDHNKIVGYDDLNQLFWYPEGIAKIVLEDGTKLIELPLEERYLR
7ex4_F	HHHSTSSSSSTHHHHHHHHHHHHHHHHHSTSSSTTSSSSSSSSHHTSSSHHHHHHHHHSHHHHHHSSSHHTSSHHHSSHHHHHHH
7yuy_F	CQQRQEPMPEGDFLNRVITPIYHFIRNQVYEIVDGRFVKRERDHNKIVGYDDLNQLFWYPEGIAKIVLEDGTKLIELPLEERYLR
7yuy_F	HHHSHSSSSSTHHHHHHHHHHHHHHHHHSTSSSTTSSSSSSSSTHTSSSHHHHHHHHHSHHHHHHSSSHHTSSHHHSSHHTHHHH

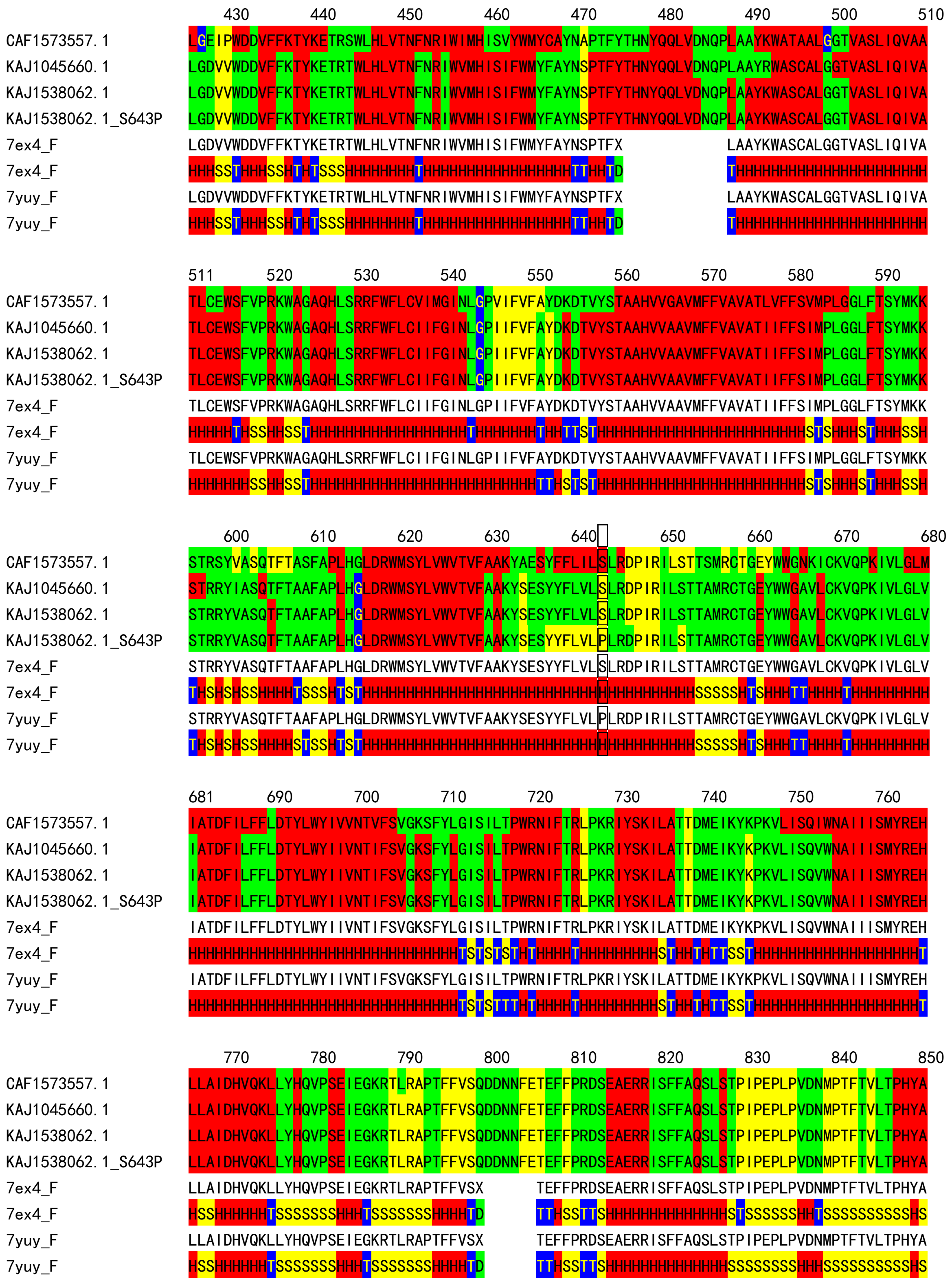
430 440 450 460 470 480 490 500 510
CAF1573557.1 LGEIPWDDVFFKTYKETRSWLHLVTNFNRIWIMHISVYWMYCAYNAPTFYTHNYQQLVDNQPLAAYKWATAALGGTVASLIQVAA
KAJ1045660.1 LGDVVWDDVFFKTYKETRTWLHLVTNFNRIWVMHISIFWMYFAYNSPTFYTHNYQQLVDNQPLAAYRWASCALGGTVASLIQIVA
KAJ1538062.1 LGDVVWDDVFFKTYKETRTWLHLVTNFNRIWVMHISIFWMYFAYNSPTFYTHNYQQLVDNQPLAAYKWASCALGGTVASLIQIVA
KAJ1538062.1_S643P LGDVVWDDVFFKTYKETRTWLHLVTNFNRIWVMHISIFWMYFAYNSPTFYTHNYQQLVDNQPLAAYKWASCALGGTVASLIQIVA
7ex4_F LGDVVWDDVFFKTYKETRTWLHLVTNFNRIWVMHISIFWMYFAYNSPTFX LAAYKWASCALGGTVASLIQIVA
7ex4_F HHHSSTHHHSSHTHTSSSHHHHHHHHTHHHHHHHHHHHHHHHHHTTHHTD THHHHHHHHHHHHHHHHHHHHHH
7yuy_F LGDVVWDDVFFKTYKETRTWLHLVTNFNRIWVMHISIFWMYFAYNSPTFX LAAYKWASCALGGTVASLIQIVA
7yuy_F HHHSSTHHHSSHTHTSSSHHHHHHHHTHHHHHHHHHHHHHHHHHTTHHTD THHHHHHHHHHHHHHHHHHHHHH
511 520 530 540 550 560 570 580 590
CAF1573557.1 TLCEWSFVPRKWAGAQHLSRRFWFLCVIMGINLGPVIFVFAYDKDTVYSTAAHVVGAVMFFVAVATLVFFSVMPLGGLFTSYMKK
KAJ1045660.1 TLCEWSFVPRKWAGAQHLSRRFWFLCIIFGINLGPIIFVFAYDKDTVYSTAAHVVAAVMFFVAVATIIFFSIMPLGGLFTSYMKK
KAJ1538062.1 TLCEWSFVPRKWAGAQHLSRRFWFLCIIFGINLGPIIFVFAYDKDTVYSTAAHVVAAVMFFVAVATIIFFSIMPLGGLFTSYMKK
KAJ1538062.1_S643P TLCEWSFVPRKWAGAQHLSRRFWFLCIIFGINLGPIIFVFAYDKDTVYSTAAHVVAAVMFFVAVATIIFFSIMPLGGLFTSYMKK
7ex4_F TLCEWSFVPRKWAGAQHLSRRFWFLCIIFGINLGPIIFVFAYDKDTVYSTAAHVVAAVMFFVAVATIIFFSIMPLGGLFTSYMKK
7ex4_F HHHHHTHSSHHSSTHHHHHHHHHHHHHHHHHHTHHHHHHTHHTTSTHHHHHHHHHHHHHHHHHHHHHHHHHHSTSHHHSTHHHSSH
7yuy_F TLCEWSFVPRKWAGAQHLSRRFWFLCIIFGINLGPIIFVFAYDKDTVYSTAAHVVAAVMFFVAVATIIFFSIMPLGGLFTSYMKK
7yuy_F HHHHHHHSSHHSSTHHHHHHHHHHHHHHHHHHHHHHHHHHTTHSTSTHHHHHHHHHHHHHHHHHHHHHHHHHSTSHHHSTHHHSSH
600 610 620 630 640 650 660 670 680
CAF1573557.1 STRSYVASQTFTASFAPLHGLDRWMSYLVWVTVFAAKYAESYFFLILSLRDPIRILSTTSMRCTGEYWWGNKICKVQPKIVLGLM
KAJ1045660.1 STRRYIASQTFTAAFAPLHGLDRWMSYLVWVTVFAAKYSESYYFLVLSLRDPIRILSTTAMRCTGEYWWGAVLCKVQPKIVLGLV
KAJ1538062.1 STRRYVASQTFTAAFAPLHGLDRWMSYLVWVTVFAAKYSESYYFLVLSLRDPIRILSTTAMRCTGEYWWGAVLCKVQPKIVLGLV
KAJ1538062.1_S643P STRRYVASQTFTAAFAPLHGLDRWMSYLVWVTVFAAKYSESYYFLVLPLRDPIRILSTTAMRCTGEYWWGAVLCKVQPKIVLGLV
7ex4_F STRRYVASQTFTAAFAPLHGLDRWMSYLVWVTVFAAKYSESYYFLVLSLRDPIRILSTTAMRCTGEYWWGAVLCKVQPKIVLGLV
7ex4_F THSHSHSSHHHHTSSSHTSTHHHHHHHHHHHHHHHHHHHHHHHHHHHHHHHHHHHHHHHHHHSSSSSHTSHHHTTHHHHTHHHHHHHHH
7yuy_F STRRYVASQTFTAAFAPLHGLDRWMSYLVWVTVFAAKYSESYYFLVLPLRDPIRILSTTAMRCTGEYWWGAVLCKVQPKIVLGLV
7yuy_F THSHSHSSHHHHSTSSHTSTHHHHHHHHHHHHHHHHHHHHHHHHHHHHHHHHHHHHHHHHHHSSSSSHTSHHHTTHHHHTHHHHHHHHH
681 690 700 710 720 730 740 750 760
CAF1573557.1 IATDFILFFLDTYLWYIVVNTVFSVGKSFYLGISILTPWRNIFTRLPKRIYSKILATTDMEIKYKPKVLISQIWNAIIISMYREH
KAJ1045660.1 IATDFILFFLDTYLWYIIVNTIFSVGKSFYLGISILTPWRNIFTRLPKRIYSKILATTDMEIKYKPKVLISQVWNAIIISMYREH
KAJ1538062.1 IATDFILFFLDTYLWYIIVNTIFSVGKSFYLGISILTPWRNIFTRLPKRIYSKILATTDMEIKYKPKVLISQVWNAIIISMYREH
KAJ1538062.1_S643P IATDFILFFLDTYLWYIIVNTIFSVGKSFYLGISILTPWRNIFTRLPKRIYSKILATTDMEIKYKPKVLISQVWNAIIISMYREH
7ex4_F IATDFILFFLDTYLWYIIVNTIFSVGKSFYLGISILTPWRNIFTRLPKRIYSKILATTDMEIKYKPKVLISQVWNAIIISMYREH
7ex4_F HHHHHHHHHHHHHHHHHHHHHHHHHHHHHHHTSTSTSTHTHHHHTHHHHHHHHHSTHHTHTTSSTHHHHHHHHHHHHHHHHHHHT
7yuy_F IATDFILFFLDTYLWYIIVNTIFSVGKSFYLGISILTPWRNIFTRLPKRIYSKILATTDMEIKYKPKVLISQVWNAIIISMYREH
7yuy_F HHHHHHHHHHHHHHHHHHHHHHHHHHHHHHHTSTSTTTHTHHHHTHHHHHHHHHSTHHTHTTSSTHHHHHHHHHHHHHHHHHHHT
770 780 790 800 810 820 830 840 850
CAF1573557.1 LLAIDHVQKLLYHQVPSEIEGKRTLRAPTFFVSQDDNNFETEFFPRDSEAERRISFFAQSLSTPIPEPLPVDNMPTFTVLTPHYA
KAJ1045660.1 LLAIDHVQKLLYHQVPSEIEGKRTLRAPTFFVSQDDNNFETEFFPRDSEAERRISFFAQSLSTPIPEPLPVDNMPTFTVLTPHYA
KAJ1538062.1 LLAIDHVQKLLYHQVPSEIEGKRTLRAPTFFVSQDDNNFETEFFPRDSEAERRISFFAQSLSTPIPEPLPVDNMPTFTVLTPHYA
KAJ1538062.1_S643P LLAIDHVQKLLYHQVPSEIEGKRTLRAPTFFVSQDDNNFETEFFPRDSEAERRISFFAQSLSTPIPEPLPVDNMPTFTVLTPHYA
7ex4_F LLAIDHVQKLLYHQVPSEIEGKRTLRAPTFFVSX TEFFPRDSEAERRISFFAQSLSTPIPEPLPVDNMPTFTVLTPHYA
7ex4_F HSSHHHHHHTSSSSSSSHHHTSSSSSSSHHHHTD TTHSSTTSHHHHHHHHHHHHHHSTSSSSSSHHTSSSSSSSSSSHS
7yuy_F LLAIDHVQKLLYHQVPSEIEGKRTLRAPTFFVSX TEFFPRDSEAERRISFFAQSLSTPIPEPLPVDNMPTFTVLTPHYA
7yuy_F HSSHHHHHHTSSSSSSSHHHTSSSSSSSHHHHTD TTHSSTTSHHHHHHHHHHHHHHSSSSSSSSHHHSSSSSSSSSSHS

```
                     851       860       870       880       890       900       910       920       930
CAF1573557.1         ERILLSLREIIREDDQFSRVTLLEYLKQLHPVEWDCFVKDTKILAEETAAYENNEDEPEKEDALKSQIDDLPFYCIGFKSAAPEY
KAJ1045660.1         ERILLSLREIIREDDQFSRVTLLEYLKQLHPVEWECFVKDTKILAEETAAYEGNENEAEKEDALKSQIDDLPFYCIGFKSAAPEY
KAJ1538062.1         ERILLSLREIIREDDQFSRVTLLEYLKQLHPVEWECFVKDTKILAEETAAYEGNENEAEKEDALKSQIDDLPFYCIGFKSAAPEY
KAJ1538062.1_S643P   ERILLSLREIIREDDQFSRVTLLEYLKQLHPVEWECFVKDTKILAEETAAYEGNENEAEKEDALKSQIDDLPFYCIGFKSAAPEY
7ex4_F               ERILLSLREIIREDDQFSRVTLLEYLKQLHPVEWECFVKDTKILAEX                                 APEY
7ex4_F               SSHSHTHHTHHTSHTHSSTSSHHHHHHHHTTHHHHHHHHHHHHHHTD                                 THHH
7yuy_F               ERILLSLREIIREDDQFSRVTLLEYLKQLHPVEWECFVKDTKILAEX                                 APEY
7yuy_F               SSHSHTHHHHHTSHTHSSTSSHHHHHHHHTTHHHHHHHHHHHHHHTD                                 THHH

                         940       950       960       970       980       990       1000      1010      1020
CAF1573557.1         TLRTRIWASLRSQTLYRTISGFMNYSRAIKLLYRVENPEIVQMFGGNADGLERELEKMARRKFKFLVSMQRLAKFKPHELENAEF
KAJ1045660.1         TLRTRIWASLRSQTLYRTISGFMNYSRAIKLLYRVENPEIVQMFGGNAEGLERELEKMARRKFKFLVSMQRLAKFKPHELENAEF
KAJ1538062.1         TLRTRIWASLRSQTLYRTISGFMNYSRAIKLLYRVENPEIVQMFGGNAEGLERELEKMARRKFKFLVSMQRLAKFKPHELENAEF
KAJ1538062.1_S643P   TLRTRIWASLRSQTLYRTISGFMNYSRAIKLLYRVENPEIVQMFGGNAEGLERELEKMARRKFKFLVSMQRLAKFKPHELENAEF
7ex4_F               TLRTRIWASLRSQTLYRTISGFMNYSRAIKLLYRVENPEIVQMFGGNAEGLERELEKMARRKFKFLVSMQRLAKFKPHELENAEF
7ex4_F               HHHHHHHHHHSTSSHHHHHHHHHHHHHHHHHHHHHHTHHHHHHTSTTHHHHHHHHHHHHHHHSSSSSTSSTHHHSSHHHHHHHHH
7yuy_F               TLRTRIWASLRSQTLYRTISGFMNYSRAIKLLYRVENPEIVQMFGGNAEGLERELEKMARRKFKFLVSMQRLAKFKPHELENAEF
7yuy_F               HHHHHHHHHHSTSSHHHHHHHHHHHHHHHHHHHHHHSHHHHHHTSTTHHHHHHHHHHHHHHHSSSSSTSSTHHTSSHHHHHHHHH

                         1110      1120      1130      1140      1150      1160      1170      1180      1190
CAF1573557.1         LLRAYPDLQIAYLDEEPPLSEGEEPRIYSALIDGHCEILENGRRRPKFRVQLSGNPILGDGKSDNQNHALIFYRGEYIQLIDANQ
KAJ1045660.1         LLRAYPDLQIAYLDEEPPLTEGEEPRIYSALIDGHCEILDNGRRRPKFRVQLSGNPILGDGKSDNQNHALIFYRGEYIQLIDANQ
KAJ1538062.1         LLRAYPDLQIAYLDEEPPLTEGEEPRIYSALIDGHCEILDNGRRRPKFRVQLSGNPILGDGKSDNQNHALIFYRGEYIQLIDANQ
KAJ1538062.1_S643P   LLRAYPDLQIAYLDEEPPLTEGEEPRIYSALIDGHCEILDNGRRRPKFRVQLSGNPILGDGKSDNQNHALIFYRGEYIQLIDANQ
7ex4_F               LLRAYPDLQIAYLDEEPPLTEGEEPRIYSALIDGHCEILDNGRRRPKFRVQLSGNPILGDGKSDNQNHALIFYRGEYIQLIDANQ
7ex4_F               HHHHSHTSSSSSSSSSSSHSSTSSSSSSSSSSSHHSSSSHHTSSSSHSSSSSHTSHSHSHSTHHHHHHHHHTHSTHSSSSSSHSS
7yuy_F               LLRAYPDLQIAYLDEEPPLTEGEEPRIYSALIDGHCEILDNGRRRPKFRVQLSGNPILGDGKSDNQNHALIFYRGEYIQLIDANQ
7yuy_F               HHHHSTHSSSSSSSSSSSHSSTSSSSSSSSSSSHHSSSSHHTSSSSHSSSSSHTSTSHSHSHHHHHHHHHHTHSTHSSSSSSHSS

                     1191      1200      1210      1220      1230      1240      1250      1260      1270
CAF1573557.1         DNYLEECLKIRSVLAEFEELGIEQIHPYTPGLKYEDQSTNHPVAIVGAREYIFSENSGVLGDVAAGKEQTFGTLFARTLAQIGGK
KAJ1045660.1         DNYLEECLKIRSVLAEFEELNVEQVNPYAPGLRYEEQTTNHPVAIVGAREYIFSENSGVLGDVAAGKEQTFGTLFARTLSQIGGK
KAJ1538062.1         DNYLEECLKIRSVLAEFEELNVEQVNPYAPGLRYEEQTTNHPVAIVGAREYIFSENSGVLGDVAAGKEQTFGTLFARTLSQIGGK
KAJ1538062.1_S643P   DNYLEECLKIRSVLAEFEELNVEQVNPYAPGLRYEEQTTNHPVAIVGAREYIFSENSGVLGDVAAGKEQTFGTLFARTLSQIGGK
7ex4_F               DNYLEECLKIRSVLAEFEELNVEQVNPYAPGLRYEEQTTNHPVAIVGAREYIFX        VAAGKEQTFGTLFARTLSQIGGK
7ex4_F               HTSHHHHHTHHHHHHHSTHHTSSSHTHSSSTTSTHHSHTHSSSHSSSSSHTSTD        THHHHHHHHHHHHHHHHHHHHHH
7yuy_F               DNYLEECLKIRSVLAEFEELNVEQVNPYAPGLRYEEQTTNHPVAIVGAREYIFX        VAAGKEQTFGTLFARTLSQIGGK
7yuy_F               HTSHHHHHTHHHHHHHTTHHTSSSHTHSSSTTSTHHSHTHTSSHSSSSSHSSTD        THHHHHHHHHHHHHHHHHHHHHH

                         1280      1290      1300      1310      1320      1330      1340      1350      1360
CAF1573557.1         LHYGHPDFINATFMTTRGGVSKAQKGLHLNEDIYAGMNAVLRGGRIKHCEYYQCGKGRDLGFGTILNFTTKIGAGMGEQMLSREY
KAJ1045660.1         LHYGHPDFINATFMTTRGGVSKAQKGLHLNEDIYAGMNAMLRGGRIKHCEYYQCGKGRDLGFGTILNFTTKIGAGMGEQMLSREY
KAJ1538062.1         LHYGHPDFINATFMTTRGGVSKAQKGLHLNEDIYAGMNAMLRGGRIKHCEYYQCGKGRDLGFGTILNFTTKIGAGMGEQMLSREY
KAJ1538062.1_S643P   LHYGHPDFINATFMTTRGGVSKAQKGLHLNEDIYAGMNAMLRGGRIKHCEYYQCGKGRDLGFGTILNFTTKIGAGMGEQMLSREY
7ex4_F               LHYGHPDFINATFMTTRGGVSKAQKGLHLNEDIYAGMNAMLRGGRIKHCEYYQCGKX                   GEQMLSREY
7ex4_F               HTHHSSHSSSHHHHHHTTSHSSSSHHSSHTTHHHHHHHHHHHTSSSSSSHTSSHTTD                   THHHHSHHH
7yuy_F               LHYGHPDFINATFMTTRGGVSKAQKGLHLNEDIYAGMNAMLRGGRIKHCEYYQCGKX                   GEQMLSREY
7yuy_F               HTHHSSHSSSHHHHHHSTSHSSSSHHSSHTTHHHHHHHHHHHTSSSSSSHTSSHTTD                   THHHHSHHH
```

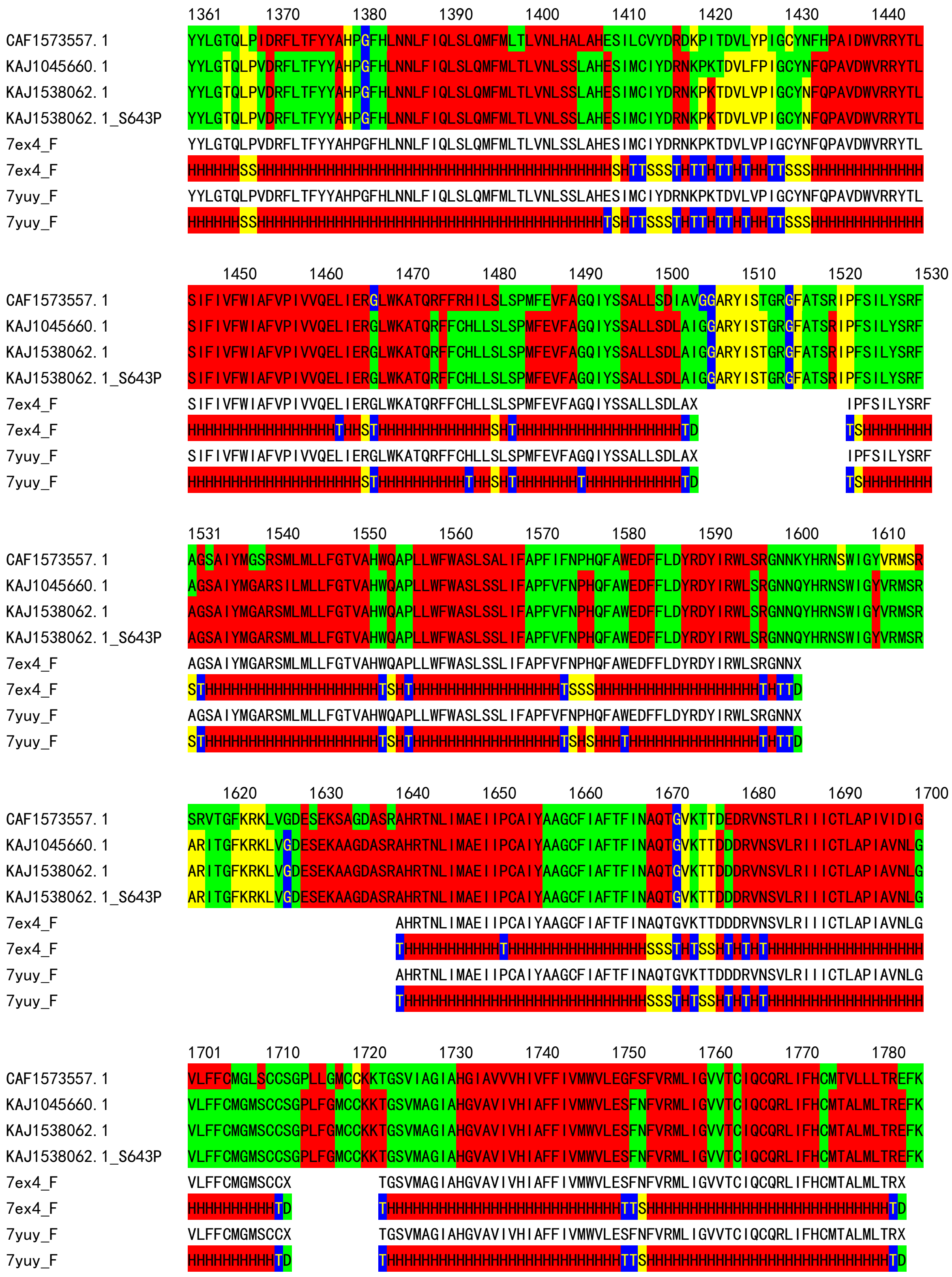

1361 1370 1380 1390 1400 1410 1420 1430 1440
CAF1573557.1 YYLGTQLPIDRFLTFYYAHPGFHLNNLFIQLSLQMFMLTLVNLHALAHESILCVYDRDKPITDVLYPIGCYNFHPAIDWVRRYTL
KAJ1045660.1 YYLGTQLPVDRFLTFYYAHPGFHLNNLFIQLSLQMFMLTLVNLSSLAHESIMCIYDRNKPKTDVLFPIGCYNFQPAVDWVRRYTL
KAJ1538062.1 YYLGTQLPVDRFLTFYYAHPGFHLNNLFIQLSLQMFMLTLVNLSSLAHESIMCIYDRNKPKTDVLVPIGCYNFQPAVDWVRRYTL
KAJ1538062.1_S643P YYLGTQLPVDRFLTFYYAHPGFHLNNLFIQLSLQMFMLTLVNLSSLAHESIMCIYDRNKPKTDVLVPIGCYNFQPAVDWVRRYTL
7ex4_F YYLGTQLPVDRFLTFYYAHPGFHLNNLFIQLSLQMFMLTLVNLSSLAHESIMCIYDRNKPKTDVLVPIGCYNFQPAVDWVRRYTL
7ex4_F
7yuy_F YYLGTQLPVDRFLTFYYAHPGFHLNNLFIQLSLQMFMLTLVNLSSLAHESIMCIYDRNKPKTDVLVPIGCYNFQPAVDWVRRYTL
7yuy_F
1450 1460 1470 1480 1490 1500 1510 1520 1530
CAF1573557.1 SIFIVFWIAFVPIVVQELIERGLWKATQRFFRHILSLSPMFEVFAGQIYSSALLSDIAVGGARYISTGRGFATSRIPFSILYSRF
KAJ1045660.1 SIFIVFWIAFVPIVVQELIERGLWKATQRFFCHLLSLSPMFEVFAGQIYSSALLSDLAIGGARYISTGRGFATSRIPFSILYSRF
KAJ1538062.1 SIFIVFWIAFVPIVVQELIERGLWKATQRFFCHLLSLSPMFEVFAGQIYSSALLSDLAIGGARYISTGRGFATSRIPFSILYSRF
KAJ1538062.1_S643P SIFIVFWIAFVPIVVQELIERGLWKATQRFFCHLLSLSPMFEVFAGQIYSSALLSDLAIGGARYISTGRGFATSRIPFSILYSRF
7ex4_F SIFIVFWIAFVPIVVQELIERGLWKATQRFFCHLLSLSPMFEVFAGQIYSSALLSDLAX IPFSILYSRF
7ex4_F
7yuy_F SIFIVFWIAFVPIVVQELIERGLWKATQRFFCHLLSLSPMFEVFAGQIYSSALLSDLAX IPFSILYSRF
7yuy_F
1531 1540 1550 1560 1570 1580 1590 1600 1610
CAF1573557.1 AGSAIYMGSRSMLMLLFGTVAHWQAPLLWFWASLSALIFAPFIFNPHQFAWEDFFLDYRDYIRWLSRGNNKYHRNSWIGYVRMSR
KAJ1045660.1 AGSAIYMGARSILMLLFGTVAHWQAPLLWFWASLSSLIFAPFVFNPHQFAWEDFFLDYRDYIRWLSRGNNQYHRNSWIGYVRMSR
KAJ1538062.1 AGSAIYMGARSMLMLLFGTVAHWQAPLLWFWASLSSLIFAPFVFNPHQFAWEDFFLDYRDYIRWLSRGNNQYHRNSWIGYVRMSR
KAJ1538062.1_S643P AGSAIYMGARSMLMLLFGTVAHWQAPLLWFWASLSSLIFAPFVFNPHQFAWEDFFLDYRDYIRWLSRGNNQYHRNSWIGYVRMSR
7ex4_F AGSAIYMGARSMLMLLFGTVAHWQAPLLWFWASLSSLIFAPFVFNPHQFAWEDFFLDYRDYIRWLSRGNNX
7ex4_F
7yuy_F AGSAIYMGARSMLMLLFGTVAHWQAPLLWFWASLSSLIFAPFVFNPHQFAWEDFFLDYRDYIRWLSRGNNX
7yuy_F
1620 1630 1640 1650 1660 1670 1680 1690 1700
CAF1573557.1 SRVTGFKRKLVGDESEKSAGDASRAHRTNLIMAEIIPCAIYAAGCFIAFTFINAQTGVKTTDEDRVNSTLRIIICTLAPIVIDIG
KAJ1045660.1 ARITGFKRKLVGDESEKAAGDASRAHRTNLIMAEIIPCAIYAAGCFIAFTFINAQTGVKTTDDDRVNSVLRIIICTLAPIAVNLG
KAJ1538062.1 ARITGFKRKLVGDESEKAAGDASRAHRTNLIMAEIIPCAIYAAGCFIAFTFINAQTGVKTTDDDRVNSVLRIIICTLAPIAVNLG
KAJ1538062.1_S643P ARITGFKRKLVGDESEKAAGDASRAHRTNLIMAEIIPCAIYAAGCFIAFTFINAQTGVKTTDDDRVNSVLRIIICTLAPIAVNLG
7ex4_F AHRTNLIMAEIIPCAIYAAGCFIAFTFINAQTGVKTTDDDRVNSVLRIIICTLAPIAVNLG
7ex4_F
7yuy_F AHRTNLIMAEIIPCAIYAAGCFIAFTFINAQTGVKTTDDDRVNSVLRIIICTLAPIAVNLG
7yuy_F
1701 1710 1720 1730 1740 1750 1760 1770 1780
CAF1573557.1 VLFFCMGLSCCSGPLLGMCCKKTGSVIAGIAHGIAVVVHIVFFIVMWVLEGFSFVRMLIGVVTCIQCQRLIFHCMTVLLLTREFK
KAJ1045660.1 VLFFCMGMSCCSGPLFGMCCKKTGSVMAGIAHGVAVIVHIAFFIVMWVLESFNFVRMLIGVVTCIQCQRLIFHCMTALMLTREFK
KAJ1538062.1 VLFFCMGMSCCSGPLFGMCCKKTGSVMAGIAHGVAVIVHIAFFIVMWVLESFNFVRMLIGVVTCIQCQRLIFHCMTALMLTREFK
KAJ1538062.1_S643P VLFFCMGMSCCSGPLFGMCCKKTGSVMAGIAHGVAVIVHIAFFIVMWVLESFNFVRMLIGVVTCIQCQRLIFHCMTALMLTREFK
7ex4_F VLFFCMGMSCCX TGSVMAGIAHGVAVIVHIAFFIVMWVLESFNFVRMLIGVVTCIQCQRLIFHCMTALMLTRX
7ex4_F
7yuy_F VLFFCMGMSCCX TGSVMAGIAHGVAVIVHIAFFIVMWVLESFNFVRMLIGVVTCIQCQRLIFHCMTALMLTRX
7yuy_F

```
                          1790      1800      1810      1820      1830      1840      1850      1860      1870
CAF1573557.1         NDHANTAFWTGKWYSTGLGYMAWTQPTRELTAKVIELSEFAADFVLGHVILIFQLPVICIPKIDKFHSIMLFWLKPSRQIRPPIY
KAJ1045660.1         NDHANTAFWTGKWYGKGMGYMAWTQPSRELTAKVIELSEFAADFVLGHVILICQLPLIIIPKIDKFHSIMLFWLKPSRQIRPPIY
KAJ1538062.1         NDHANTAFWTGKWYGKGMGYMAWTQPSRELTAKVIELSEFAADFVLGHVILICQLPLIIIPKIDKFHSIMLFWLKPSRQIRPPIY
KAJ1538062.1_S643P   NDHANTAFWTGKWYGKGMGYMAWTQPSRELTAKVIELSEFAADFVLGHVILICQLPLIIIPKIDKFHSIMLFWLKPSRQIRPPIY
7ex4_F                                       TQPSRELTAKVIELSEFAADFVLGHVILICQLPLIIIPKIDKFHSIMLFWLKPSRQIRPPIY
7ex4_F                                       THHHHHHHHHHHHHHHHHHHHHHHHHHHHHHHTHHHSHSHHHHHHHHHHHHSSHTSSSSTSS
7yuy_F                                       TQPSRELTAKVIELSEFAADFVLGHVILICQLPLIIIPKIDKFHSIMLFWLKPSRQIRPPIY
7yuy_F                                       THHHHHHHHHHHHHHHHHHHHHHHHHHHHHHHHHHHSHSHHHHHHHHHHHHSSTTSSSSTTS

                     1871      1880      1890      1900      1910      1920      1930      1940      1950
CAF1573557.1         SLKQARLRKRMVRRYCSLYFLVLIIFAGCIVGPAVASAHVSKDLGSGLKDTFHNLVQPRNVSNNDTGFQMSTYKSHYYTHTPSLK
KAJ1045660.1         SLKQSRLRKRMVKKYCSLYFLVLAIFAGCIIGPAVASAKIHDQIGDSLTGVVHNLFQPVNTTNNDTGFQMSTYQSHYYTHTPSLK
KAJ1538062.1         SLKQTRLRKRMVKKYCSLYFLVLAIFAGCIIGPAVASAKIHKHIGDSLDGVVHNLFQPINTTNNDTGSQMSTYQSHYYTHTPSLK
KAJ1538062.1_S643P   SLKQTRLRKRMVKKYCSLYFLVLAIFAGCIIGPAVASAKIHKHIGDSLDGVVHNLFQPINTTNNDTGSQMSTYQSHYYTHTPSLK
7ex4_F               SLKQTRLRKRMVKKYCSLYFLVLAIFAGCIIGPAVASAKIHKHIGDSLDGVVHNLFQPINTTNNDTGSQMSTYQSX
7ex4_F               SHHHHHHHHHHHHHHHHHHHHHHHHHHHHHHHHHHHHHSHHSHSHSSTTHSTHHSTSHSSSTTTSTHSHSSHTTD
7yuy_F               SLKQTRLRKRMVKKYCSLYFLVLAIFAGCIIGPAVASAKIHKHIGDSLDGVVHNLFQPINTTNNDTGSQMSTYQSX
7yuy_F               SHHHHHHHHHHHHHHHHHHHHHHHHHHHHHHHHHHHHHSHSSSTSSSTTHSTHHSHSHTSSHTTSTHSHSSHTTD

                          1960
CAF1573557.1         TWSTIK
KAJ1045660.1         TWSTIK
KAJ1538062.1         TWSTIK
KAJ1538062.1_S643P   TWSTIK
```
